# Navigation through high-dimensional chemical space: discovery of Ba_5_Y_13_[SiO_4_]_8_O_8.5_ and Ba_3_Y_2_[Si_2_O_7_]_2_†

**DOI:** 10.1039/d4sc04440a

**Published:** 2024-09-12

**Authors:** Nataliya L. Gulay, Marco Zanella, Craig M. Robertson, Daniel Ritchie, Manel Sonni, Matthew A. Wright, Jon A. Newnham, Cara J. Hawkins, Jayne Whitworth, Bhupendra P. Mali, Hongjun Niu, Matthew S. Dyer, Christopher M. Collins, Luke M. Daniels, John B. Claridge, Matthew J. Rosseinsky

**Affiliations:** a Department of Chemistry, Materials Innovation Factory, University of Liverpool 51 Oxford Street Liverpool L7 3NY UK M.J.Rosseinsky@liverpool.ac.uk; b Leverhulme Research Centre for Functional Materials Design, Materials Innovation Factory, University of Liverpool 51 Oxford Street Liverpool L7 3NY UK

## Abstract

Two compounds were discovered in the well-studied BaO–Y_2_O_3_–SiO_2_ phase field. Two different experimental routines were used for the exploration of this system due to the differences of synthetic conditions and competition with a glass field. The first phase Ba_5_Y_13_[SiO_4_]_8_O_8.5_ was isolated through a combination of energy dispersive X-ray spectroscopy analysis and diffraction techniques which guided the exploration. The second phase Ba_3_Y_2_[Si_2_O_7_]_2_ was located using iterative algorithmic identification of target compositions. The structure solution of the new compounds was aided by continuous rotation electron diffraction, and the structures were refined against combined synchrotron and neutron time-of-flight powder diffraction. Ba_5_Y_13_[SiO_4_]_8_O_8.5_ crystallizes in *I*4̄2*m*, *a* = 18.92732(1), *c* = 5.357307(6) Å and represents its own structure type which combines elements of structures of known silicates embedded in columns of interconnected yttrium-centred polyhedra characteristic of high-pressure phases. Ba_3_Y_2_[Si_2_O_7_]_2_ has *P*2_1_ symmetry with a pseudo-tetragonal cell (*a* = 16.47640(4), *b* = 9.04150(5), *c* = 9.04114(7) Å, *β* = 90.0122(9)°) and is a direct superstructure of the Ca_3_BaBi[P_2_O_7_]_2_ structure. Despite the lower symmetry, the structure of Ba_3_Y_2_[Si_2_O_7_]_2_ retains disorder in both Ba/Y sites and disilicate network, thus presenting a superposition of possible locally-ordered fragments. Ba_5_Y_13_[SiO_4_]_8_O_8.5_ has low thermal conductivity of 1.04(5) W m^−1^ K^−1^ at room temperature. The two discovered phases provide a rich structural platform for further functional material design. The interplay of automated unknown phase composition identification with multiple diffraction methods offers acceleration of the time-consuming exploration of high-dimensional chemical spaces for new structures.

## Abstract 2

Using a combination of experimental and computational tools, two compounds were discovered in the well-studied BaO–Y_2_O_3_–SiO_2_ phase field.

## Graphical abstract

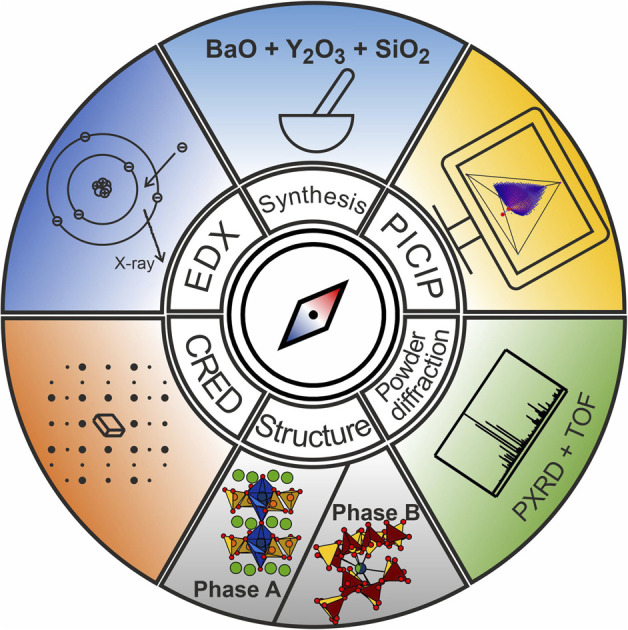

## 1. Introduction

The discovery of new materials has been a driving force for scientific and technological progress.^1^ Recently, there has been a clear trend of involving various artificial intelligence- and machine learning-based technologies to accelerate this process on different levels.^2–5^ There are successful examples that apply a combination of computational techniques narrowed by domain-specific knowledge.^6^ AI and machine learning have been implemented in the discovery process,^7,8^ with arising challenges of implementing a completely human-free experimental workflow identified.^9^ For example, the presence of structural disorder (often only partially resolved) and the accommodation of continuously varying compositions in solid solutions is challenging to treat computationally. The augmentation of traditional exploratory synthesis methods with techniques and tools created to facilitate exploration has proved to be a successful approach for many different classes of materials,^10–12^*e.g.*, intermetallics^13,14^ or electrode materials.^15^

In the case of solid-state systems, exploration can be also hindered by the need for extreme or highly specific experimental conditions (high temperature or pressure, long reaction times *etc.*). A new material may form at conditions that differ from the typical norm for proximal phases or common structures, and despite the advances in prediction of potential synthesizability of theoretical materials^16^ researchers in practice often have to screen multiple synthetic pathways. There have been attempts to develop high-throughput synthetic techniques applicable to solid-state reactions;^17–19^ however, they cannot be applied for all systems given differences in solubility and reactivity within different solids. Another challenge arises from the higher dimensionality of unexplored chemical space as, due to multiple competing reactions in the multicomponent systems, some intermediate phases might be left undiscovered.^20^ There have been developments of *in situ* studies of phase formation in different systems,^21–23^ but such studies are generally unavailable once the reaction temperature becomes too high. Therefore, investigation of a solid-state system may require a combination of different exploration approaches in order to maximize the success rate. The discovery of new phases in high-dimensional chemical spaces is time-consuming and new tools and appropriate workflows that combine and thus build on existing tools are required to accelerate this process.

In this paper we showcase two distinct workflows for materials discovery, one which combines established pertinent experimental techniques and one that incorporates a new computational tool. These enable the isolation and study of two structurally complex materials. We aim to create new structures by combining features of established ones, assisted by the increase in the number of coordination chemistries displayed by the constituent elements. In order to accelerate the investigation of phases at boundaries between known structure types, we combine traditional experimental routes with an iterative algorithmic identification of target compositions based on results from powder X-ray diffraction in order to efficiently target the composition corresponding to the new structure, demonstrating the role of computational analysis beyond high-throughput DFT and AI in the experimental realisation of materials (*i.e.*, in materials discovery). This process was followed by a combination of different diffraction techniques which lead to efficient structure solution.

**Fig. 1:**
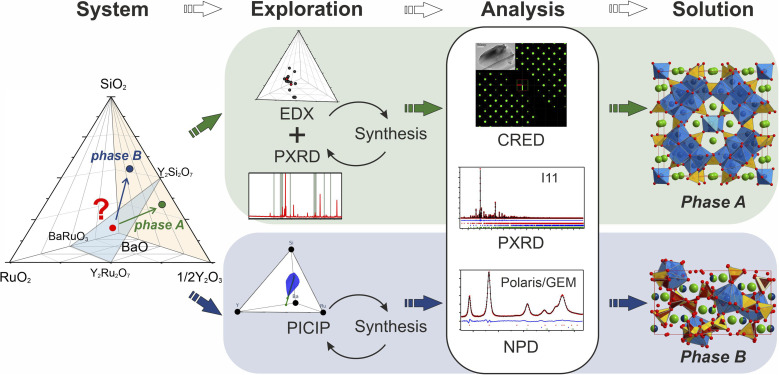
Schematic description of the exploration of the BaO–Y_2_O_3_–SiO_2_–RuO_2_ chemical space. Given an initial synthetic samples (red dot), two routes were used to isolate phases A and B. The first combines the established techniques of EDX with PXRD to identify compositions likely to contain higher fractions of the target phase. The second applies the computational tool PICIP to isolate the crystalline phase that coexists with a competing amorphous field. PICIP uses phase fractions of known phases in PXRD patterns to propose the next compositions to investigate on the basis of the lever rule and uncertainties in refined phase fractions.

The experimental work began with the interphase between well-known materials: the perovskite BaRuO_3_,^24^ the pyrochlore-type Y_2_Ru_2_O_7_,^25^ and Y_2_Si_2_O_7_ ^26^ which was chosen to increase potential structural complexity by introducing the tetrahedral silicon coordination environment. This interphase is placed within the five-dimensional Ba–Y–Si–Ru–O chemical space that so far has no quinary phases reported in the inorganic compound databases.^27,28^ However, within the lower four-dimensional borders of this space, there have been reports on the perovskites Ba_2_YRuO_6_,^29^ Ba_3_YRu_2_O_9_,^30^ and BaY_0.33_Ru_0.67_O_3_ ^31^ for the Ba–Y–Ru–O space, and the silicates Ba_9_Y_2_[SiO_4_]_6_,^32^ BaY_2_Si_3_O_10_,^33^ BaY_4_Si_3_[Si_2_O_7_]O_10_,^34^ and BaY_16_Si_4_O_33_ ^35^ obtained by conventional solid state reactions, which proves that there is potential reactivity of the components. Beginning with solid state syntheses at compositions that span the phase diagrams, unidentified patterns were observed that later led to the discovery of two compounds (referred to as phase A and B throughout the text). In both cases, no ruthenium was incorporated into the structures, and the phases further expand the knowledge of an already populous BaO–Y_2_O_3_–SiO_2_ phase field (Table 1). Phase A was isolated *via* iterative exploratory synthesis based on elemental analysis. Phase B was harder to locate since it is situated near the edge of the glass forming region in the BaO–Y_2_O_3_–SiO_2_ system^37^ and was isolated with the aid of the automated phase isolation software (Probabilistic Isolation of Crystalline Inorganic Phases, or PICIP) that uses overall composition and the diffraction-determined ratios of known phases to direct the selection of compositions in subsequent synthetic reactions (see Fig. 1). The structures of these new phases were solved directly using continuous rotation electron diffraction (CRED) and refined against combined high-resolution synchrotron X-ray diffraction (SPXRD) and time-of-flight neutron diffraction data (TOF). The complementary exploration routes leading to these two phases, their structures and properties are described herein.

**Table 1:** Crystallographic details of the Ba–Y silicate phases for which crystal structures were solved and reported

Phase	Space group	Lattice parameters	Ref.
Ba_9_Y_2_[SiO_4_]_6_	*R*3̄	*a* = 10.02892 Å	36
		*c* = 22.16790 Å	
Ba_3_Y_2_[Si_2_O_7_]_2_ (phase B)	*P*2_1_	*a* = 16.47640(4) Å	This work
		*b* = 9.04150(5) Å	
		*c* = 9.04114(7) Å	
		*β* = 90.0122(9)°	
BaY_2_Si_3_O_10_	*P*2_1_/*m*	*a* = 5.399 Å	33
		*b* = 12.179 Å	
		*c* = 6.852 Å	
		*β* = 106.37°	
BaY_4_[Si_2_O_7_][Si_3_O_10_]	*P*2_1_/*m*	*a* = 5.532 Å	34
		*b* = 19.734 Å	
		*c* = 6.868 Å	
		*β* = 106.53°	
Ba_5_Y_13_[SiO_4_]_8_O_8.5_ (phase A)	*I*4̄2*m*	*a* = 18.92732(1) Å	This work
		*c* = 5.357307(6) Å	
BaY_16_Si_4_O_33_	*P*2_1_/*c*	*a* = 9.1095 Å	35
		*b* = 18.7306 Å	
		*c* = 18.3105 Å°	
		*β* = 109.008	

## 2. Results and discussion

### 2.1. Isolation and structure solution of phase A: Ba_5_Y_13_[SiO_4_]_8_O_8.5_

#### 2.1.1. Phase field exploration

We began the exploration of the BaO–Y_2_O_3_–SiO_2_–RuO_2_ chemical space with the BaRuO_3_–Y_2_Ru_2_O_7_–Y_2_Si_2_O_7_ phase field (highlighted in green in Fig. 1). The detailed description of the experimental methods used is given in SI1† while the main steps will be outlined below. The space was sampled initially through four evenly spaced compositions (see Fig. S1†), and different synthetic temperatures (1273–1573 K) were used to maximize exploration efficiency. Most of the samples contained mixtures of known phases, however two of the powder samples that were heated to 1573 K presented diffraction patterns which could not be assigned to any of the known structures. The unknown peaks were identified, and further investigation has led to isolation of two phases: Ba_5_Y_13_[SiO_4_]_8_O_8.5_ (A) and Ba_3_Y_2_[Si_2_O_7_]_2_ (B). During the experimental exploration, RuO_2_ wasn't incorporated into these structures and formed BaRuO_3_ or Y_2_Ru_2_O_7_ by-products instead (Table S1†).

Phase A was discovered using an iterative diffraction-chemical analysis route marked in green in Fig. 1: EDX analysis of the sample that contained the unknown phase guided the selection of composition for further synthetic exploration. This step was repeated until the highest yield of the new material was achieved. It was not possible to grow suitable single crystals, so the polycrystalline sample that contained the highest phase fraction of the unknown phase A (corresponding to a nominal composition of Ba_16_Y_50_Ru_4_Si_29_O_157_) was studied by means of different diffraction techniques. Continuous rotation electron diffraction enabled analysis of particles which were otherwise too small for single crystal X-ray diffraction,^38^ and its combination with consecutive energy dispersive X-ray spectroscopy (EDX) analysis enabled identification of particles with the symmetry and composition of the new phase. The SPXRD and neutron TOF studies were combined to provide the widest range of diffraction information for the polycrystalline sample. A combination of these techniques resulted in structure solution and refinement of the composition as Ba_5_Y_13_[SiO_4_]_8_O_8.5_. The final synthesis of the sample with this nominal composition provided the highest purity sample. The composition of new phase A is located away from the initial BaRuO_3_–Y_2_Ru_2_O_7_–Y_2_Si_2_O_7_ boundary and the final product contained no ruthenium in the structure, as confirmed by chemical analysis. Details on the samples, their compositions and analysis are given in SI2.†

#### 2.1.2. Symmetry analysis and structure solution

**Fig. 2:**
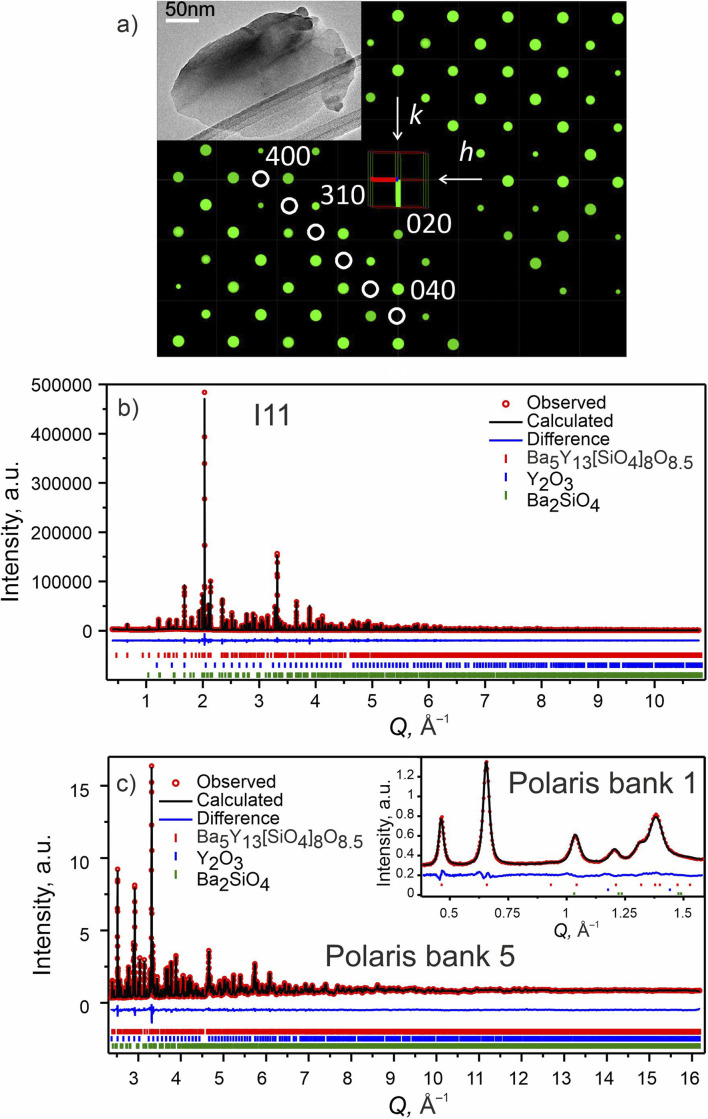
Phase A: (a) slice of the 3D reciprocal space along the *hk*0 plane obtained from CRED data. Systematic absences consistent with *I*4̄2*m* space group are highlighted with white circles. (Inset) TEM microphotograph of one of the measured particles. (b) Rietveld refinement of the model from CRED against synchrotron XRD data (I11, diamond light source, MAC detector, *λ* = 0.82387 Å), and (c) TOF neutron diffraction data (Polaris Bank 5, ISIS). The inset shows the fit to Polaris Bank 1. Bragg reflections for Ba_5_Y_13_[SiO_4_]_8_O_8.5_ (93.55 wt%), Y_2_O_3_ (4.84 wt%) and Ba_2_SiO_4_ (1.61 wt%) are shown as red, blue and green ticks, respectively. Combined Rietveld refinement of Ba_5_Y_13_[SiO_4_]_8_O_8.5_ (*I*4̄2*m*, *Z* = 2) resulted in *R*_wp_ = 3.68% and *χ*^2^ = 3.05 for 154 refined parameters (refinement details and all profiles are given in Table S4†).

The unknown peaks in the SPXRD pattern with the composition of Ba_16_Y_50_Ru_4_Si_29_O_157_ were indexed to a tetragonal unit cell (*a* = 18.92732(1), *c* = 5.357307(6) Å) using TOPAS Academic V7.^39^ The reflections were consistent with a body-centred unit cell with *mmm* Laue class. The experimental pattern was described using Le Bail profile fit, and these data was processed by a charge flipping algorithm within the SuperFlip^40^ tool of the Jana2020 package^41^ to obtain a preliminary structural model. The two best initial solutions corresponded to models with *I*4/*mmm* and *I*4̄2*m* space group symmetry, however, both of the models contained additional oxygen and silicon sites with unreasonable interatomic distances and poorly fitted the experimental data. Therefore, the particles of this sample were analysed by means of CRED (selected sections of reciprocal space and systematic absences analysis are given in SI3†), which by means of direct methods yielded a much cleaner model with clearly recognisable atomic environments (Fig. S12†) consistent with *I*4̄2*m* space group (Fig. 2a). No signs of possible superstructure reflections have been observed. Some atomic sites had to be populated with different species to satisfy geometric and coordination restrictions, but the final model corresponded to a charge-balanced formula Ba_10_Y_26_Si_16_O_81_, which is in good agreement with the composition revealed by EDX (average of Ba 10.72 ± 0.98: Y 23.82 ± 1.95: Si 16 ± 1.65 from the particles measured by CRED, Fig. S1b†). The refinement of CRED data has converged with a final *R*_int_ of 21.78% (Table S3†) which is in line with recent literature values.^42^ The content of Ru from EDX analysis was lower than the detection limit, and further synthetic experiments proved that no Ru is incorporated in the structure of phase A.

To accurately refine the positions and occupancies of light silicon and oxygen atoms, the sample was studied by means of TOF neutron diffraction. The diffraction patterns were fitted, and the structures refined using TOPAS Academic V7,^39^ and the final solution was obtained using combined refinement of high resolution SPXRD and TOF neutron diffraction data.^43^

Selected Rietveld refinements performed against SXRD and neutron diffraction data are shown in Fig. 2b and c, while all profiles are shown in Fig. S13.† During the multi-pattern refinement, the unit cell parameters were fixed according to the highest resolution data (SPXRD), and other structural variables were refined using all datasets. The unit cell contains three yttrium, two barium, two silicon and seven oxygen sites. The occupancy of the Ba1 site refined to 50% within error based on the combined refinement, and the occupancy of the O7 site was fixed at 50% to charge balance the formula. A possibility to have a fully occupied oxygen site charge-balanced by hydrogen forming OH group was tested but the IR spectrum showed no signs of these groups (Fig. S26†). Analysis of the difference Fourier maps revealed that the Ba1 and Y3 atoms are displaced along the *c* axis from the 4d and 2b positions respectively, therefore their occupancy parameters were reduced by half to respect site multiplicities. After obtaining a stable solution, the unit cell parameters were refined as a final step. Crystallographic and refinement details for Ba_5_Y_13_[SiO_4_]_8_O_8.75_ are given in Table S4.† Refined atomic parameters are listed in Table S5,† while the interatomic distances for the atoms can be found in Table S6.†

To test the possibility of ordering at lower temperature, the SPXRD was also measured at 100 K. The Rietveld refinements of such diffraction data showed the expected contraction of the unit cell (*a* = 18.90366(2), *c* = 5.351310(9) Å, Fig. S14†) but revealed no superstructure reflections, and the atomic parameters deviate only slightly from those at room temperature (Table S7†).

#### 2.1.3. Structure of Ba_5_Y_13_[SiO_4_]_8_O_8.5_ (phase A)

**Fig. 3:**
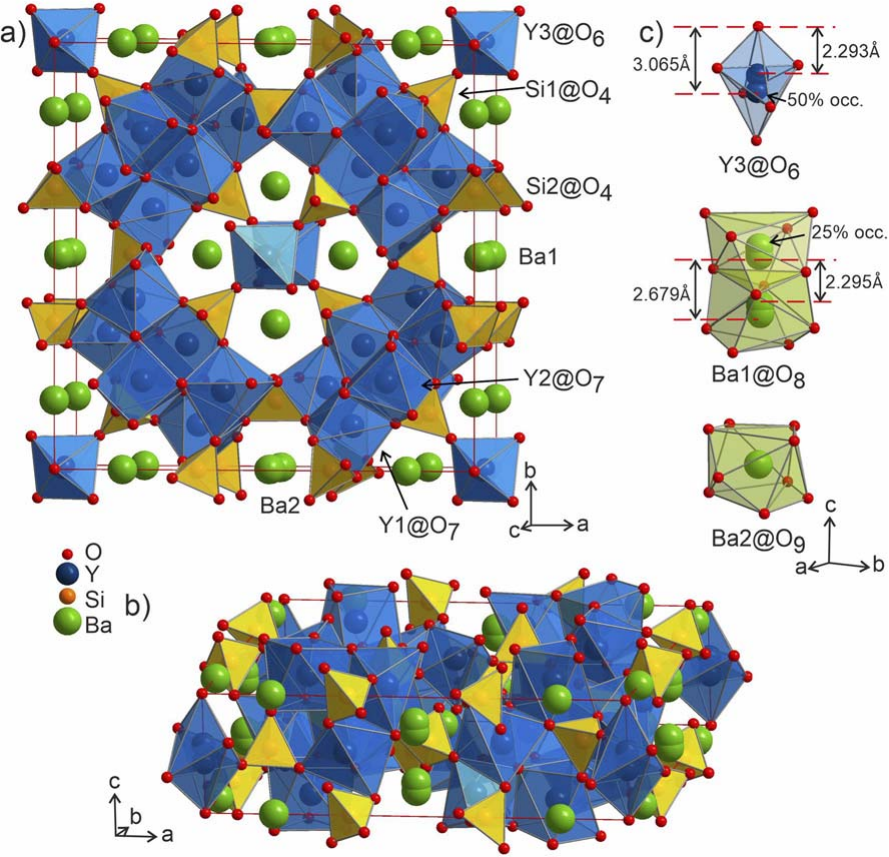
(a and b) Structure of phase A Ba_5_Y_13_[SiO_4_]_8_O_8.5_ with silica tetrahedra and yttrium-centred polyhedra drawn in yellow and blue, respectively. (c) The Ba1 and Ba2-centered polyhedra (green shading) drawn oriented along the *c* axis. Y3@O_6_ octahedron is shown along the same direction, shaded in a light-blue colour to provide a better view on disordered atomic site.

Ba_5_Y_13_[SiO_4_]_8_O_8.5_ (Phase A) crystallizes in space group *I*4̄2*m*, Pearson code tI142, Wyckoff sequence j^5^i^4^hf^2^ea that represents its own structure type which is not reported in ICSD (version 5.2.0, Data Release 2024.1)^28^ or Pearson's^27^ databases. A phase with this space group and cell has been mentioned in conference proceedings by Wierbocka-Wieczorek *et al.*^44^ but no detailed crystallographic data or explanation was given in that publication. It has been recently followed by Yamane *et al.*^45^ in which a more Ba-rich phase (Ba_10.22_Y_26_Si_16_O_81.22_) with an incommensurate composite structure was reported to form at 1873 K. In contrast to the majority of known compounds in the Ba–Y–Si–O chemical space, phase A contains barium atoms coordinated *via* Si@O_4_ tetrahedra and condensed yttrium-centred polyhedra representing a mixed silicate-oxide (see Fig. 3). Ba1 and Ba2 atoms are coordinated by 8 and 9 oxygens, respectively, and all Ba–O distances (2.584–3.189 Å, Table S6†) are comparable to those reported for the rocksalt type barium(ii) oxide (2.752 Å)^46^ and other Ba–Y–Si–O materials, *e.g.*, Ba_9_Y_2_[SiO_4_]_6_ ^36^ (Ba–O 2.535–3.321 Å). The disordered Ba1 atoms (Fig. 3c) show rather short Ba1–Ba1 distances of 2.295 and 2.679 Å (the latter being half of the unit cell *c* dimension) between each pair of atoms; however, the Ba1–O1 distances (2.584–3.189 Å) lie within the ranges known for other barium oxide compounds.^27^ These sites sum to a total of 50% occupancy, therefore we can assume a statistical chance of locally observing only one barium atom in one of the four 8h sites per silica-coordinated column in the unit cell. Similar disordered models with proximal half-occupied barium sites have been used to describe structures of the mineral muirite (Ba_10_(Ca,Mn,Ti)_4_Si_8_O_24_(Cl,OH,O)_12_·4H_2_O, which has Ba3–Ba3 distances of 2.758 and 2.877 Å,^47^ as well as the synthetic titanosilicate (Ba,Sr)_4_Ti_6_Si_4_O_24_·H_2_O (with Ba2–Ba2 distances of 2.866 Å).^48^

Both types of silicon atoms form nearly perfect tetrahedra; the Si1–O (1.617–1.647 Å) and Si2–O (1.629–1.632 Å) distances are similar to those reported for α-quartz (1.603–1.610 Å).^49^ Slight asymmetry for the Si1@O_4_ tetrahedra can be attributed to the disordered Y3 and O7 sites which impose spatial strain on the neighbouring atoms hence causing slight deformation of their coordination polyhedra. There are two types of yttrium coordination in the structure: Y1 and Y2 atoms are situated inside distorted trigonal prisms with one additional coordinating oxygen atom (CN = 7) while disordered Y3 sites are surrounded by distorted octahedra (CN = 6). Y1–O and Y2–O distances of 2.212–2.521 Å have a slightly broader range compared to those observed in structures of other similar compounds, *e.g.*, BaY_4_[Si_2_O_7_][Si_3_O_10_]^34^ (2.220–2.381 Å). Due to displacement from the high symmetry 
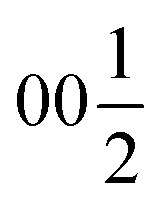
 position (Fig. 3c), the coordination of the Y3 atom becomes distorted resulting in a variation of Y–O distances from 2.007 to 3.065 Å with the longest Y3–O7 distance exceeding the average bond value, which likely reflects weaker interaction with the half-occupied O7 site.

**Fig. 4:**
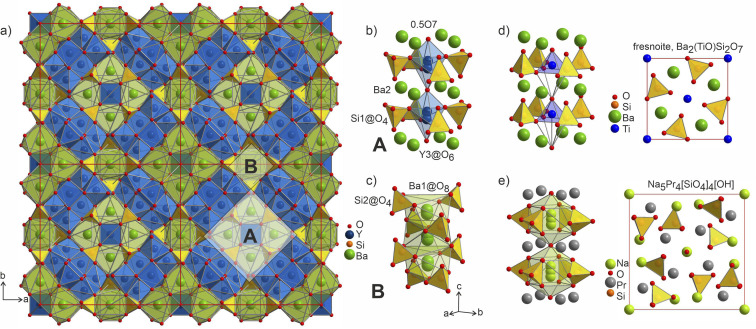
(a) 2*a* × 2*b* projection of the unit cell of Ba_5_Y_13_[SiO_4_]_8_O_8.5_ showing the arrangement of the silica tetrahedral network. Two motifs that form the structure are marked as A and B. (b) Motif A: disordered Y3 and O7 sites within the columns formed by Ba1 atoms and Si1@O_4_ tetrahedra. (c) Motif B: disordered Ba1 sites with square antiprismatic coordination realized by Si2@O_4_ tetrahedra. (d) Structure of fresnoite, Ba_2_(TiO)Si_2_O_7_ ^50^ with unusual square pyramidal coordination of titanium atoms. Note that upon inclusion of the oxygen from the edge of bottom pyramid, the titanium atoms become octahedrally coordinated which resembles the distorted Y3@O_6_ octahedra from the fragment A (emphasised by grey lines). (e) Structure of Na_5_Pr_4_[SiO_4_]_4_[OH] that contains disordered Na2 sites situated within bicapped square prisms.^51^ Different distribution of the disordered atoms results in a square-prismatic coordination of these sites in contrast to Ba1-centred square antiprisms in fragment B.

Despite representing its own structure type, Ba_5_Y_13_[SiO_4_]_8_O_8.5_ contains structural fragments similar to those observed for known compounds. When viewed in the *ab* plane, the silicon-oxide tetrahedral network forms two types of distinct motifs arranged in a checkerboard-like pattern (A and B in Fig. 4a). The first one (A) coordinates the half-occupied O7 sites at cell edges and Y3 atoms, displaced from the high symmetry 
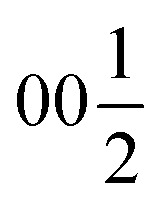
 position along the *c* axis (Fig. 4b). The O7 atoms lie within the plane of neighbouring Ba2 atoms, while disordered Y3 sites are surrounded by four Si1@O_4_ tetrahedra. Surprisingly, this first motif is remarkably similar to the structure of the mineral fresnoite, Ba_2_(TiO)Si_2_O_7_ ^50^ (Fig. 4d), that features similar layers of barium atoms and silicon tetrahedra. In fresnoite, the silicon tetrahedra surround titanium atoms with rather unusual square pyramidal coordination which upon inclusion of an oxygen atom from the next layer resemble the distorted Y3@O_6_ octahedra observed in phase A (emphasized by grey lines at Fig. 4d). The main difference between these structural fragments is the orientation of the silicon tetrahedra: in fresnoite, their apexes are oriented in the same direction as the one of the titanium-centred pyramids, while they are alternately oriented in opposing directions in phase A. Another motif (B) in the structure of Ba_5_Y_13_[SiO_4_]_8_O_8.5_ consists of disordered Ba1 sites in face-sharing square-antiprismatic coordination environments further coordinated by alternating pairs of Si2@O_4_ tetrahedra along the *c* axis (Fig. 4c). This fragment can be compared with the structure of Na_5_Pr_4_[SiO_4_]_4_[OH] which features disordered Na2 sites coordinated by silicon tetrahedra (Fig. 4e).^51^ However, due to a different orientation of Si@O_4_, the sodium atoms are situated inside bicapped square prisms while fragment B contains square-antiprismatic coordination of Ba1 sites. Furthermore, the sodium sites in the structure of Na_5_Pr_4_[SiO_4_]_4_[OH] are separated with layers of praseodymium atoms while the fragment B in the structure of Ba_5_Y_13_[SiO_4_]_8_O_8.5_ has no clear separation due to more uniform distribution of 25% occupied Ba1 atoms along the columns.

**Fig. 5:**
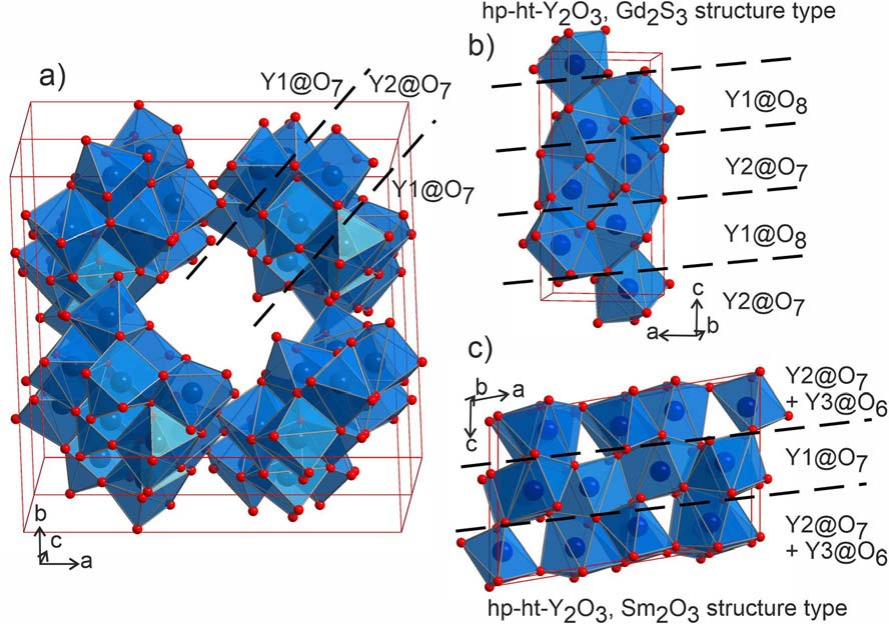
(a) Projection of a 1 × 1 × 2 unit cell of Ba_5_Y_13_[SiO_4_]_8_O_8.5_ to emphasize the channels formed by Y1- and Y2-centred polyhedra. Condensed motifs of edge-sharing Y1@O_7_/Y2@O_7_/Y1@O_7_ polyhedra resemble the fragments of the structures of high-pressure and high-temperature modifications of yttria: (b) Gd_2_S_3_ structure type^52^ of Y1@O_8_/Y2@O_7_ alternating layers and (c) Sm_2_O_3_ structure type with (Y2@O_7_ + Y3@O_6_)/Y1@O_7_/(Y2@O_7_ + Y3@O_6_) layers.^53^

The distorted Y1- and Y2-centered polyhedra connect *via* common edges forming a peculiar condensed pattern, that occupies the voids within the silicon–oxygen network (see Fig. S15†). Pairs of Y1@O_7_ “sandwich” pairs of Y2@O_7_ polyhedra (see Fig. 5a), and these layers are arranged on top of each other forming columns along the *c* axis. Surprisingly, such layered packing resembles those observed for known high-temperature and high-pressure modifications of yttria which adopt the Sm_2_O_3_ and Gd_2_S_3_ structure types (Fig. 5b and c). The latter structures also feature broader ranges of Y–O distances of 2.188–2.699 Å and 2.187–2.789 Å for Sm_2_O_3_- and Gd_2_S_3_-type yttria, respectively, which are comparable to the distances in the structure of Ba_5_Y_13_[SiO_4_]_8_O_8.5_. The sevenfold-coordinated atoms of yttrium have been also observed in the structure of another mixed oxide-silicate BaY_16_Si_4_O_33_,^35^ but in that case they are distributed within the structure alternating with the sixfold-coordinated sites.

The diversity of coordination in the structure of Ba_5_Y_13_[SiO_4_]_8_O_8.5_ indicates the potential of this structure for function incorporation. The half-occupied O7 atoms signal the possibility of ionic conductivity induced by this disorder. The presence of rigidly-coordinated yttrium and barium sites indicates that this material has a potential as a phosphor host similarly to many other known silicates.^54^

### 2.2. Isolation and structure solution of phase B: Ba_3_Y_2_[Si_2_O_7_]_2_

#### 2.2.1. Phase field exploration

The experimental route that led to discovery of phase B is highlighted in blue in Fig. 1. During the exploration of the BaRuO_3_–Y_2_Ru_2_O_7_–Y_2_Si_2_O_7_ phase field, a newly-developed tool Probabilistic Isolation of Crystalline Inorganic Phases (PICIP) was tested for the first time. As input, PICIP takes the nominal overall composition for each sample and the relative amounts of known crystalline phases present in that sample as estimated from quantitative X-ray diffraction-based phase analysis. PICIP assumes uncertainties in these relative amounts of known phases and predicts a probability density over a region of phase space evaluating the expected average composition of the unknown phases present. This probability density can be aggregated over multiple samples with different nominal compositions to improve the overall accuracy, effectively triangulating the average composition of the unknown phases. PICIP thus automates application of the lever rule in high-dimensional chemical space taking experimental uncertainties into account, in order to accelerate identification of compositions affording unknown structures. A detailed description of how PICIP works is provided in the ESI (page 3†).

**Fig. 6:**
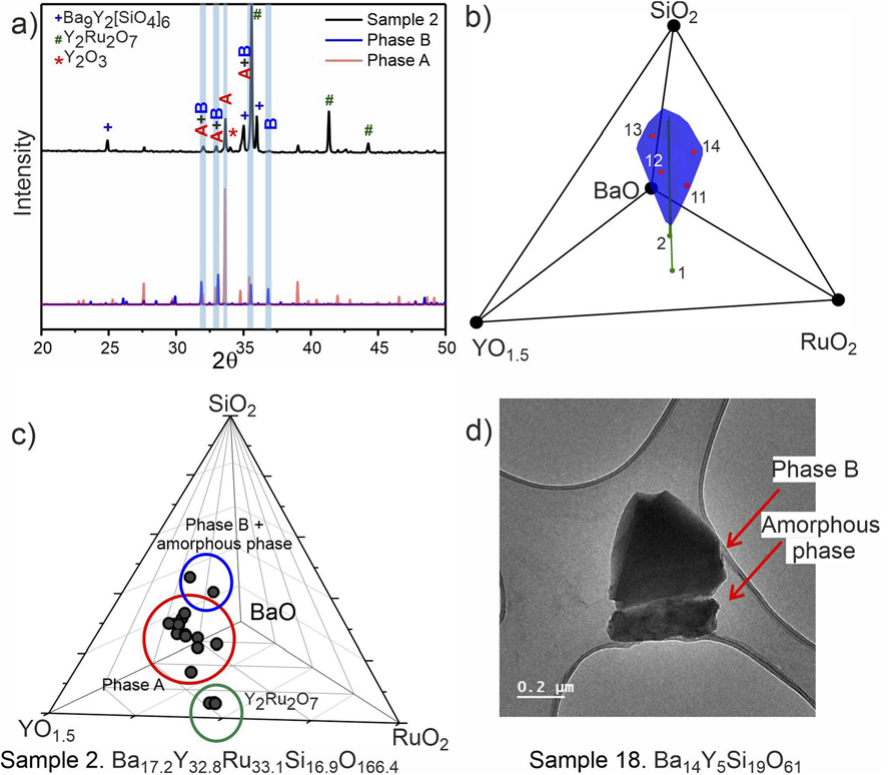
(a) Experimental PXRD pattern (Co Kα_1_ radiation) for the sample 2 (Ba_17.2_Y_32.8_Ru_33.1_Si_16.9_O_166.4_, top), where the unidentified peaks were first located. Calculated patterns for phases A and B (bottom) are rescaled to recreate the phase ratio, with the overlapping strongest reflections marked in blue. (b) The blue cone shows the region which contains 50% of the probability mass computed by PICIP, calculated from samples 1 (Ba_9.2_Y_40.8_Ru_41.4_Si_8.6_O_170.4_) and 2 (Ba_17.2_Y_32.8_Ru_33.1_Si_16.9_O_166.4_). (c) Results of EDX analysis of sample 2 with clusters corresponding to different phases grouped and marked with circles. (d) TEM microphotograph of one of the measured particles from the sample 18 (Ba_14_Y_5_Si_19_O_61_) that consists of two parts with distinct morphology (phase B and amorphous phase).

PICIP was initially tested on two of the first powder samples that presented the unidentified peaks in their diffraction patterns (Fig. 6a). PXRD analysis of samples 1 and 2 revealed mixtures of the known phases Y_2_Ru_2_O_7_ and Ba_9_Y_2_[SiO_4_]_6_ (details in Table S2†) along with reflections from the unknown phases. On the basis of the relative amounts of Y_2_Ru_2_O_7_ and Ba_9_Y_2_[SiO_4_]_6_ in these samples and using a standard deviation of 2 wt% on these amounts, PICIP predicted the residual composition to be in a different region of the Ba–Y–Si–Ru–O chemical space compared to phase A suggesting the presence of an additional unknown phase (Fig. 6b). It should be noted that EDX analysis of sample 2 showed two points in the same region but they did not cluster as well as for phase A (Fig. 6c). From the aggregated probability density an additional four compositions were chosen by PICIP (samples 11–14, Fig. S5a†) and prepared using the same synthetic routine as for the initial batch (details are given in SI1†). The PXRD patterns of these samples revealed a second set of unidentified reflections.

Here it should be noted that some of the studied samples became completely amorphous upon melting if annealed for too long at 1573 K (see Fig. S5† for sample 13, Ba_15_Y_5_Ru_2_Si_18_O_61_). This happened for Ru-deficient and Si-rich compositions, and agrees with the previous reports for the BaO–Y_2_O_3_–SiO_2_ system, where a wide range of compositions in the SiO_2_-rich corner form amorphous glasses upon melting.^37^ The presence of other crystalline and amorphous phases in this compositional region prevented the definitive use of EDX analysis to locate the phase B in contrast to phase A which was readily isolated this way (see Fig. S7†). Therefore, PICIP was used again starting with the relative amounts of known phases BaSiO_3_, Ba_9_Y_2_[SiO_4_]_6_ and BaRuO_3_ in sample 13 (Table S2 and Fig. S5b†) with a 2 wt% standard deviation on these amounts to suggest the next compositions for experimental synthesis.

Since any amorphous phases present will not contribute to the relative amount of known crystalline phases in the sample, their composition will also contribute to the average composition of unknown phases in the sample. Whilst this prevents PICIP from suggesting compositions which have a reduced amount of amorphous phase, PICIP remains able to suggest compositions which minimise the amount of competing known crystalline phases.

Integration of PICIP in the exploratory routine helped obtain a sample with a high yield of phase B with respect to crystalline phases, which was finally sufficient for CRED/EDX studies. Final tuning of the composition and reaction conditions resulted in a sample with the highest purity of phase B as determined by X-ray diffraction (starting composition of Ba_31_Y_23_Si_46_O_157.5_, annealing at 1573 K for 12 h). This composition yields an almost phase-pure powder of phase B with respect to the crystalline phases present (detailed synthetic procedure is described in SI1†). At the same time, it does contain a noticeable amount of amorphous glassy phase. TEM imaging helped to identify the presence of two different particle morphologies, sometimes in the same crystallite, corresponding to phase B and an amorphous phase (Fig. 6d). Similarly to phase A, no ruthenium was necessary for the formation of this phase.

The purest sample of phase B with respect to crystalline content was studied similarly to phase A with CRED, SPXRD and TOF neutron diffraction, which enabled solution and refinement of the structure.

#### 2.2.2. Symmetry analysis and structure solution

The sample with a nominal composition of Ba_31_Y_23_Si_46_O_157.5_ contained the unknown phase referred to as phase B which was at first indexed to a primitive tetragonal lattice (*a* = 16.476, *c* = 9.041 Å) using TOPAS Academic V7.^39^ Similar to phase A, the initial models were developed using both charge-flipping applied to the powder diffraction data and direct methods using CRED data. The outcomes from both methods converged on the space group *P*4_3_2_1_2 with two 8b silicon, one 8b barium, one 4a yttrium, and one 8a mixed barium/yttrium (0.5:0.5) atomic sites. Additionally, the model was populated with seven 8b oxygen sites which coordinate silica atoms forming disilicate groups to result in a charge balanced formula Ba_3_Y_2_Si_4_O_14_. However, after refinement of the structural model against powder diffraction data, it lacked the necessary coordination for heavy atoms and the fit to experimental data required improvement. The higher symmetry model is shown in Fig. S23† for reference (atomic coordinates are listed in Table S13†). We have attempted to lower the symmetry to orthorhombic *P*2_1_2_1_2_1_ space group and to split the disilicate groups which did not resolve the mentioned problems. The diffraction patterns for selected models and statistics from the Rietveld refinements are shown in Fig. S24 and Table S14.†

**Fig. 7:**
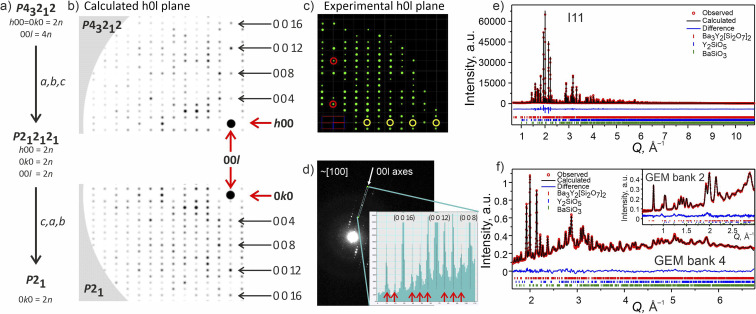
(a) Symmetry reduction route for the structure model of phase B consistent with the systematic absences found while slicing the 3D reciprocal space. More detailed reciprocal space analysis for phase B is given in SI5.† (b) Simulated diffraction pattern for the *h*0*l* plane in higher symmetry *P*4_3_2_1_2 and lower symmetry *P*2_1_ models using the same instrumental parameters for the CRED measurement. The 000 point is marked with a black circle. (c) Cutout of the experimentally obtained *h*0*l* plane with the violation of reflection conditions along the 00*l* axis marked with red circles and the systematic absences along the *h*00 plane marked in yellow (data is merged over 10 particles). (d) TEM frame close to [100] zone axes. The inset shows a line profile along the 00*l* axes plotted from the area included in the cyan rectangle. The violations of the 00*l* = 4*n* reflection condition for *P*4_3_2_1_2 space group are marked with red arrows. (e) Rietveld refinement of against synchrotron XRD data (I11, Diamond Light Source, MAC detector, *λ* = 0.82387 Å), and (f) TOF neutron diffraction data (GEM Bank 4, ISIS). The inset shows the fit to GEM Bank 2. Bragg reflections for Ba_3_Y_2_[Si_2_O_7_]_2_ (96.11 wt%), Y_2_SiO_5_ (2.59 wt%) and BaSiO_3_ (1.30 wt%) are shown as red, blue and green ticks, respectively. Combined Rietveld refinement of Ba_3_Y_2_[Si_2_O_7_]_2_ (*P*2_1_, *Z* = 4) resulted in *R*_wp_ = 5.19% and *χ*^2^ = 2.92 for 413 refined parameters (refinement details and all profiles are given in SI6†).

Attempts to solve the structure directly from CRED data were made using direct methods, intrinsic phasing and charge flipping algorithms implemented in Olex2.^55^ Every attempt failed, which can be attributed to low intensity of some reflections which could not be extracted by REDp or micro ED CrysAlisPro v43. Nevertheless, electron diffraction provided us with a crucial insight into the structure of Ba_3_Y_2_[Si_2_O_7_]_2_. Upon a careful re-examination of different sections of the 3D reciprocal space of the CRED data, violations of the 00*l* = 4*n* and 0*k*0 = 2*n* reflection conditions^56^ of the *P*4_3_2_1_2 space group were observed (Fig. 7b–d). At the same time, clear systematic absences were observed along the *h*00 axis. To satisfy these reflection conditions, the symmetry of the model was lowered from *P*4_3_2_1_2 to its direct subgroup of *P*2_1_ (Fig. 7a). Comparison between the calculated patterns in both symmetries is given in Fig. 7b, and is in a good agreement with the experimental data (Fig. 7c, d, and S5†). The group–subgroup relationship and the atomic parameters evolution is illustrated in Fig. S21 and S22† (oxygen atoms are omitted).

The monoclinic model in the *P*2_1_ space group was successfully refined using combined SPXRD and TOF neutron diffraction data^43^ (*a* = 16.47640(4), *b* = 9.04150(5), *c* = 9.04114(7) Å, *β* = 90.0122(9)°), and its accuracy was evaluated using the maximum entropy method (MEM).^57^ The refined SPXRD and TOF banks 5 and 2 patterns are shown on the right-hand side of Fig. 7 (the featured background of the neutron diffraction spectra is caused by a presence of the amorphous phase). All profiles for Banks 6–2 are shown in Fig. S19.†

The monoclinic model of Ba_3_Y_2_[Si_2_O_7_]_2_ contains four fully occupied barium and two yttrium atoms. Additionally, four sites are occupied by Ba/Y mixtures in different ratios close to 50 : 50. There were 8 initial silicon sites that were coordinated pairwise by oxygen, forming Si_2_O_7_ disilicate groups. This model did not however provide a good fit to the diffraction data and inspection of the difference Fourier maps showed multiple unassigned regions of electron and nuclear density. Given the uniformly disordered nature of the Ba/Y sites, it was decided to split atoms forming the disilicate groups in similar 50 : 50 ratios to model disorder occurring due to the substitution of bigger Ba for smaller Y atoms. This resulted in 4 equivalent pairs of disilicate groups (16 silicon and 56 oxygen sites), which were restrained to retain disilicate geometry. The occupancies of all atoms within each disilicate group were refined as one parameter, and each pair of disilicate groups was restrained to sum to 100% occupancy. Due to the high degree of disorder, the thermal parameters of all silicon and oxygen atoms were constrained to the same value per atomic type. This model yielded a good fit to both SPXRD and neutron diffraction data and provided necessary bonding for the heavy atoms. MEM analysis was applied to the observed synchrotron diffraction data to generate the electron density distribution which supports the split disilicate model (see Fig. S25† featuring “dumbbells” of electron density). The refined formula of Ba_3.03(3)_Y_1.97(4)_Si_4_O_14_ is charge balanced and close to the composition measured by EDX from particles selected for CRED measurement (average of Ba 3.03 ± 0.36: Y 1.42 ± 0.38: Si 4 ± 0.39, Fig. S1b†). Crystallographic and refinement details for Ba_3_Y_2_[Si_2_O_7_]_2_ are given in Table S9.† Refined atomic parameters are listed in Table S10† (atoms forming disilicate groups are listed together for clarity), while the representative interatomic distances for the atoms can be found in Table S11.†

The Rietveld refinements of 100 K synchrotron diffraction data with the same model as obtained at room temperature showed expected contraction of the unit cell (*a* = 16.45139(4), *b* = 9.02912(4), *c* = 9.02884(6) Å, *β* = 90.0104(7)°, Fig. S20†) but revealed no ordering transition, and the atomic parameters deviate only slightly from the room temperature solution (Table S12†).

#### 2.2.3. Structure of Ba_3_Y_2_[Si_2_O_7_]_2_

Ba_3_Y_2_[Si_2_O_7_]_2_ (phase B) crystalizes in space group *P*2_1_ with a pseudo-tetragonal unit cell of data (*a* = 16.47640(4), *b* = 9.04150(5), *c* = 9.04114(7) Å, *β* = 90.0122(9)°), Pearson code mP172, Wyckoff sequence a^86^ and is a direct disordered superstructure of the known structure type of the orthorhombic Cs_3_CaBi[P_2_O_7_]_2_ (*P*2_1_2_1_2_1_).^58^ The group–subgroup relationship is schematically described in Fig. 7a, while a more detailed description of the atomic site evolution is given in Fig. S21 and S22.† Despite the same stoichiometry, the heavy atoms in the structure of Ba_3_Y_2_[Si_2_O_7_]_2_ adopt a different colouring pattern then those in the structure of Cs_3_CaBi[P_2_O_7_]_2_ (Fig. S23†). The caesium sites in the substructure are replaced with a mixture of barium and Ba/Y sites, the calcium sites are substituted by yttrium, while the bismuth positions are occupied by Ba/Y mixtures. Due to different valency of these heavy atoms as well as their radii, the equivalent sites in both structures have different coordination environments. Bonding to a different number of oxygen atoms at each site causes rotation of the disilicate groups in the structure of phase B compared to the diphosphates of Cs_3_CaBi[P_2_O_7_]_2_ and results in lowering of the symmetry of phase B.

**Fig. 8:**
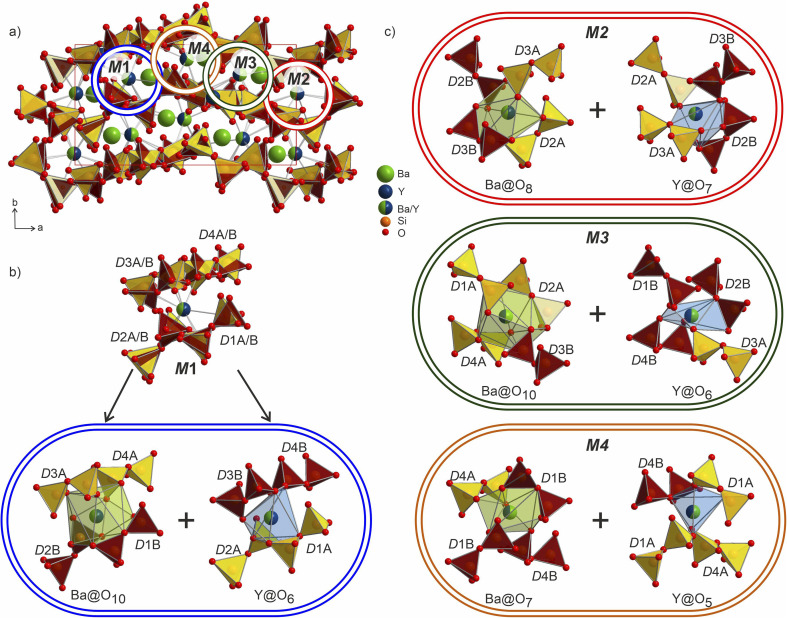
(a) Structure of Ba_3_Y_2_[Si_2_O_7_]_2_ plotted parallel to the *ab* plane. Two sets of disilicate groups are marked in yellow (D1A–D4A) and orange (D1B–D4B) to illustrate site splitting and disorder in the structure. The four crystallographically independent mixed occupied Ba/Y sites M1–M4 are marked with circles. (b) Co-existence of two different coordinations within the environment of the M1 site corresponding to the larger barium and smaller yttrium ions. Split disilicate groups accommodate both environments and provide opportunities for shorter and longer bonds within the locally ordered fragments (surrounded by a blue border). (c) Analogous pairs of larger and smaller environments corresponding to the M2–M4 sites. Colours of borders match the circles in the unit cell (a).

The Ba/Y disorder that is not resolved even in the lower symmetry *P*2_1_ model could be a driving force for the further splitting of the disilicate groups. The network formed by these disilicate groups in the unit cell of Ba_3_Y_2_[Si_2_O_7_]_2_ is shown in Fig. 8. Each of the 4 mixed Ba/Y sites has a non-equivalent coordination to 4 split disilicate groups. Since the mixed sites represent the statistical possibility of the presence of different atomic species, the split disilicate groups model the possibility of having a lower coordination number environment for yttrium atoms and a larger one for barium (corresponding to their ionic radii). Despite the high degree of disorder, such a model gives us a glimpse of the possible local ordering of the atomic species. As exemplified for the mixed site M1 in Fig. 8b, it can be realized either as the larger 10-coordinated environment typical for barium or a smaller 6-coordinated yttrium. Similar decomposition into two different environments for M2–M4 sites is shown in Fig. 8c. These arrangements of disilicate groups representing coordination to two cation environments show possibilities of local ordering within the structure of Ba_3_Y_2_[Si_2_O_7_]_2_. Since there are four split Ba/Y sites and 8 pairs of disilicate groups, one is presented with 32 different options (2^4^ + 2^4^) to populate the unit cell in an orderly manner. Therefore, we must assume that this model corresponds to the average structure of Ba_3_Y_2_[Si_2_O_7_]_2_ and drawing an exact connectivity pattern between Ba/Y and each set of split disilicate groups is beyond the scope of this study. The disorder correlates well with observed physical behaviour of phase B which turns into a glass upon melting. Hence this model is an example of a crystalline structure on the way to become amorphous.

At the same time, there is always a possibility that disorder will resolve itself while moving to a different chemical space and/or different conditions leading to a potential functional material. For example, a Na[LnSiO_4_]-based phosphor forms as a disordered *Pnma* structure above 100 K while ordering with *Imma* symmetry below this point which also causes a change in luminescence performance.^59^ Full or partial substitution of atomic species in the structure of Ba_3_Y_2_[Si_2_O_7_]_2_ may force similar ordering and potentially improve synthetic conditions (*i.e.*, transition to a chemical space without competing glass field).

**Fig. 9:**
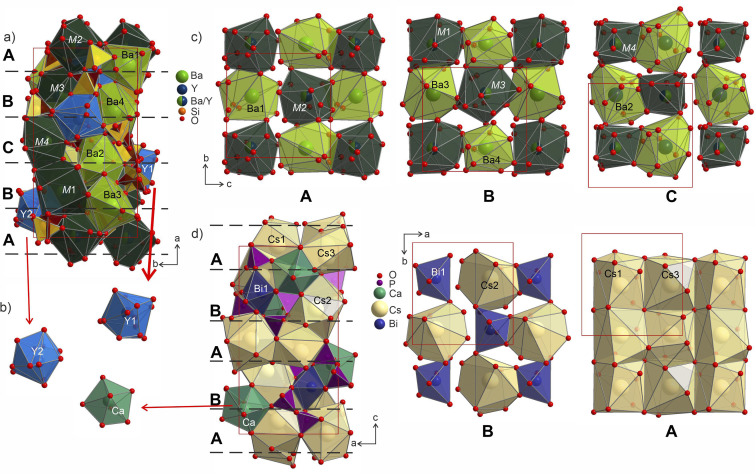
(a) Structure of Ba_3_Y_2_[Si_2_O_7_]_2_ shown parallel to the *ab* plane with Y-, Ba- and M-centred polyhedra drawn in blue, light-green and dark-green, respectively. Two sets of disilicate groups are marked in yellow and brown to illustrate site splitting and disorder in the structure. Three different layers A (c), B (d), C (c) formed by Ba- and M-centred polyhedra are separated with red dashed lines. (b) Coordination of Y1 and Y2 sites in comparison with pentagonal bipyramids of Ca atoms in the structure of Cs_3_CaBi[P_2_O_7_]_2_.^58^ (c) Cut-outs of the layers arranged from Ba- and M-centred polyhedra in a checkerboard-like pattern. Yttrium and silicon coordination is omitted for clarity. The site occupancies for mixed sites M1–M4 are listed in Table S10.† (d) Structure of Cs_3_CaBi[P_2_O_7_]_2_ with two layers formed by Cs- and Bi-centred polyhedra drown on the right.

The individual coordination of the yttrium and barium atoms is hard to describe in terms of simple polyhedra due to coordination by disordered disilicate groups. The arising environments are realized as superpositions of polyhedra that could have been formed locally by the ordered species. This is most evident in comparison to the ordered Cs_3_CaBi[P_2_O_7_]_2_ aristotype. The yttrium atoms coordination forms polyhedra Y1@O_13_ and Y1@O_14_ (shaded in blue in Fig. 9) that in their overall shapes resemble a defect superposition of the pentagonal dipyramids surrounding the calcium atoms in the structure of Cs_3_CaBi[P_2_O_7_]_2_ (Fig. 9b). They show a broad range of Y–O distances from 1.96 to 2.82 Å, which differs from those observed for other ordered Ba–Y silicates like BaY_16_Si_4_O_33_ (Y–O of 2.12–2.62 Å).^35^ The lower values from this region are comparable to those observed for *e.g.*, YBa_5_Cu_2_O_8.5+*x*_ (shortest Y–O of 2.01 Å)^60^ or YBaFeO_4_ (shortest Y–O of 2.09 Å).^61^

The structure of Ba_3_Y_2_[Si_2_O_7_]_2_ contains 4 crystallographically independent barium atoms that are coordinated by 11–14 half-occupied oxygens: Ba1@O_11_, Ba2@O_12_, Ba3@O_14_, and Ba4@O_12_ (shaded in light-green in Fig. 9). These sites are related to Cs3 and Cs2 in the structure of Cs_3_CaBi[P_2_O_7_]_2_ ^58^ which are surrounded by already distorted 9- and 10-vertex polyhedra. The Ba–O distances lie within 2.41–3.19 Å and correlate well with the values reported for other oxides of barium.^19^ The shortest bond of 2.41 Å for Ba4–O7a is slightly lower than those observed in the structure of phase A or reported for other Ba–Y silicates (see previous section). However, similar values have been reported in other systems such as the chloride silicate Ba_5_[SiO_4_]Cl_6_ with Ba2–O2 distances of 2.467 Å ^62^ or a Ba–Fe disilicate Ba_2_Fe[Si_2_O_7_] with Ba–O2 of 2.417 Å.^63^

The mixed occupied sites M1–M2 show a much broader range of Ba–O distances from 1.96 to 3.20 Å and are surrounded by irregularly shaped polyhedra (drawn in dark-green in Fig. 9). These sites show the most striking difference in coordination compared to the aristotype. The M2 and M4 sites arise after splitting of Cs1 site (see group-subgroup scheme in Fig. S21 and S22†); they are surrounded by 13 partially occupied oxygen sites which is comparable to 11 for Cs1 atoms. At the same time, M1 and M3 sites that are equivalent to Bi sites are centred within much larger coordination: M1@O_15_ and M3@O_14_*versus* Bi@O_6_. The distorted coordination of these two atomic sites arises both from superposition of the original distorted octahedra of bismuth atoms and inclusion of further oxygen atoms due to larger radii of the Ba/Y sites. At the same time, higher coordination numbers with respect to the aristotype allow for accommodation of both bigger and smaller coordination environments, as was emphasized in Fig. 8.

The Ba- and Ba/Y-centred polyhedra interconnect along the *bc* plane forming a checkerboard-like pattern. There are 3 such layers in the structure of Ba_3_Y_2_[Si_2_O_7_]_2_ (Fig. 9c): the one at the origin of the *a* axis is formed by Ba1- and M2-centred polyhedra (A), followed by a more diverse layer of Ba3@O_14_, Ba4@O_12_, M1@O_15_, and M3@O_14_ (B), and the third layer formed by Ba2- and M4-centred polyhedra in the middle of the unit cell (C). These fragments stack on top of each other along the *a* axis, populating the unit cell of Ba_3_Y_2_[Si_2_O_7_]_2_ (divided by dashed line in Fig. 9a). The similar layers are observed in the substructure of orthorhombic Cs_3_CaBi[P_2_O_7_]_2_; however, it features alternation of a uniform layer formed by Cs-centred polyhedra and a chessboard-like arrangement of Cs-centred polyhedra and Bi-centred octahedra (Fig. 9d).

The heavy atoms in the structure of Ba_3_Y_2_[Si_2_O_7_]_2_ form a pattern that directly resembles the structure of zirconium silicide Zr_5_Si_4_.^64^ This resemblance follows the common trend for other oxide materials, *e.g.* silicates or aluminates, that are structurally related to Zintl phases.^65,66^ In these oxides, the Al or Si atoms adopt the structure patterns with the same connectivity as polyanionic networks in Zintl phases. However, we can draw even more connections between these two classes of materials which can provide a useful insight for the further material design. Many of the silicate structures retain the same ratio of heavy atoms to silica and crystallize in the space groups that are direct subgroups of the corresponding Zintl phases. This can be illustrated with examples of malvianovite Mn_5_Si_3_ (*P*6_3_/*mcm*)^67,68^ and apatite RE_5_[SiO_4_]_3_O (*P*6_3_/*m*).^69^ Similarly, silicide U_3_Si_2_ (*P*4/*mbm*)^70^ can be a Zintl aristotype for previously mentioned fresniote Ba_2_Ti[Si_2_O_7_]O (*P*4*bm*)^50^ or melilite Ca_2_Mg[Si_2_O_7_] (*P*4̄2_1_*m*).^71^ In these examples, bold text emphasizes the substructures related to corresponding Zintl phases.

**Fig. 10:**
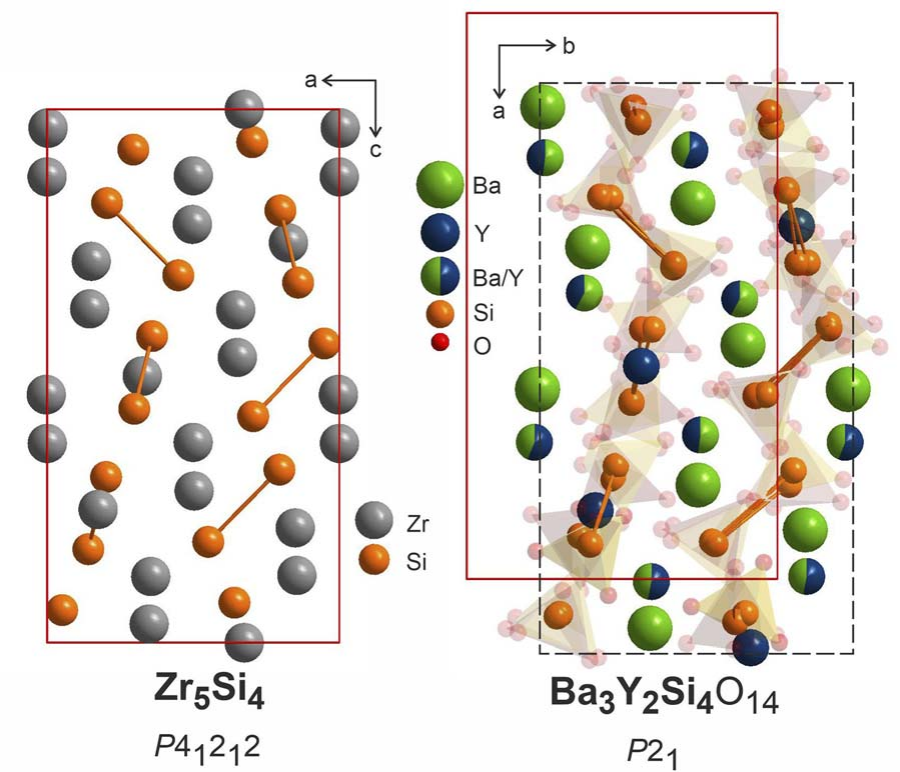
Structure of Zr_5_Si_4_ (*P*4_1_2_1_2),^64^ left, compared to Ba_3_Y_2_[Si_2_O_7_]_2_ refined in *P*2_1_, right. Disilicate groups are shown with partial transparency to emphasize the Ba–Y–Si network. The pairs of silicon atoms corresponding to disilicate groups are linked.

The structure of Zr_5_Si_4_ was solved in *P*4_1_2_1_2 space group which is enantiomeric with *P*4_3_2_1_2, the space group of the average model of phase B, and a direct supergroup for *P*2_1_. Fig. 10 shows the Zr_5_Si_4_ structure in comparison with the Ba–Y–Si network in the structure of Ba_3_Y_2_[Si_2_O_7_]_2_. The structure of the silicide features pairs of silicon atoms with a rather short Zr–Zr distance of 2.471 Å that resemble the distribution of disilicate groups in Ba_3_Y_2_[Si_2_O_7_]_2_. The ratio of heavy atoms to silicon also remains the same: 5Zr : 4Si and (3Ba + 2Y) : 4Si. The unit cell of a disilicate (9.04 × 9.04 × 16.47 Å^3^) has expanded by 100% in comparison to those of the zirconium silicide (7.12 × 7.12 × 13.0 Å^3^) to accommodate oxygen atoms.

An unknown amorphous content prevented further physical property investigation of phase B. We can assume that the range of applications for phase B could be very similar to phase A and other silicates. One might expect better ionic conductivity due to a high degree of disorder and respectively lower thermal conductivity (*vide infra*). At the same time, the lack of rigidity and amorphization after prolonged heat treatment makes it unsuitable for doping in the context of luminescence studies.^54^ Synthesis of this structure type in a chemical space where there is less competition with disorder will enable further exploration of these possibilities.

### 2.3. Thermal properties of Ba_5_Y_13_[SiO_4_]_8_O_8.5_

**Fig. 11:**
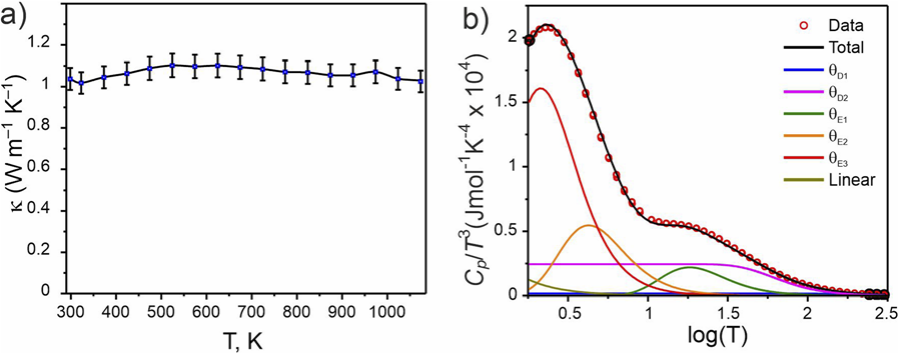
Physical properties of Ba_5_Y_13_[SiO_4_]_8_O_8.5_. (a) Thermal conductivity measured from 300 to 1073 K. (b) Heat capacity modelled as *C*_p_/*T*^3^(log *T*) through a combination of two Debye, three Einstein, and one linear term (Table S15†).

A pellet of Ba_5_Y_13_[SiO_4_]_8_O_8.5_ (phase A) was processed to >88% relative density (with respect to the theoretical density) *via* spark plasma sintering at 1843 K and 35 MPa uniaxial pressure (see Fig. S4†) which enabled measurement of thermal transport properties. The thermal conductivity of Ba_5_Y_13_[SiO_4_]_8_O_8.5_ was measured from 298 to 1073 K and has a low value of 1.04(5) W m^−1^ K^−1^ at room temperature (Fig. 11a). Temperature independence over this wide temperature range is observed, where *κ*_latt_ at 1073 K is 1.03(5) W m^−1^ K^−1^. This temperature independence is characteristic of glass-like phonon transport behaviour, in which a solid behaves like a disordered material, despite Ba_5_Y_13_[SiO_4_]_8_O_8.5_ being a crystalline material. Interestingly, these measured values are comparable directly to SiO_2_–Al_2_O_3_–Y_2_O_3_ glasses (≈0.8–1.0 W m^−1^ K^−1^ at room temperature).^72^ The thermal conductivity of Ba_5_Y_13_[SiO_4_]_8_O_8.5_ is lower than that of other silicates such as Y_2_SiO_5_ (≈1.6 W m^−1^ K^−1^),^73^ β-Y_2_Si_2_O_7_ (≈5.2 and 1.84 W m^−1^ K^−1^ at 300 K respectively 1473 K)^17,74^ and γ-Y_2_Si_2_O_7_ (≈5.1 and 1.9 W m^−1^ K^−1^ at 300 K respectively 1273 K) ceramics.^74^

The phonon-glass behaviour of Ba_5_Y_13_[SiO_4_]_8_O_8.5_ is further supported by the observation of excess heat capacity at low temperatures (Fig. 11b), which often arises from localised vibrations.^75^ The measured heat capacity of Ba_5_Y_13_[SiO_4_]_8_O_8.5_ is modelled with a combination of two Debye temperatures, three Einstein temperatures, and a linear contribution (Table S15†). The two Debye temperatures (*θ*_D_) of 910(10) and 300(10) K are necessary to fit the data in the high temperature region, while the three Einstein temperatures (*θ*_E_) of 90(5), 21(5), and 10.5(2) K are necessary to model the excess heat capacity observed at low temperatures. Models with fewer terms are not able to accurately reproduce the data (Fig. S28†).

The low *κ*_latt_, the glass-like temperature dependence, and the extracted Debye and Einstein temperatures can be related to the crystal structure of Ba_5_Y_13_[SiO_4_]_8_O_8.5_ through consideration of the structural motifs and bonding types present. The lower Debye temperature *θ*_D2_ of 300(10) K is comparable to that reported for Ba_2_SiO_4_ (332 K),^76^ and is associated with the A and B motifs within the structure of Ba_5_Y_13_[SiO_4_]_8_O_8.5_ (Fig. 4a) which consist of high atomic mass Ba with ionic, low-force-constant Ba–O bonds, and isolated SiO_4_ tetrahedra respectively. The higher frequency contributions of *θ*_D2_ (910(10) K) likely result from the condensed blocks of edge-sharing Y–O environments that resemble high-pressure high-temperature forms of Y_2_O_3_. This combination of multiple structural motifs with distinct bonding is likely responsible for the complex heat capacity observed at low temperature, for which the three Einstein temperatures are necessary. These represent non-propagating phonon modes, associated with localised oscillators of singular vibrational frequencies. The two low frequency terms of 21(5) and 10.5(2) K can be associated with the higher mass Ba species which occupy large coordination 8- and 9-fold coordination environments, and particularly the disordered distribution of Ba1 sites. The high frequency Einstein temperature of *θ*_E1_ = 90(5) K could arise from the lightweight isolated SiO_4_ tetrahedra which may act as localised oscillators. In particular, it is noted that the three oxygen sites coordinated to Si1 sites in the A motifs (O2, O5, and O6) exhibit the highest isotropic displacement parameters of all oxygen sites (Table S5†). The combination of multiple structural motifs within Ba_5_Y_13_[SiO_4_]_8_O_8.5_ with diverse atomic environments that distinct types of bonding culminate in increased phonon scattering to yield the low glass-like observed thermal conductivity.

According to recent reports on a correlation between structural rigidity and connectivity, a large Debye temperature can be a prerequisite for the material being a potential phosphor host.^32,54^ Since Ba_5_Y_13_[SiO_4_]_8_O_8.5_ is characterized by a band gap of 5.8(4) eV (see Fig. S27†), we can estimate a potential quantum yield of 80–90% (compared to experimentally measured materials with Ce^3+^ as the dopant) according to the correlation plot given by Brgoch *et al.*^32^ At the same time, one should be cautious as these are only the estimates and real phosphor efficiency can be proved only after successful experiments.

## 3. Conclusions

**Fig. 12:**
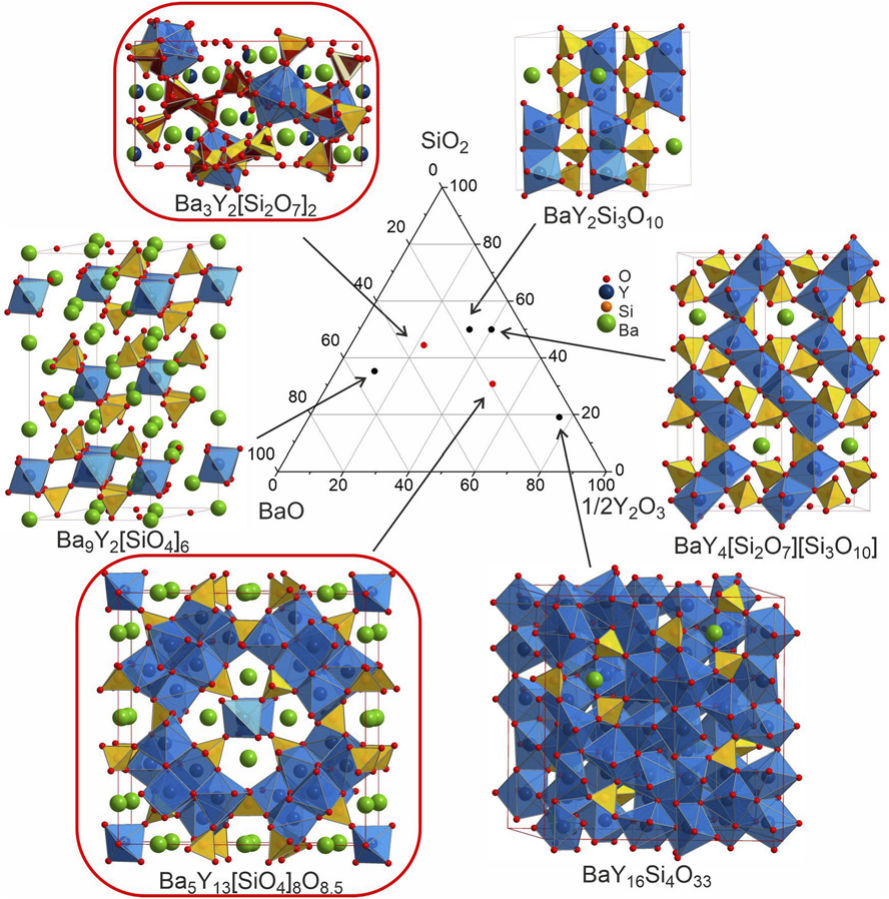
Comparison of the structures of the known Ba–Y-silicates emphasizing single yttrium-centred octahedra in Ba_9_Y_2_[SiO_4_]_6_ ^36^ and Ba_3_Y_2_[Si_2_O_7_]_2_ (phase B), dimers in BaY_2_Si_3_O_10_,^33^ chains of polyhedra in BaY_4_[Si_2_O_7_][Si_3_O_10_],^34^ condensed clusters in Ba_5_Y_13_[SiO_4_]_8_O_8.5_ (phase A), and a 3D network of polyhedra in BaY_16_Si_4_O_33_.^35^ Crystallographic details of these compounds are given in Table 1. Silica tetrahedra and yttrium-centred polyhedra drawn in yellow (yellow/red for phase B) and blue, respectively. Note that the coordination of yttrium atoms becomes more condensed with increasing yttrium content.

The discovery of new phases in a well-established phase field (Fig. 12) illustrates the challenges encountered in the exploration of chemical space that can affect both manual and automated experimental workflows. Because of the range of chemistries encountered in practice, an array of tools is needed to allow the deployment of workflows that match the specific chemistry under study, which can vary even within the same phase field.

This is illustrated by the exploration of BaO–Y_2_O_3_–RuO_2_–SiO_2_ chemical space. Ba_5_Y_13_[SiO_4_]_8_O_8.5_ (phase A) was located and isolated through an iterative route that combined electron, X-ray, and neutron diffraction with chemical analysis methods. Ba_3_Y_2_[Si_2_O_7_]_2_ (phase B) required in addition the introduction of a quantitative probabilistic composition prediction tool that explicitly dealt with uncertainties in the available phase assemblage information to navigate around the glass field competing with the crystalline target material. The complementary use of these exploration routes enabled the isolation of two phases in the absence of single crystals, and, by minimizing the impact of the glass-forming region, allowed investigation of a wide compositional range.

Each phase corresponds to a new structure type that reveals unique structural motifs and different degrees of disorder. Phase A is a mixed silicate-oxide where silica-coordinated motifs are embedded within interconnected columns of yttrium-centred polyhedra that are characteristic of the high-pressure and high-temperature modifications of Y_2_O_3_. These unusual rare earth environments are located in a scaffold whose optical and thermal properties indicate promise as a host for activator ions to create phosphors for solid-state lighting. As a direct superstructure of Cs_3_CaBi[P_2_O_7_]_2_,^58^ phase B features more conventional structural units but illustrates how those units can be assembled in a distinctive partially ordered manner on that extended framework, exploiting flexible silicate disorder to create appropriate coordination environments for the disordered cations. The persisting disorder suggests the prospect of chemistry leading to more completely ordered structures from targeted exploration of substitutional chemistry. Furthermore, a structural relationship between the cation arrangement in phase B and the Zintl phase Zr_5_Si_4_ ^64^ aristotype reveals an unexpected perspective for the design of other silicate materials. Such opportunities illustrate the enabling nature of the discovery of new structure types, as they allow subsequent targeted modification, and the importance of developing the appropriate workflows to access them. These workflows can exploit multiple source diffraction combined with automated identification of composition regions affording the new structures, accounting for experimental uncertainty, to reduce the barriers to the discovery (*i.e.* the experimental realisation) of new structures in the poorly explored high-dimensional chemical spaces that are likely locations for such materials.

While RuO_2_ from the initial BaO–Y_2_O_3_–RuO_2_–SiO_2_ system was not incorporated into the new structures and formed the known by-products instead, we cannot discard the possibility that it will form any quinary phases as the chemical space has not been explored in its entirety yet. Ruthenium(iv,v) oxide compounds typically favour octahedral coordination with the average Ru–O bond around 2.0 Å.^27^ Theoretically, ruthenium could coexist with a slightly larger yttrium atoms which coordinates octahedrally in the structure of phase A, but instances of Y/Ru mixing in oxides are extremely rare (*e.g.*, high-pressure cubic perovskite BaY_0.33_Ru_0.67_O_3_)^31^ as these atomic species usually separate onto distinct cation sites. Differences in coordination environments between ruthenium and silicon may limit structural formation; however, we can draw optimism from minerals that are known to host SiO_4_ tetrahedra combined with differently coordinated transition metals surrounding heavy atoms. Some of the examples are gillespite BaFeSi_4_O_10_ ^77^ that contains square coordinated Fe atoms; or the synthetic gadolinite, NiYb_2_Be_2_Si_2_O_10_ ^78^ with octahedrally-coordinated nickel. Therefore, search of the quinary compounds in this system is not exhausted and can become a target for further exploration based on the experimental knowledge and array of tools presented in the current study.

## Author contributions

NLG, LMD, JBC, and MJR conceptualized the project. NLG developed synthetic methodology and utilized it for experimental investigation. DR and MSD developed computational tool PICIP and processed corresponding data. MZ, CMR, and BM collected CRED data and developed corresponding structure models. NLG and LMD conducted PXRD analysis of the samples. MAW and CMC aided structure solution of the phase B from PXRD data. NLG developed crystallographic description of both phases with aid from LMD and JBC. MZ, MS and JAN performed EDX analysis. CJH measured and interpreted UV/vis data. JW collected and processed IR spectra. NLG, JAN, HN and LMD studied thermal properties. MJR directed the project and acquired funding. NLG composed the initial draft of the manuscript. All authors contributed to discussing, editing, and reviewing the manuscript.

## Conflicts of interest

The authors declare no competing interests.

## Supplementary Material

SC-015-D4SC04440A-s001

SC-015-D4SC04440A-s002

## Data Availability

The data that support the findings of this study are openly available in the University of Liverpool Research Data Catalogue at https://datacat.liverpool.ac.uk/id/eprint/2738.
