# Supplementary material for: Navigation through high-dimensional chemical space: discovery of Ba5Y13[SiO4]8O8.5 and Ba3Y2[Si2O7]2
Source: Chem Sci. 2024 Sep 12;15(40):16503–18. doi: 10.1039/d4sc04440a (PMC11413733; doi:10.1039/d4sc04440a)
Supplement: SC-015-D4SC04440A-s001 [file SC-015-D4SC04440A-s001.pdf]

Supplementary Information (SI) for Chemical Science.
This journal is © The Royal Society of Chemistry 2024

Supporting Information

Navigation through High-dimensional Chemical Space: Discovery of $Ba_5Y_{13}[SiO_4]_8O_{8.5}$ and $Ba_3Y_2[Si_2O_7]_2$

Authors

Nataliya L. Gulay[1], Marco Zanella[1], Craig M. Robertson[1], Daniel Ritchie[1,2], Manel Sonni[1], Matthew A. Wright[1], Jon A. Newnham[1], Cara J. Hawkins[1], Jayne Whitworth[1], Bhupendra Mali[1], Hongjun Niu[1], Matthew S. Dyer[1], Christopher M. Collins[1,2], Luke M. Daniels[1], John B. Claridge[1], Matthew J. Rosseinsky[1,2]*

1. Department of Chemistry, University of Liverpool, Materials Innovation Factory, 51 Oxford Street, Liverpool, L7 3NY, UK

2. Leverhulme Research Centre for Functional Materials Design, Materials Innovation Factory, 51 Oxford Street, University of Liverpool, Liverpool L7 3NY, UK.

Supplementary materials

Contents:

SI1.	Methods	2
SI2.	Exploration of the Ba-Y-Ru-Si-O chemical space: details	7
SI3.	CRED analysis of phase A	12
SI4.	Rietveld refinement of phase A	17
SI5.	CRED analysis of phase B	24
SI6.	Rietveld refinement of Phase B	26
SI7.	Additional crystallographic information on Phase B	35
SI8.	Additional measurements	40
SI9.	References	43

SI1. Methods

1.1 Exploration of the Ba-Y-Si-Ru-O chemical space

The samples were prepared using high-purity starting materials: barium carbonate (Alfa Aesar, 99%), yttrium (III) oxide (Alfa Aesar, 99.999%), ruthenium (IV) oxide (Sigma Aldrich, 99.9%), and silicon dioxide nanopowder (Sigma Aldrich, 99.5%). The oxides were kept in a drying furnace (220°C) prior to synthesis, and barium carbonate was additionally heated to 600°C overnight to ensure accurate stoichiometry.

For the exploration, the first four samples were evenly spaced within the $BaRuO_3$-$Y_2Ru_2O_7$-$Y_2Si_2O_7$ phase field (marked with blue in Figure SI1). The dried oxides were weighed to match the stoichiometry of the sample to the total weight of ~1 g. The mixtures were homogenized using an agate mortar and pestle, placed in alumina crucibles and calcined in air at 1273 K over 12 hours (for all described steps, heating and cooling rate was kept at 5 K/min). After that, the powders were extracted from the crucibles, ground in a mortar and used to make two pellets per sample, which were consequently heated to 1473 and 1573 K and kept at these temperatures for 24 h. After reaction, the pellets were ground to fine powder and studied by means of powder X-ray diffraction (PXRD). The samples reacted at 1473 K consisted of known phases, but two of the samples heated to 1573 K showed some unidentified peaks (Phase A), which could not be matched with any known compounds. The particles of these sample were studied via transmission electron microscopy (TEM) with energy dispersive X-ray (EDX) analysis, which revealed compositions different to those corresponding to known compounds formed by these elements (Figure SI3). The averaged values of the clustered measured compositions were used for the preparation of next samples using the same synthesis technique (1573 K as reaction temperature). This next generation of samples revealed even higher content of the unidentified phase. EDX analysis and consequent synthesis of new samples was repeated one more time until a significant yield of Phase A was obtained (Sample 10 in Table SI1).

At the same time, the same compositions with unidentified reflections from the first batch were analysed using the Probabilistic Isolation of Crystalline Inorganic Phases (PICIP) tool. The ratio between known phases was estimated using Rietveld analysis, and the residual composition was selected as the next target for the synthesis. Surprisingly, these compositions away from the previously explored ones corresponding to phase A, as marked by arrows at Figure SI1, a. The samples were studied using the same synthetic approach as before and analysed using PXRD. In some of the samples, another distinct set of unidentified reflections was present (denoted as Phase B). The EDX analysis of these samples did not produce any reliable cluster of compositions (see Figure SI5), which is probably caused by a partial glassification of the sample. Furthermore, these samples had melted during the repeated heating at 1573 K as an attempt of homogenization. Therefore, PICIP analysis was used two more times to guide the exploration of this system, and the synthesis temperature was adjusted to 2 hours to prevent the formation of an amorphous phase. After that, a sample with a higher yield of the second crystalline phase B was obtained (Figure SI2, b). This sample was succesfully studied by means of CRED (details given further in text) and all particles that were reflecting according to the same symmetry were analysed by EDX. The final composition was adjusted along with heating time to obtain the highest yield of the crystalline phase B (Sample 18 in Figure SI2, b).

Both phase A ($Ba_5Y_{13}[SiO_4]_8O_{8.5}$) and phase B ($Ba_3Y_2[Si_2O_7]_2$) form white powders, which are stable in air under ambient conditions. The complete list of studied compositions is given in Table SI1, and

powder patterns of some representative samples are shown in Figure SI2, a and b, to illustrate the discovery and isolation of both phases.

1.2. Probabilistic Isolation of Cristalline Inorganic Phases (PICIP)

PICIP is a computational tool which directs iterative experimental sampling of compositional space with the aim of isolating an unknown crystalline phase that has been detected by PXRD. Of the known crystalline phases identified in a PXRD pattern, PICIP uses the estimated relative weight fractions of those phases obtained from quantitative Rietveld analysis to calculate the average composition of the known phases, taking into account experimental uncertainty. An example of this input data is given in Table SI2. Assuming that just one unknown phase is detected, the estimated relative weight fractions were exact and covered all other phases, and the reaction was a closed thermodynamic system, then the unknown crystalline phase would be somewhere on the line extending from the sampled composition away from the average composition of known phases. In practice, estimated relative weight fractions will have an associated uncertainty and so this line is treated as an estimated direction rather than the ground truth. PICIP accounts for uncertainty by computing a cone shaped probability density (Figure 6, b, Figure SI5). This probability cone points in the estimated direction, with an aperture proportional to the expected degree of experimental error and an apex at the sampled composition. The advantage of this probabilistic treatment is that when multiple sample compositions are found to contain the unknown phase, the information from them can easily be combined by multiplying the probability densities.

In the case that multiple unknown phases are present in a sample, PICIP would instead direct a search towards the weighted average composition of those unknown phases, with the weighting equal to their relative amounts. This average composition will change from sample to sample as the relative amounts of unknown phases will change. The probabilistic modelling approach manages inherent uncertainties and variations in the target's position. By aggregating the probability densities from multiple samples, the resultant probability density predicts the average relative weights of the unknown samples effectively accommodating the shifting position of the average composition of unknown phases.

Since any amorphous phases present will not contribute to the weighted fraction of known crystalline phases in the sample, their composition will also contribute to the weighted average composition of unknown phases in the sample. As a result, compositions suggested by PICIP will not attempt to reduce the amount of amorphous phase that forms, but will still increase the yield of unknown crystalline phases relative to competing known crystalline phases.

1.3. Synthesis of phase A – $Ba_5Y_{13}[SiO_4]_8O_{8.5}$.

For the preparation of the phase A, the dried precursors were weighted to achieve 0.5 g of the final material in a 5 : 13 : 8 ratio of $BaCO_3$: $YO_{1.5}$: SiO_2 that corresponds to the refined formula $Ba_5Y_{13}[SiO_4]_8O_{8.5}$. The mixtures were calcined at 1273 K as described above, ground and used for the next stage of the synthesis. $Ba_5Y_{13}[SiO_4]_8O_{8.5}$ (phase A) was prepared by pressing calcined powder in a 10mm pellet and annealing it in an alumina crucible in air at 1573 K for 24 h. After cooling down, the pellet was crushed and ground to a fine powder, which was later studied by X-ray diffraction to monitor the phase purity. This step was repeated 2 more times to ensure complete reaction of the precursors. For neutron diffraction studies, powders from multiple batches were combined together to a total weight of ~5g, mixed with a mortar and pestle and homogenized by heating them to 1573 K for 12 h.

1.4. Synthesis of phase B – $Ba_3Y_2[Si_2O_7]_2$

Nearly phase-pure samples with respect to the crystalline phase B (according to powder diffraction) were prepared from dried precursors weighted in a 31 : 23 : 46 ratio of $BaCO_3$: $YO_{1.5}$: SiO_2 to obtain 0.5 g of final material. The calcination was performed using the same routine as for the phase A. The calcined powder was pressed in a pellet, placed in an alumina crucible and annealed for 12 hours at 1573 K. After cooling down, the pellet was carefully removed from the crucible and ground to a fine white powder, which was routinely checked by PXRD. For the neutron diffraction investigation, 10 individual powder samples were mixed together and annealed at 1473 K for 24 h to ensure homogeneity. The powder was carefully scraped from the crucible and ground again. In total, around 5 g of the sample was prepared.

1.5. Preparation of dense pellets of Phase A.

Dense pellets for property measurements were obtained using spark plasma sintering (SPS). A carbide die (inner diameter of 10 mm) was lined with a carbon foil and filled with ~0.5 g of $Ba_5Y_{13}[SiO_4]_8O_{8.5}$. The die was placed in a chamber of the Thermal Technology LLC DCS10 furnace, which was evacuated to a 10^{-3} mbar vacuum before sintering. The temperature was monitored through a bore-hole in the side of the die set via a pyrometer. The uniaxial pressure applied to the pellet was kept stable at 35 MPa, and the temperature was rapidly increased to 1773 K (100 K/min) followed by slower heating to 1843 K (30 K/min) which is about 20 K below the melting point (~1863 K, see Figure SI4). The sample was kept at this temperature for 2 minutes and cooled down to room temperature. After that, the pellet was taken out of the die and the carbon foil was removed mechanically by lightly polishing with SiC polishing paper. The leftover carbon was burned out at 1573 K (12 hours) in air in a muffle furnace. This treatment resulted in dense pellets of >88% of crystallographic density with respect to the theoretical density and the content of the main phase.

1.6. Powder diffraction

Routine analysis of the polycrystalline samples was performed by measuring X-ray diffraction data on a PANalytical X'Pert diffractometer (monochromatic Co $K\alpha_1$ radiation, λ=1.78896 Å). For this, a small amount of sample was evenly distributed over a greased glass slide and measured on diffractometer in reflection mode. Initial phase identification was made by comparison of the diffraction data with the patterns of known compounds using X'Pert HighScore Plus software.

For the collection of high-resolution data, the fine powders were loaded into borosilicate glass capillaries of 0.3 mm diameter. Synchrotron diffraction data were collected at Diamond Light Source, beamline I11, using Multianalyzer crystal detector (MAC) and Mythen position sensitive detector (PSD) for high resolution room temperature and low temperature measurements. For the latter, the capillary was cooled down to 100 K by Oxford Cryostream Plus.

For collection of time-of-flight (TOF) neutron diffraction data, ~5 g of the polycrystalline samples were loaded in an 8 mm diameter vanadium can and studied on the Polaris (Phase A) and GEM (Phase B) instruments at ISIS neutron source. The measurements were done at room temperature. The data was corrected with respect to absorption and further structure refinement was done using TOPAS Academic V7.[1] The bond valence sum (BVS) was determined using GBondStr tool of FullProf suite.[2]

Crystal structures were visualized using Diamond software (version 4.6.8.) [3]

1.7. Continuous Rotation Electron Diffraction (CRED)

The samples for CRED analysis were prepared dispersing a small amount of well-ground powder in 4 ml of ethanol and leaving the suspension decanting for about 1 h to allow the largest agglomerates to precipitate. An aliquot of the supernatant was deposited on a lacey carbon film coated TEM grid and let dry in air.

CRED data were collected, at room temperature, on single particles on a 200KV JEOL 2100+ equipped with a GATAN Rio Camera using insteaDMatic software.[4] Data were collcted using a tomography TEM holeder which allows to collect data in the ±70 degrees range. CRED data were initially processed using REDp software[5,6] and then further reduced using micro ED CrysAlisPro v43 from Rigaku. Merging and scaling of multiple datasets was performed using XPREP software from Bruker[7]. Space group and initial crystal structure were derived using ShelXT.[8] The found space group was confirmed through the study of the diffraction systematic absences. In the case of phase B, the presence or absence of systematic absences was confirmed analysing the intensity of each pixel on the relevant CRED frames. Final crystal structure was derived using Olex2 from OlexSys.[9]

Energy dispersive X-ray spectroscopy (EDX) data were measured on the particles to confirm their composition before collecting CRED data.

1.8. Energy dispersive X-ray spectroscopy (EDX)

For both bulk samples and the particles analysed by CRED, the compositions were determined using energy dispersive X-ray spectroscopy (EDX) on a JEOL2100+ operating at 200 kV equipped with SDD detector from Oxford Instruments (Model: X-Max 65T with a 65 mm^2 surface area detection). Samples were dispersed on carbon coated copper TEM grids and were inserted using a tomography holder. In order to reduce the signal coming from the holder the sample was tilted 10 degrees toward the detector. Data acquisition and analysis were performed using the Aztec software. Correction factors were determined measuring corresponding standards for each chemical element. Small isolated particles were picked for EDX analysis to avoid collecting data from agglomerates.

1.9. Maximum entropy method (MEM)

Electron density distributions were calculated from I11 synchrotron powder X-ray diffraction data using the maximum entropy method (MEM). The observed structure factor was extracted from data using a Rietveld refinement performed in Jana2006.[10] The refinement models were taken from Topas refinements and atomic positions were kept fixed; as were isotropic displacements of oxygen sites. Peak profiles were fit empirically using a pseudo-Voigt function and the Stephens model for anisotropic strain broadening in the monoclinic setting, as was done in Topas. The background was fit manually using a 60 point linear interpolation to ensure that peak intensities were extracted to the highest degree of accuracy. The electron densities were generated with MEM using the Sakata-Sato algorithm in BayMEM[11] on a grid of 300×300×300 voxels and run till the combined FG constraint met its convergence criteria of 1.0. Electron densities were visualized using VESTA.[12]

1.10. IR-spectroscopy

The infra-red spectra were recorded at Bruker Vertex V70 FT-IR spectrometer using diamond ATR accessory. 16 scans were made both for the background and the sample, and the replicated datasets were

averaged at the end. The spectra were compared with reported ones in the regions for the structural groups of interest (i.e. OH-groups).

1.11. Heat capacity and thermal conductivity measurements

Thermal diffusivity data (α) were measured through the laser flash method using a Netzsch LFA 457. A pellet (approximately 10 mm in diameter and approx. 0.8 mm thick) was coated in colloidal graphitic carbon before being placed inside the sample chamber, which was evacuated and purged three times with helium before measurement. Measurements were made under a flow (100 mL min^{-1}) of helium. Data were collected in 50 K steps in the temperature range 298 to 1073 K using a heating rate of 5 K min^{-1} and three-minute equilibration at each temperature before measurement. Thermal diffusivities were obtained by fitting a Cowan model to the raw data. Three measurements were performed at each temperature and their diffusivities were averaged, with the standard deviation of points ≤0.6 %. Sufficient time (120 seconds) separated each of the three measurements allowing for equilibration for local heating of the sample from the laser.

Heat capacities of the sample in a range from 1.8-300 K were measured using the heat capacity option on the Quantum Design Physical Properties Measurement System (PPMS) using the relaxation method. Above room temperature, heat flux profiles were measured using a Netzsch DSC 404 F1 differential scanning calorimeter under a 50 mL min^{-1} flow of nitrogen. Continuous data were recorded from 300 to 1073 K using a heating rate of 10 K min^{-1}. Data were measured from a sapphire standard of similar mass (37 mg) under identical conditions to determine the heat capacity (C_P) of the sample. Errors on the heat capacity are assumed to be 5% as advised by the manufacturer. The thermal conductivity (κ) was calculated by combining the diffusivity and heat capacity data through $\kappa(T) = \alpha(T)\cdot C_P(T)\cdot\rho$, assuming a constant pellet density ρ.

The heat capacity data was modelled in a range from 1.8-300 K using a linear combination of Debye and Einstein terms. The modelling was made using the Heat Capacity tool of the Liverpool Materials Discovery Server (LMDS).[13]

1.12. UV-Vis spectrometry

Diffuse reflectance of Phase A powder was measured using an Agilent Cary 5000 between 200 and 2500 nm with a step size of 1 nm. Calibration to 100% and 0% reflectance was performed prior to measurement using a PTFE standard and a light trap, respectively. The band gap was determined from a Tauc plot using a method described by Makuła et al.[14]

SI2. Exploration of the Ba-Y-Ru-Si-O chemical space: details.

Table SI1. Studied compositions in the Ba-Y-Ru-Si-O chemical space that led to discovery of the new compounds $Ba_5Y_{13}[SiO_4]_8O_{8.75}$ (phase A) and $Ba_3Y_2[Si_2O_7]_2$ (phase B). Phase analysis is given for samples reacted at 1573 K based on in-house data.

	Composition	Identified phases
1	$Ba_{9.2}Y_{40.8}Ru_{41.4}Si_{8.6}O_{170.4}$	$Y_2Ru_2O_7$ + $Ba_9Y_2[SiO_4]_6$ + phase A
2	$Ba_{17.2}Y_{32.8}Ru_{33.1}Si_{16.9}O_{166.4}$	$Y_2Ru_2O_7$ +$Ba_9Y_2[SiO_4]_6$ + phase A
3	$Ba_{33.9}Y_{16.1}Ru_{41.5}Si_{8.5}O_{158.1}$	$BaRuO_3$ + $Y_2Ru_2O_7$ + Ba_2SiO_4 + $Ba_9Y_2[SiO_4]_6$
4	$Ba_9Y_{41}Ru_{16.5}Si_{33.5}O_{170.5}$	$BaY_2Si_3O_{10}$ + $Y_2Ru_2O_7$ + Y_2SiO_5
5	$Ba_{4.7}Y_{16.1}Ru_{9.7}Si_7O_{62.25}$	$Y_2Ru_2O_7$ + $Ba_9Y_2[SiO_4]_6$ + Y_2O_3 +phase A
6	$Ba_{6.7}Y_{16.5}Ru_{5.4}Si_{9.3}O_{60.85}$	$Y_2Ru_2O_7$ + $Ba_9Y_2[SiO_4]_6$ + phase A
7	$Ba_{12}Y_{52}Ru_{14}Si_{23}O_{164}$	phase A + Y_2O_3 + $Y_2Ru_2O_7$ + Y_2SiO_5
8	$Ba_{16}Y_{50}Ru_4Si_{29}O_{157}$	phase A + $Y_2Ru_2O_7$ + Y_2SiO_5 + $Ba_9Y_2[SiO_4]_6$ + Y_2O_3
9	$Ba_9Y_{51}Ru_{21}Si_{19}O_{165.5}$	phase A + Y_2O_3 + $Y_2Ru_2O_7$ + Y_2SiO_5
10	$Ba_{10}Y_{26}Si_{16}O_{81}$	phase A + Y_2O_3
11	$Ba_{12}Y_5Ru_{10}Si_{11}O_{62}$	Phase B + $Y_2Ru_2O_7$ + $Ba_9Y_2[SiO_4]_6$ + $BaRuO_3$
12	$Ba_{14}Y_7Ru_6Si_{13}O_{61}$	$BaRuO_3$ + $Y_2Ru_2O_7$ + $Ba_9Y_2[SiO_4]_6$ + phase B
13	$Ba_{15}Y_5Ru_2Si_{18}O_{61}$	phase B + $BaSiO_3$ + $BaRuO_3$ + $Ba_9Y_2[SiO_4]_6$
14	$Ba_{14}Y_1Ru_8Si_{15}O_{62}$	$BaSiO_3$ + $BaRuO_3$ + $Y_2Ru_2O_7$+ phase B
15	$Ba_{36}Y_{18}Ru_8Si_{38}O_{155}$	phase B + $Y_2Ru_2O_7$ + $Ba_9Y_2[SiO_4]_6$ + $BaRuO_3$
16	$Ba_{33}Y_{24}Ru_1Si_{42}O_{155}$	phase B + $Ba_9Y_2[SiO_4]_6$
17	$Ba_{43}Y_{16}Ru_1Si_{41}O_{151}$	phase B + $Y_2Ru_2O_7$ + $Ba_9Y_2[SiO_4]_6$
18	$Ba_{14}Y_5Si_{19}O_{61}$	phase B + $Ba_9Y_2[SiO_4]_6$ + $BaSiO_3$
19	$Ba_{31}Y_{23}Si_{46}O_{157.5}$	Phase B + $BaSiO_3$

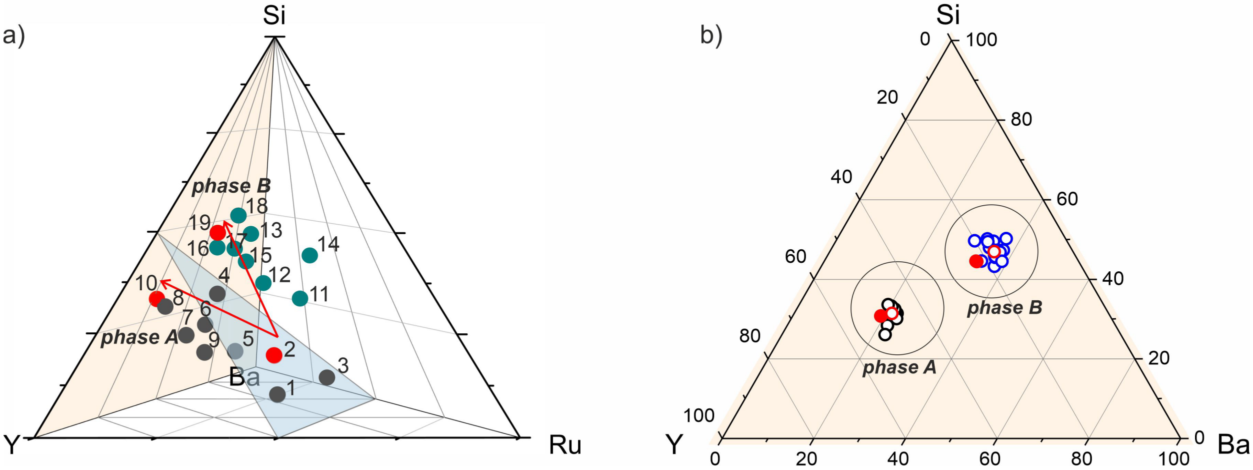

Figure SI1. (a) Quaternary BaO-$YO_{1.5}$-RuO_2-SiO_2 diagram with the compositions of the studied samples corresponding to phases A and B marked in grey and light-sea green respectively. Blue shading emphasized a starting phase field of $BaRuO_3$-$Y_2Ru_2O_7$-$Y_2Si_2O_7$ where the first four samples were probed. The sample numbers correspond to those in Table SI1. (b) Results of EDX analysis of the particles studied by CRED (black and blue open circles for Samples 10 and 18, respectively), their average (red open circles), and refined compositions (red filled circles) of phase A ($Ba_5Y_{13}[SiO_4]_8O_{8.5}$) and phase B ($Ba_3Y_2[Si_2O_7]_2$) based on Rietveld analysis.

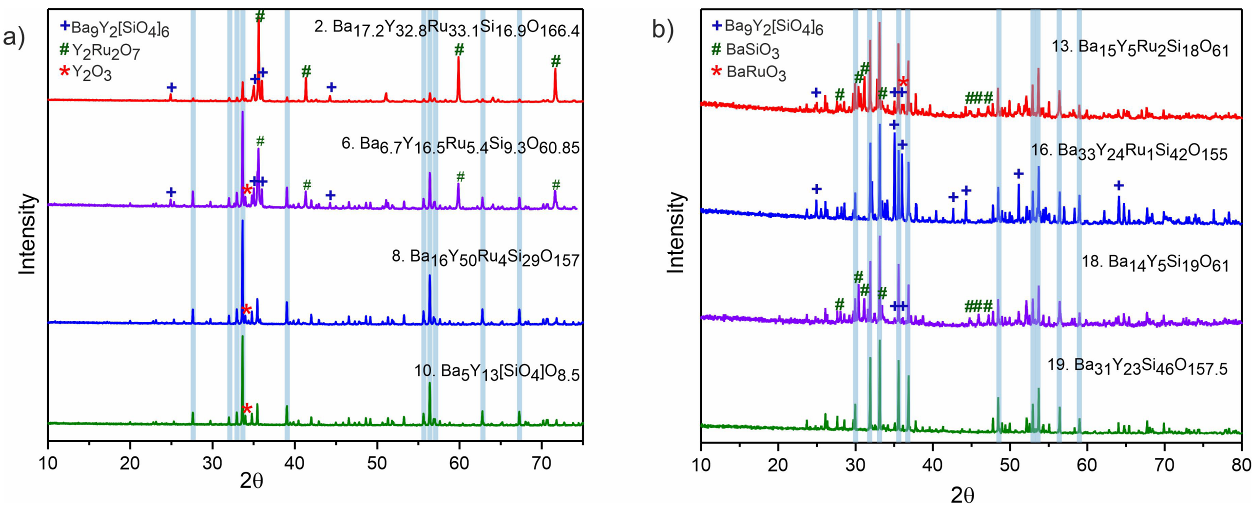

Figure SI2. (a) PXRD patterns (Co Kα radiation) for the selected samples in the Ba-Y-Si-Ru-O chemical space showing the tuning of composition which led to almost phase-pure phase A ($Ba_5Y_{13}[SiO_4]_8O_{8.5}$) Sample 10 ($Ba_{10}Y_{26}Si_{16}O_{81}$). The strongest reflections corresponding to phase A are highlighted in blue. (b) PXRD patterns (Co Kα radiation) for the selected samples in the Ba-Y-Si-Ru-O chemical space showing the tuning of composition which lead to almost phase-pure phase B ($Ba_3Y_2[Si_2O_7]_2$) Sample 19 ($Ba_{31}Y_{23}Si_{46}O_{157.5}$). The strongest reflections corresponding to phase B are highlighted in blue.

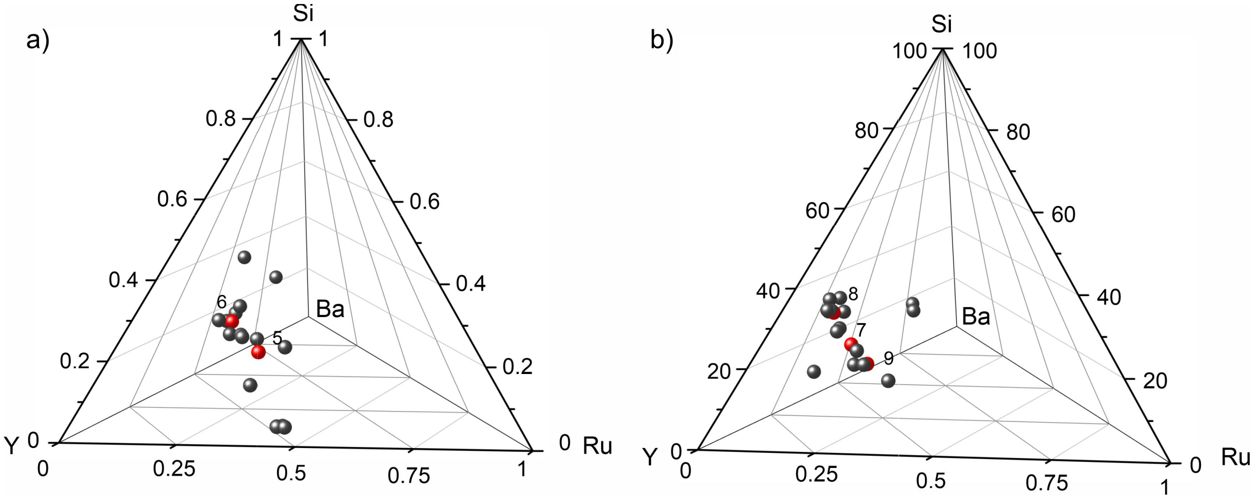

Figure SI3. **Isolation of phase A:** EDX analysis of the particles from the samples 2 ($Ba_{17.2}Y_{32.8}Ru_{33.1}Si_{16.9}O_{166.4}$, a) and 6 ($Ba_{6.7}Y_{16.5}Ru_{5.4}Si_{9.3}O_{60.85}$, b). Grey spheres mark measured compositions while red correspond to averaged values for the clusters which were used in the next stage of the phase exploration.

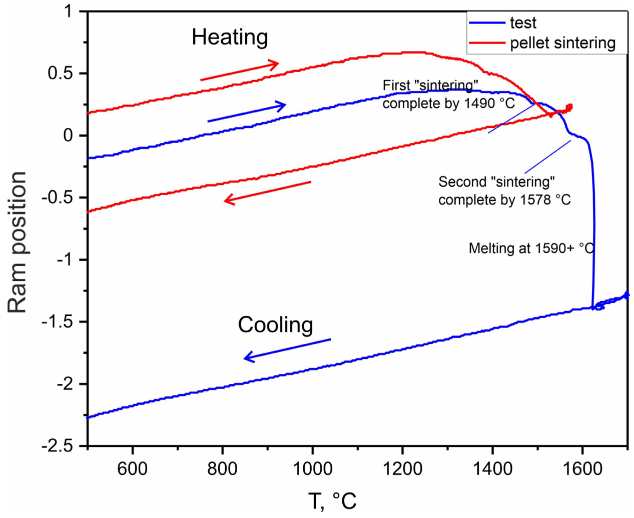

Figure SI4. Spark plasma sintering of the pellets of phase A. The sample melts above 1863 K.

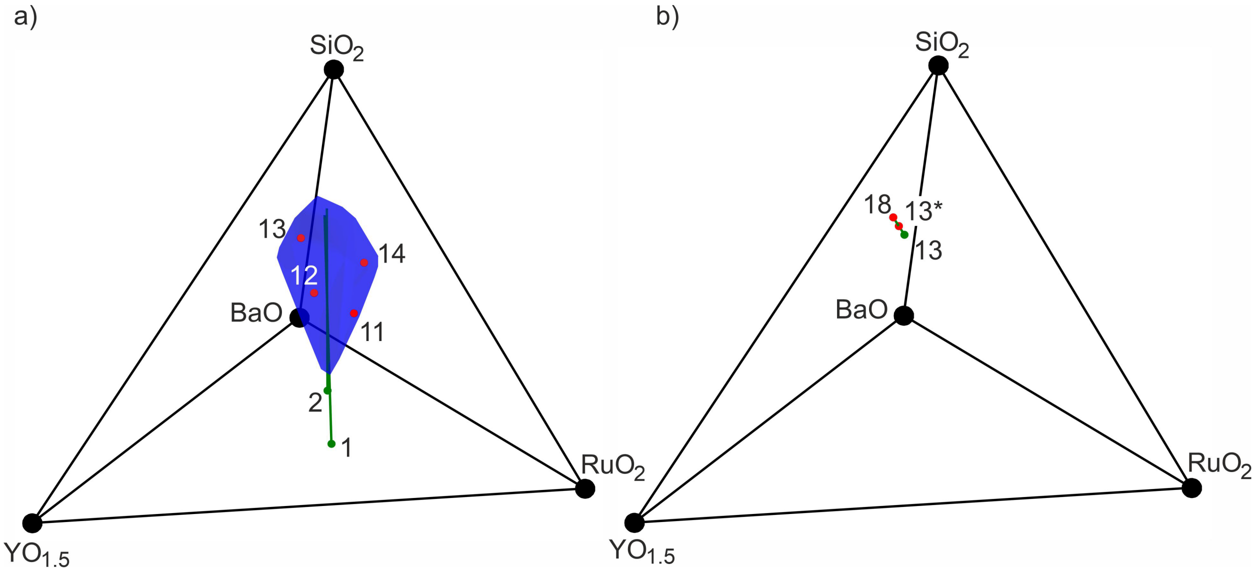

Figure SI5. **PICIP directed isolation of phase B:** (a) Quantitative phase analysis original synthesis of samples 1 and 2 (green dots, Table SI2) was used as input to PICIP generating a probability density for the composition of unknown phases (blue contour at 50 % probability). The compositions of samples 11-14 (red dots, Table SI1) were chosen to sample the region of high probability density. (b) Sample 13 (green dot) showed increased content of the unknown Phase B, and quantitative phase analysis of this sample (Table SI2) was used by PICIP to generate a new probability density. Samples 13* ($Ba_{15}Y_5RuSi_{18}O_{61}$) and 18 (red dots) were chosen along the line of highest probability density. Sample 18 contained sufficiently high content of Phase B to allow for optimisation of Phase B content yielding sample 19.

Table SI2. Results of quantitative phase analysis of selected compositions in the Ba-Y-Ru-Si-O chemical space that led to discovery of a new compounds $Ba_3Y_2[Si_2O_7]_2$ (phase B). Phase analysis is given for samples reacted at 1573 K based on in-house data with respect to known phases. The data was used by PICIP to generate the next compositions to study. After two iterations of PICIP, sample 18 was obtained.

Sample number	Composition	Identified phases (wt%)
1	$Ba_{9.2}Y_{40.8}Ru_{41.4}Si_{8.6}O_{170.4}$	97(1)% $Y_2Ru_2O_7$ + 3(1)% $Ba_9Y_2[SiO_4]_6$
2	$Ba_{17.2}Y_{32.8}Ru_{33.1}Si_{16.9}O_{166.4}$	70(1)% $Y_2Ru_2O_7$ +30(1)% $Ba_9Y_2[SiO_4]_6$
13	$Ba_{15}Y_5Ru_2Si_{18}O_{61}$	76(1)% $BaSiO_3$+ 17(1)%$Ba_9Y_2[SiO_4]_6$ + 7(1)% $BaRuO_3$
18	$Ba_{14}Y_5Si_{19}O_{61}$	phase B + $Ba_9Y_2[SiO_4]_6$ + $BaSiO_3$

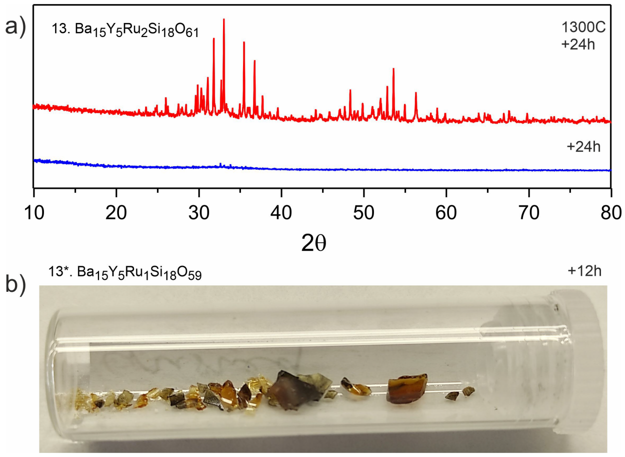

Figure SI6. (a) PXRD of the $Ba_{15}Y_5Ru_2Si_{18}O_{61}$ sample 13 after one and two stages of re-annealing. Note that the sample becomes completely amorphous. (b) A photo of the inhomogeneous melted chunks of another batch with a composition of $Ba_{15}Y_5Ru_1Si_{18}O_{59}$. The sample melted after 12h at 1573 K.

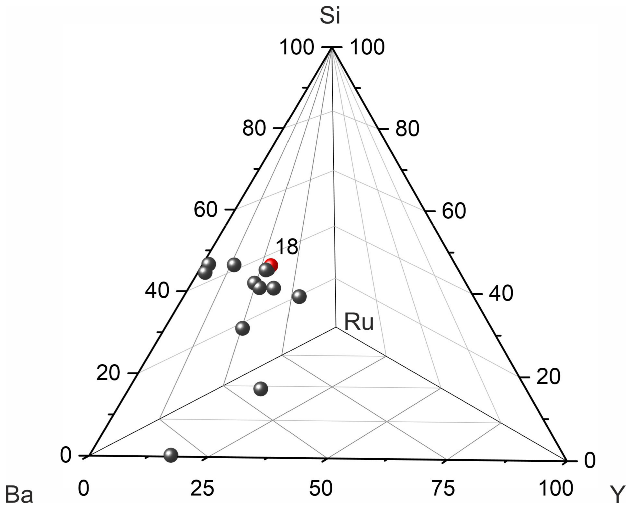

Figure SI7. **Isolation of phase B:** EDX analysis of the particles from the sample 13, $Ba_{15}Y_5Ru_2Si_{18}O_{61}$. Grey spheres mark measured compositions while red corresponds to the composition of the sample (see Table SI1). Note that the points are broadly distributed and the compositions lay close to the starting point preventing advancement of the exploration.

SI3. CRED analysis of phase A

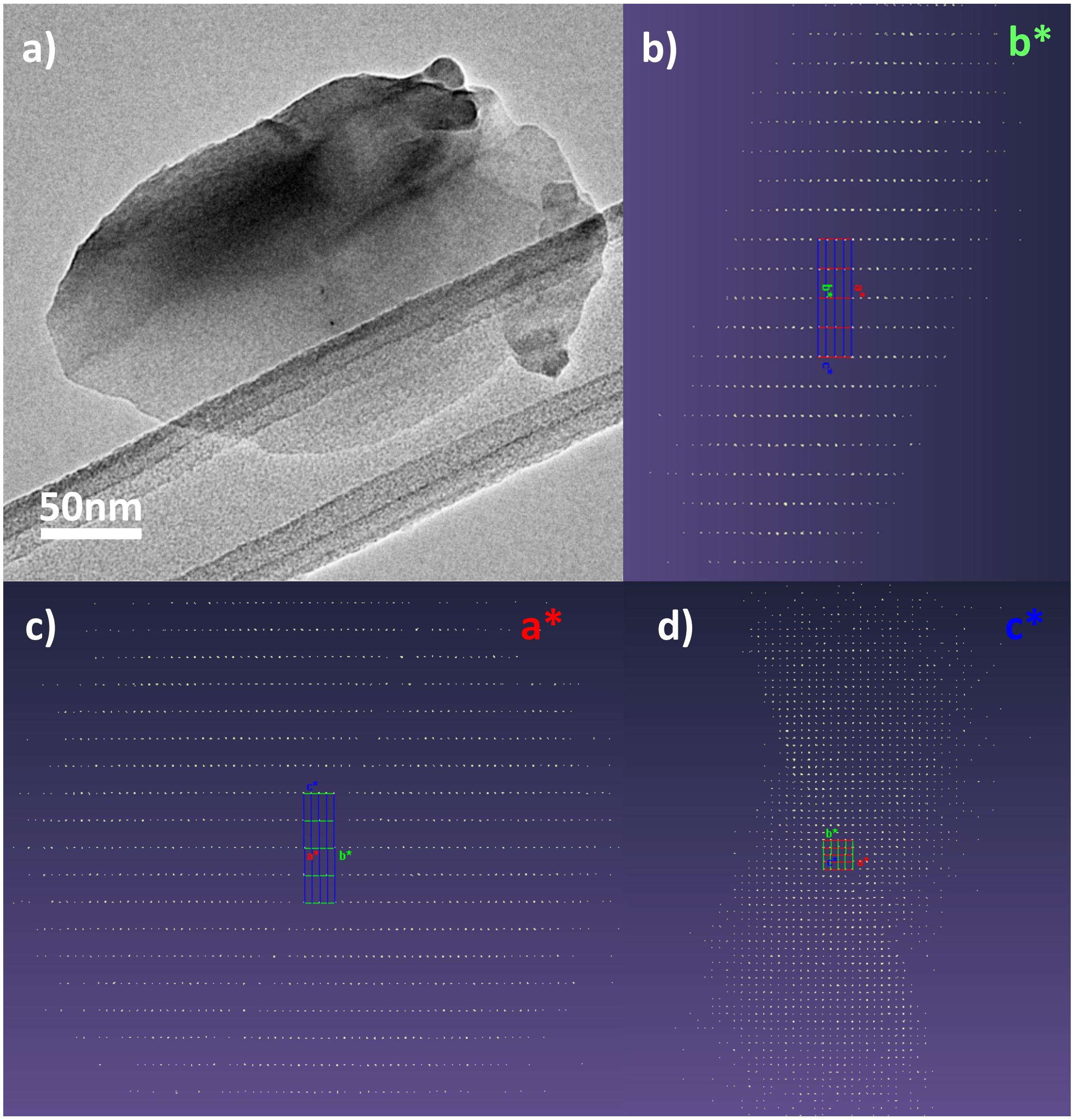

Figure SI8. a) Transmission Electron Micrograph of one of the crystals of phase A ($Ba_5Y_{13}[SiO_4]_8O_{8.5}$) used for collecting CRED data. b), c) and d) plots, along the axes *a**, *b** and *c**, of the portion of reciprocal space reconstructed with reflections extracted from the crystal shown in a). At the centre of panels b), c) and d) the unit cell is reported as aid for the reader. Images are generated using APEXIII from Bruker[15].

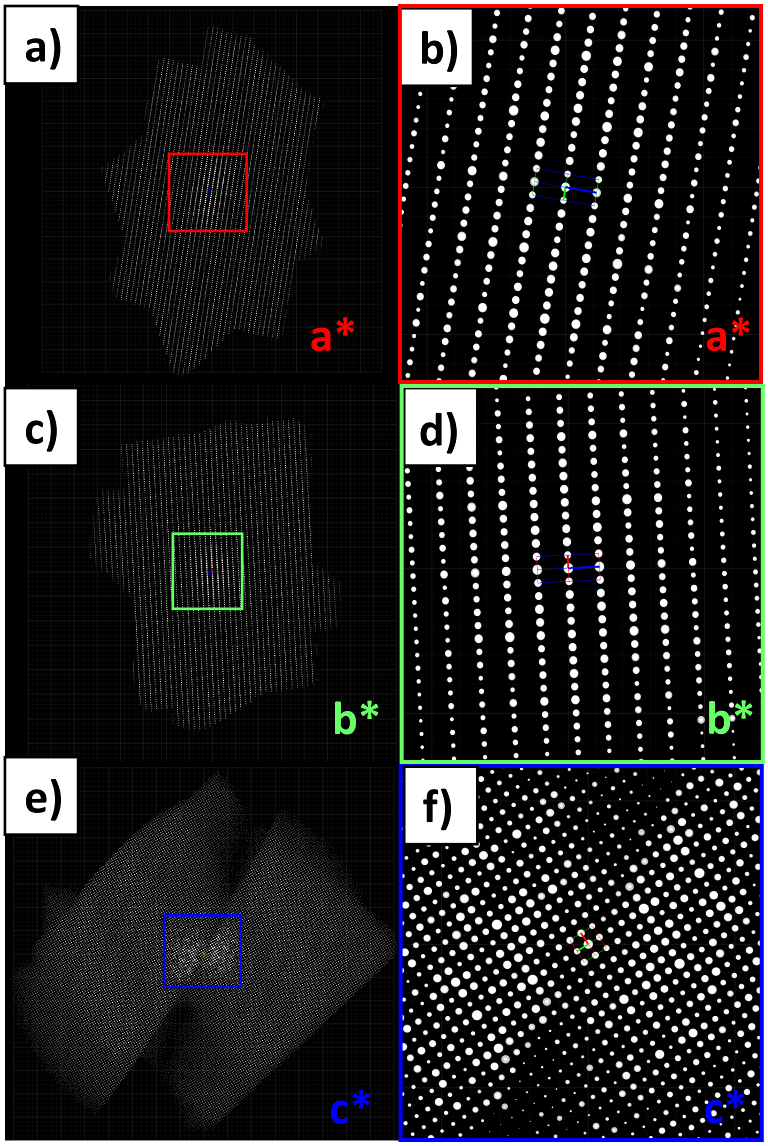

Figure SI9. a), c) and e) Plots, along the axes *a**, *b** and *c**, of the portion of reciprocal space obtained merging four datasets of phase A ($Ba_5Y_{13}[SiO_4]_8O_{8.5}$). b), d) and f) Zoom in of the rectangular central areas in a)-c)-e) panels. The unit cell has been added at the centre of panels b), d) and f) as aid for the reader.

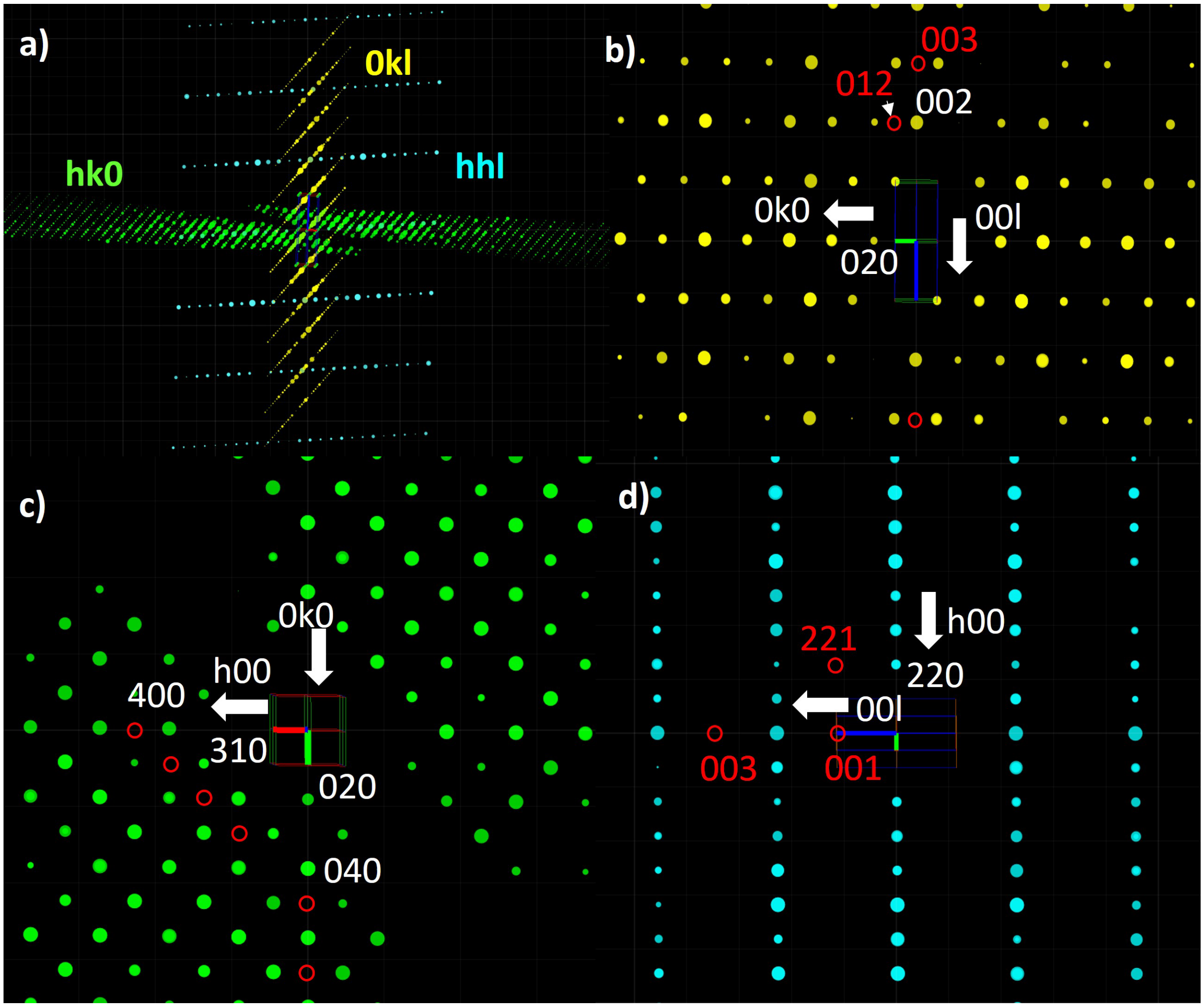

Figure SI10. a) Slicing of the 3D reciprocal space of Figure SI9 into the 0*kl*, *hk*0 and *hhl* planes. b), c) and d) plots of the reflections found in the 0*kl*, *hk*0, *hhl* planes respectively. Main axes for each plane are represented with white arrows. Some reflections are labelled in white while some systematic absences are highlighted with red circles and labelled in red.

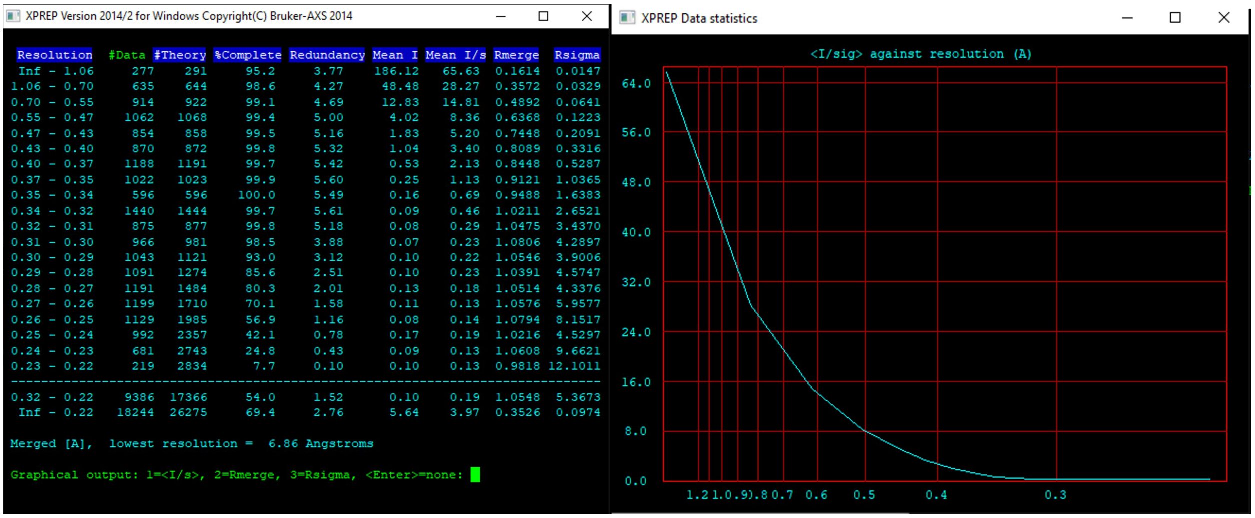

Figure SI11. Left: intensity statistics for the of phase A ($Ba_5Y_{13}[SiO_4]_8O_{8.5}$) CRED dataset analyzed with XPREP[7]. The statistics was used to choose the resolution for the data refinement. Right: plot of average I/sig against the resolution. The vast majority of data are enclosed in the [0.55,∞], therefore the chosen resolution was 0.55 and the rest of the data were truncated.

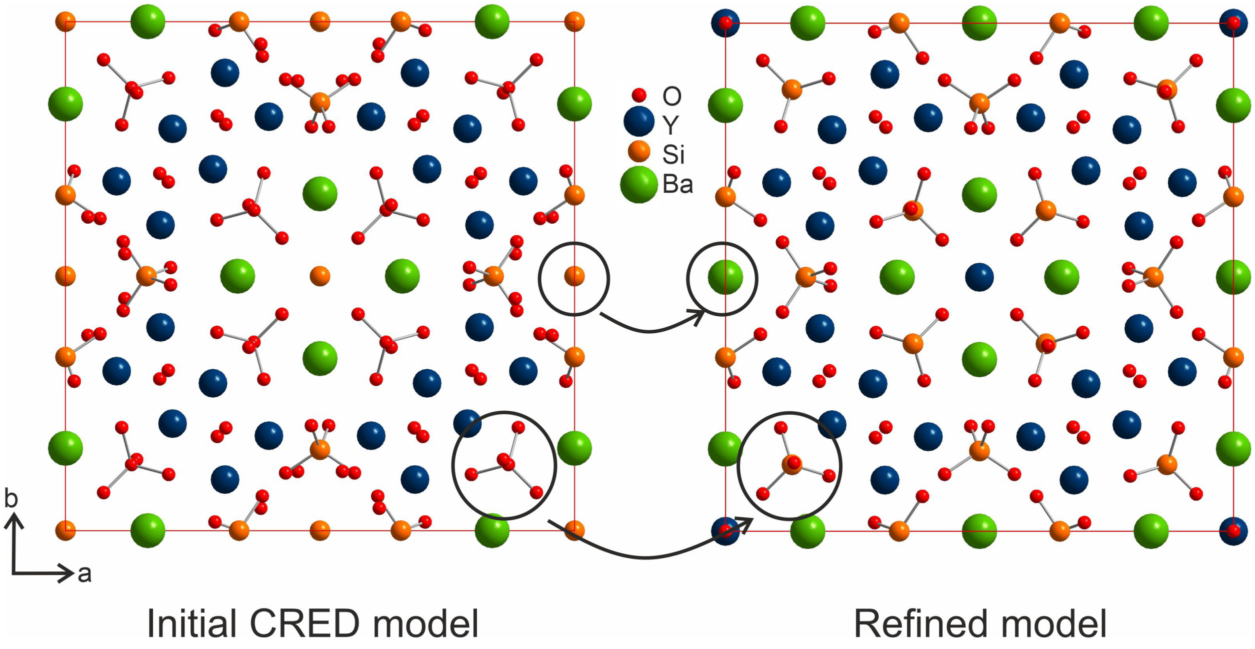

Figure SI12. ShelXT[8] solution from CRED data (left) of the structure of phase A ($Ba_5Y_{13}[SiO_4]_8O_{8.5}$) compared with the refined one (right). Despite the incorrect element assignment, the atomic sites were located correctly, and the tetrahedral geometry of silicate groups was evident.

Table SI3. Crystallographic data and structure refinement details on CRED data of $Ba_5Y_{13}[SiO_4]_8O_{8.5}$.

Chemical formula	$Ba_{10}Y_{26}Si_{16}O_{81}$
Data collection Temperature, K	300
Wavelength, Å	0.0251
Space group	$I\bar{4}2m$
Lattice parameters, Å	*a*=*b*=19.41(3), c=5.44(4)
Resolution, Å	0.55
Completeness, %	98.0
R_{int}, %	21.78
R_1, %, wR_2,%, GoF for $F^2>2s(F^2)$	19.96, 57.06, 3.292
Shift, Å	0.000
Reflections (observed)	8170 (3281)
Parameters (restraints)	39 (0)

SI4. Rietveld refinement of phase A

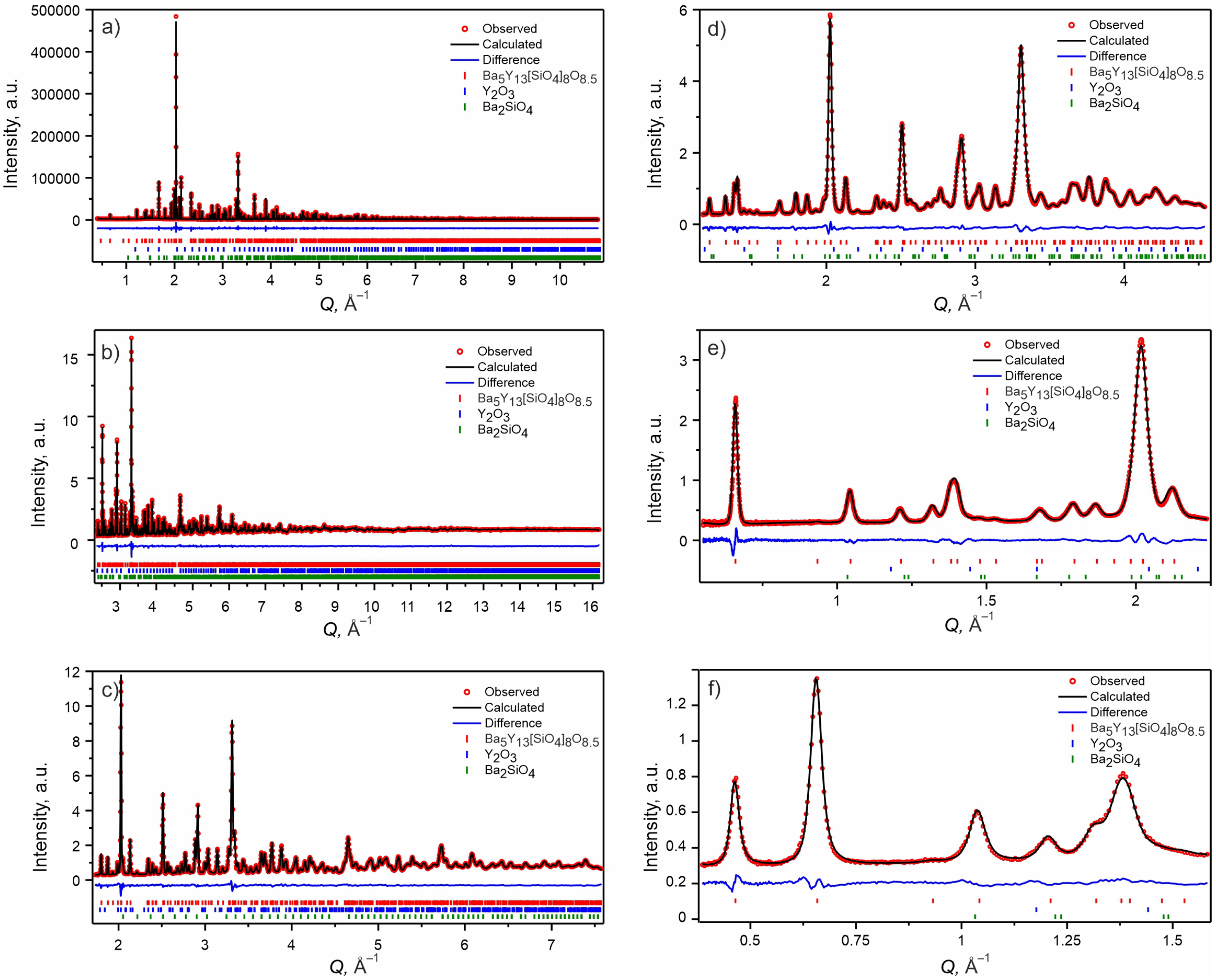

Figure SI13. Combined Rietveld refinement against (a) synchrotron XRD (I11, Diamond Light Source, MAC detector, λ = 0.82387Å) and TOF neutron diffraction from Bank 5 to Bank 1 (b-f) of $Ba_5Y_{13}[SiO_4]_8O_{8.5}$ ($I\bar{4}2m$, Z = 2) with R_{wp}= 3.68% and χ2= 3.05 for 154 refined parameters. Observed, calculated and difference intensity is drawn in red circles, black line and blue line, respectively. Bragg reflections for $Ba_5Y_{13}[SiO_4]_8O_{8.5}$ (93.55 wt. %), Y_2O_3 (4.84 wt. %) and Ba_2SiO_4 (1.61 wt. %) are shown as red, blue and green ticks, respectively. In the case of the SPXRD patterns, the Chebyshev polynomial function was applied to fit the background profile, and two broad peaks resulting from background from air and the capillary were described using two additional Pseudo-Voight functions. For each of the banks 1-5, a Chebyshev polynomial function of 8 terms was used to describe the background. The combined refinement has R_{wp}= 3.68% and χ2= 3.05 for 154 refined parameters for the structure of $Ba_5Y_{13}[SiO_4]_8O_{8.5}$.

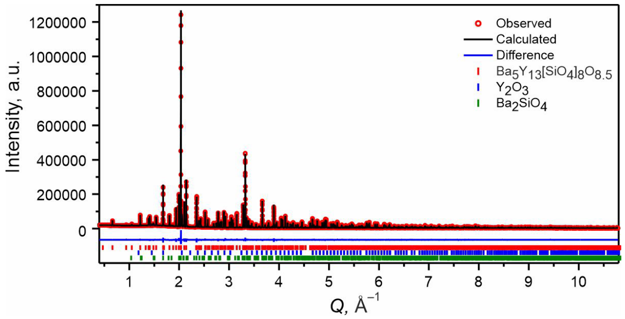

Figure SI14. Rietveld refinement of synchrotron XRD (I11, Diamond Light Source, PSD detector, λ = 0.82387Å) collected at 100K for $Ba_5Y_{13}[SiO_4]_8O_{8.5}$ ($I\bar{4}2m$, Z = 2). Observed, calculated and difference intensity is drawn in red circles, black line and blue line, respectively. Bragg reflections for $Ba_5Y_{13}[SiO_4]_8O_{8.5}$ (93.55 wt. %), Y_2O_3 (4.84 wt. %) and Ba_2SiO_4 (1.61 wt. %) are shown as red, blue and green ticks, respectively.

Table SI4. Crystallographic data and refinement details for $Ba_5Y_{13}[SiO_4]_8O_{8.75}$ from Rietveld refinements of the synchrotron PXRD data collected at room temperature and at 100K and from TOF neutron diffraction.

Radiation	NPD bank 1	NPD bank 2	NPD bank 3	NPD bank 4	NPD bank 5	SXRD	
Refined composition	$Ba_5Y_{13}[SiO_4]_8O_{8.5}$						
Formula weight, g mol^{-1}	2715.071						
Space group	$I\bar{4}2m$						
Z	2						
Lattice parameters, Å	*a* = 18.92732(1); *c* = 5.357307(6)						*a* = 18.90366(2); *c* = 5.351310(9)
Cell volume, Å^3	1919.221(3)						1912.282(6)
Density, g cm^{-3}	4.698						4.715
Measurement temperature, K	298						100
Angle, ° / Wavelength, Å	10.40	25.99	52.21	92.59	146.72	0.823801	0.82385
d spacing range, Å	3.9717-16.2919	2.8084-11.4034	1.3838-5.3664	0.8293-3.6223	0.3883-2.6552	0.5826-15.7345	0.5826-15.7371
TOF, µsec. / 2ϑ, ° range	2922.8353-11989.4653	4999.9476-20302.1522	4642.1475-18002.2999	4466.0756-19506.9939	1865.7364-19596.3778	3-90	3-90
TOF, µsec. / 2ϑ, ° step	3.5098	1.2009	1.1007	1.1009	0.5504	0.004	0.004
No. of refined parameters	154						90
R_p, %	1.91	3.11	2.49	2.58	2.56	4.81	3.10
R_{wp}, %	2.28	3.12	2.71	2.39	2.25	6.34	4.38
R_{exp}, %	0.78	1.07	0.79	0.52	0.72	2.17	1.03
$\chi 2$	2.92	2.92	3.39	4.57	3.11	2.92	4.26

Table SI5. Site coordinates and isotropic thermal parameters for the atoms in the structure of $Ba_5Y_{13}[SiO_4]_8O_{8.5}$ ($I\bar{4}2m$, Z = 2) based on combined Rietveld refinement of synchrotron and neutron TOF powder diffraction data recorded at room temperature (298 K). Site occupancy parameters for each atom were checked separately to ensure the correct assignment of species, and the occupancies were assigned and full or half-occupied within three standard deviations.

Site	Occ.	Wyck.	*x/a*	*y/b*	*z/c*	B_{iso} (Å^2)
O1	1	16*j*	0.06886(6)	0.38769(6)	0.0853(2)	0.70(2)
Y1	1	16*j*	0.10086(3)	0.31381(3)	0.4508(1)	0.387(9)
O2	1	16*j*	0.10912(6)	0.20366(6)	0.3034(2)	0.75(2)
O3	1	16*j*	0.20482(5)	0.31538(5)	0.2320(2)	0.41(2)
O4	1	16*j*	0.20642(6)	0.48324(6)	0.2547(2)	0.58(2)
O5	1	8*i*	0.07352(7)	*x*	0.3559(3)	1.78(4)
Si1	1	8*i*	0.13098(7)	*x*	0.4491(3)	0.57(3)
Y2	1	8*i*	0.20972(2)	*x*	0.0076(1)	0.45(1)
O6	1	8*i*	0.36543(6)	*x*	0.2524(3)	1.09(3)
Ba1	0.25	8*h*	0	1/2	0.2857(5)	0.64(3)
Ba2	1	8*f*	0.16192(3)	0	0	0.81(1)
Si2	1	8*f*	0.34199(9)	0	0	0.42(3)
Y3	0.5	4*e*	0	0	0.4278(4)	0.73(3)
O7	0.5	2*a*	0	0	0	0.4(1)

Table SI6. Complete list of interatomic distances (Å for the atoms in the structure of $Ba_5Y_{13}[SiO_4]_8O_{8.5}$ ($I\bar{4}2m$, Z = 2) based on combined Rietveld refinement of synchrotron and neutron TOF powder diffraction data recorded at room temperature (298 K).

Atoms			*d*, Å	Atoms			*d*, Å	Atoms			*d*, Å
O1	1x	Si2	1.6295(14)	O3	1x	Y1	2.2126(13)	O6	1x	Si1	1.647(3)
	1x	Y1	2.3473(13)		1x	Y2	2.2378(13)		1x	Y2	2.4242(19)
	1x	Y1	2.4811(13)		1x	Y1	2.2906(12)		1x	O5	2.697(3)
	1x	O4	2.5578(17)		1x	Y2	2.3429(12)		2x	O2	2.786(2)
	1x	Ba1	2.5839(13)		2x	O3	2.7347(4)		2x	Ba2	2.9146(13)
	1x	O4	2.6972(17)		1x	O2	2.8190(16)		2x	O3	3.183(2)
	1x	Ba1	2.7113(15)		1x	O3	2.966(2)	Ba1	1x	Ba1	0.384(4)
	1x	O1	2.760(2)		1x	O1	3.0192(16)		1x	Ba1	2.295(4)
	2x	O1	2.9214(9)		1x	O4	3.1675(16)		2x	O1	2.5839(13)
	1x	Ba1	2.9653(16)		1x	O6	3.183(2)		2x	Ba1	2.679083(4)
	1x	O3	3.0192(16)	O4	1x	Si2	1.6324(14)		2x	O1	2.7113(15)
	1x	Ba1	3.1899(18)		1x	Y1	2.3051(12)		2x	O1	2.9653(16)
Y1	1x	O3	2.2126(13)		1x	Y1	2.5214(12)		1x	Ba1	3.063(4)
	1x	O2	2.2375(13)		1x	O1	2.5578(17)		2x	O1	3.1899(18)
	1x	O3	2.2906(12)		1x	O1	2.6972(17)	Ba2	2x	O2	2.7419(12)
	1x	O4	2.3051(12)		1x	O4	2.701(2)		2x	O4	2.8321(13)
	1x	O1	2.3473(13)		1x	O4	2.812(2)		2x	O5	2.8877(13)
	1x	O1	2.4811(13)		1x	Ba2	2.8321(13)		2x	O6	2.9146(13)
	1x	O4	2.5214(12)		1x	O2	2.9488(16)		1x	O7	3.0664(4)
	1x	Si2	3.1262(5)		1x	O3	3.1675(16)	Si2	2x	O1	1.6295(14)
O2	1x	Si1	1.6281(16)	O5	1x	Si1	1.617(3)		2x	O4	1.6324(14)
	1x	Y1	2.2375(13)		1x	Y3	2.007(2)		2x	Y1	3.1262(5)
	1x	Y2	2.4866(13)		1x	Y3	2.289(2)	Y3	1x	Y3	0.772(3)
	1x	O2	2.532(2)		2x	O2	2.564(2)		2x	O5	2.007(2)
	1x	O5	2.564(2)		1x	O6	2.697(3)		2x	O5	2.289(2)
	1x	Ba2	2.7419(12)		1x	O7	2.733(2)		1x	O7	2.2931(15)
	1x	O6	2.786(2)		2x	Ba2	2.8877(13)		1x	O7	3.0650(15)
	1x	O3	2.8190(16)		2x	O5	3.193(3)	O7	2x	Y3	2.2931(15)
	1x	O4	2.9488(16)	Si1	1x	O5	1.617(3)		4x	O5	2.733(2)
					2x	O2	1.6281(16)		2x	Y3	3.0650(15)
					1x	O6	1.647(3)		4x	Ba2	3.0664(4)
					1x	Y2	3.1642(18)				
				Y2	2x	O3	2.2378(13)				
					2x	O3	2.3429(12)				
					1x	O6	2.4242(19)				
					2x	O2	2.4866(13)				
					1x	Si1	3.1642(18)				

Table SI7. Site coordinates and isotropic thermal parameters for the atoms in the structure of $Ba_5Y_{13}[SiO_4]_8O_{8.5}$ ($I\bar{4}2m$, Z = 2) based on Rietveld refinement of synchrotron powder diffraction data recorded at 100 K. * B_{iso} values for some oxygen atoms were fixed to 0.1 have positive values.

Site	Occ.	Wyck.	*x/a*	*y/b*	*z/c*	B_{iso}
O1	1	16*j*	0.0695(2)	0.3872(2)	0.0897(6)	0.23(7)
Y1	1	16*j*	0.10084(2)	0.31385(3)	0.4501(1)	0.233(9)
O2	1	16*j*	0.1093(2)	0.2035(2)	0.3010(6)	0.27(6)
O3	1	16*j*	0.2046(2)	0.3164(2)	0.2322(7)	0.08(6)
O4	1	16*j*	0.2061(2)	0.4831(2)	0.2576(6)	*0.1
O5	1	8*i*	0.0733(2)	*x*	0.346(1)	1.7(1)
Si1	1	8*i*	0.13088(8)	*x*	0.4429(5)	0.54(4)
Y2	1	8*i*	0.20958(2)	*x*	0.0058(2)	0.26(1)
O6	1	8*i*	0.3668(2)	*x*	0.2515(9)	0.3(1)
Ba1	0.25	8*h*	0	1/2	0.2776(5)	0.50(3)
Ba2	1	8*f*	0.16192(2)	0	0	0.50(7)
Si2	1	8*f*	0.3418(1)	0	0	0.39(3)
Y3	0.5	4*e*	0	0	0.4282(3)	0.48(3)
O7	0.5	2*a*	0	0	0	*0.1

Table SI8. Results of BVS calculation for the atoms in the structure of $Ba_5Y_{13}[SiO_4]_8O_{8.5}$ ($I\bar{4}2m$, Z = 2.

Atom	Coordination	Average bond length, Å	Valence	Occupancy corrected valence	Bond-valence sum
O1	4.00	2.5588	-2.000	-2.000	1.936
Y1	7.00	2.3412	3.000	3.000	3.048
O2	4.00	2.2735	-2.000	-2.000	2.106
O3	4.00	2.2705	-2.000	-2.000	2.043
O4	4.00	2.3225	-2.000	-2.000	1.922
O5	4.00	2.3386	-2.000	-2.000	2.169
Si1	4.00	1.6285	4.000	4.000	3.952
Y2	7.00	2.3635	3.000	3.000	2.851
O6	4.00	2.4740	-2.000	-2.000	1.679
Ba1	8.00	2.8641	2.000	2.000	2.001
Ba2	8.50	2.8718	2.000	2.000	1.839
Si2	4.00	1.6314	4.000	4.000	3.921
Y3	4.50	2.1739	3.000	3.000	3.293
O7	5.00	2.8071	-2.000	-1.000	0.965

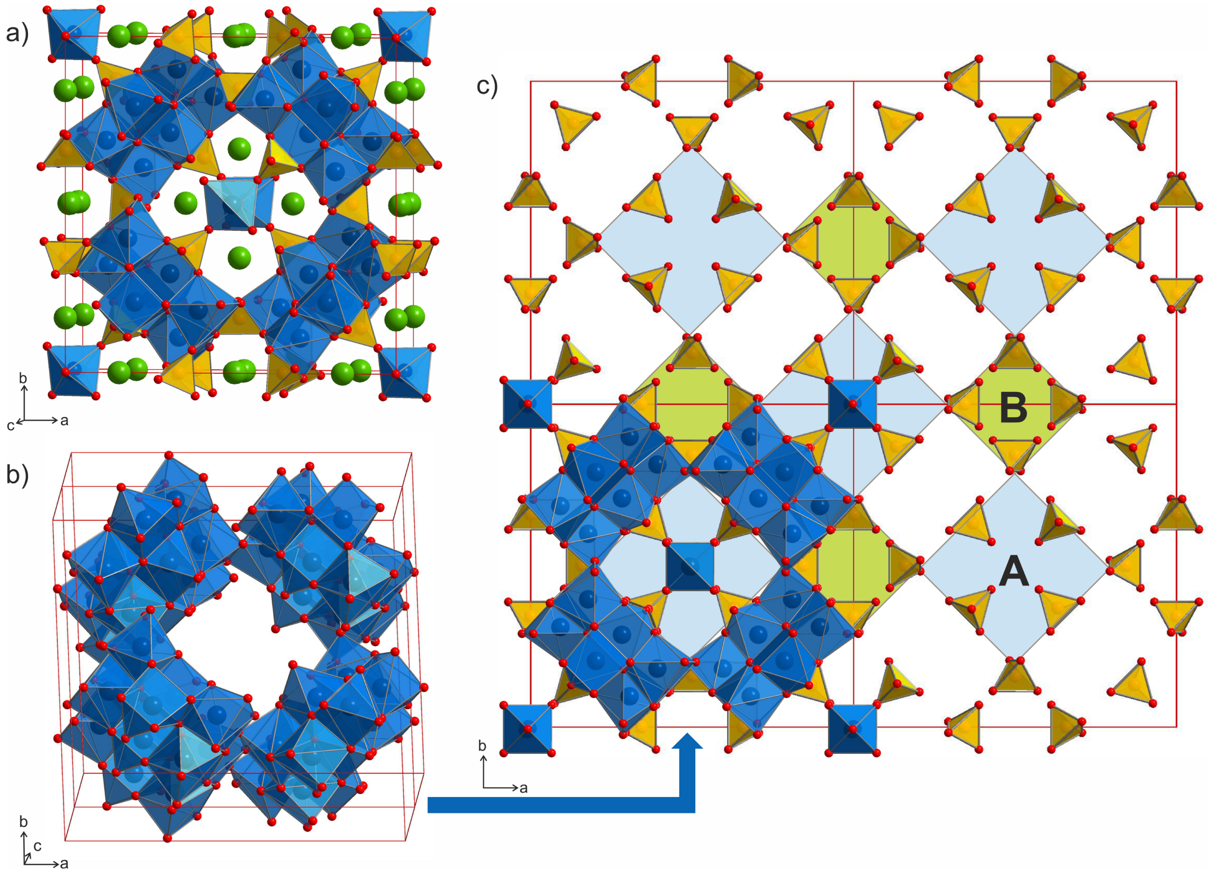

Figure SI15. (a) Structure of Phase A $Ba_5Y_{13}[SiO_4]_8O_{8.5}$ emphasizing coexistence of two networks formed by yttrium-centred polyhedra (b) and silicate groups (c). Silica tetrahedra and yttrium polyhedra are drawn in yellow and blue, respectively.

SI5. CRED analysis of phase B

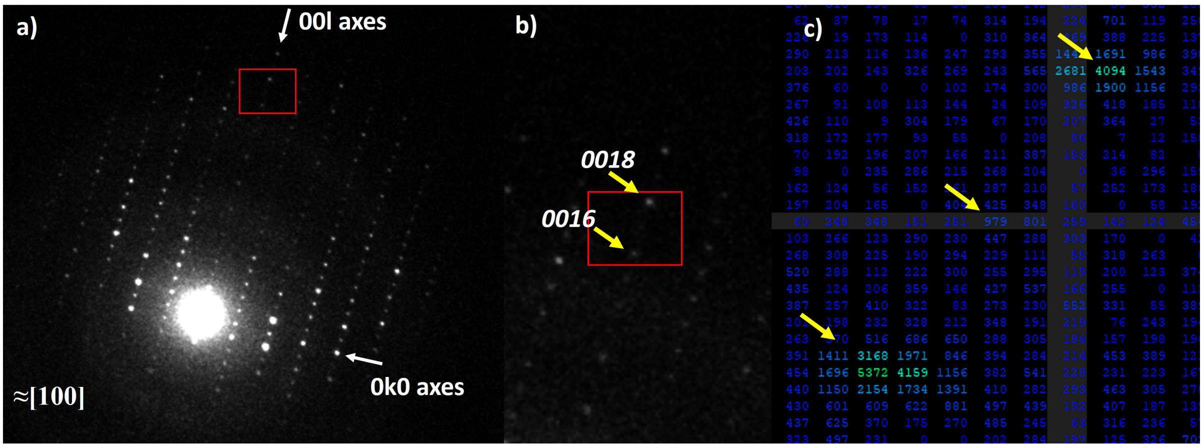

Figure SI16: a) TEM frame close to [100] zone axes measured on one of the particles of $Ba_3Y_2[Si_2O_7]_2$ (phase B). The 00*l* axis is highlighted with a white arrow. Area marked with a red rectangle is enlarged in (b) and even more in (c). (c) Pixel intensity analysis, performed using Redp[4,5], of the area within the red rectangle with peak intensities highlighted by yellow arrows. Note the small intensity region located in between 0 0 16 and 0 0 18 *hkl* values that violates reflection conditions for $P4_32_12$ space group.

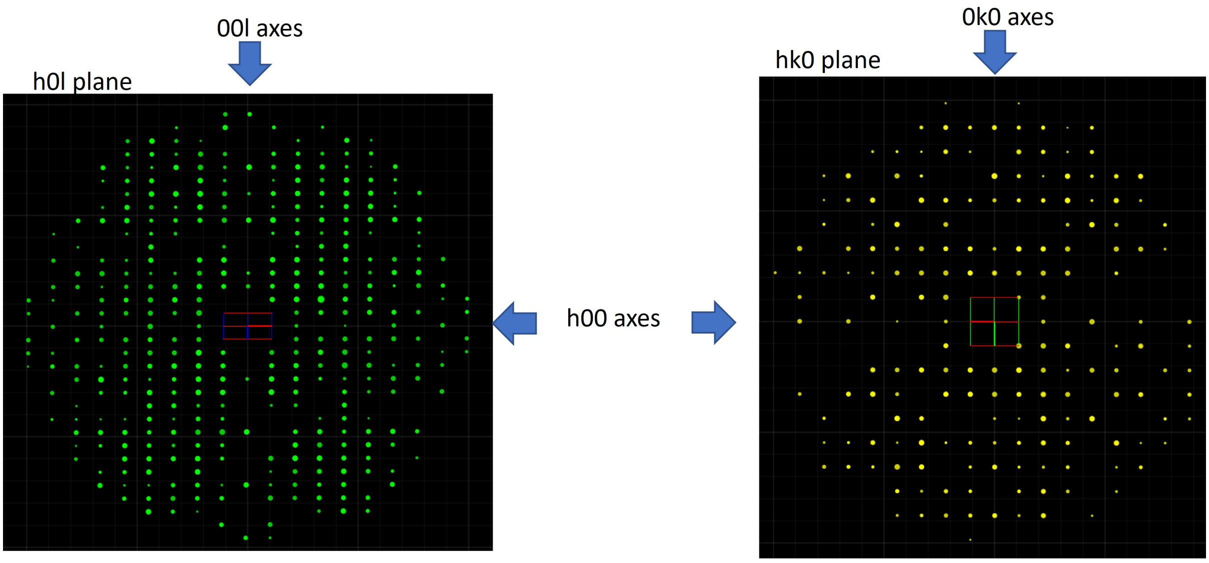

Figure SI17: Slicing of the 3D reciprocal space built using CRED data merging 10 datasets into the *hk*0 and *h*0*l* planes supplementing the analysis made in Figure 7.

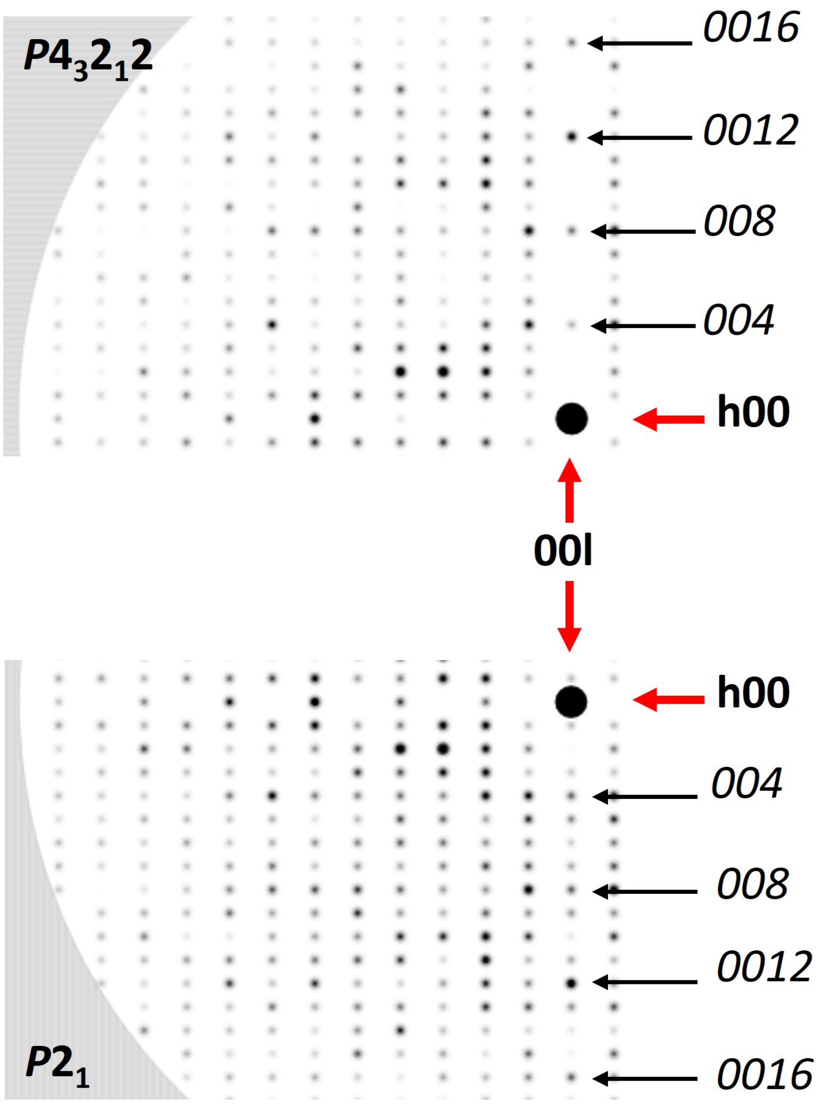

Figure SI18: Simulated diffraction pattern (Single Crystal 4 software from CrystalMaker Software Limited) for the higher symmetry $P4_32_12$ and lower symmetry $P2_1$ models using the same instrumental parameters as for the CRED measurement. The 000 point is marked with a black circle.

SI6. Rietveld refinement of Phase B

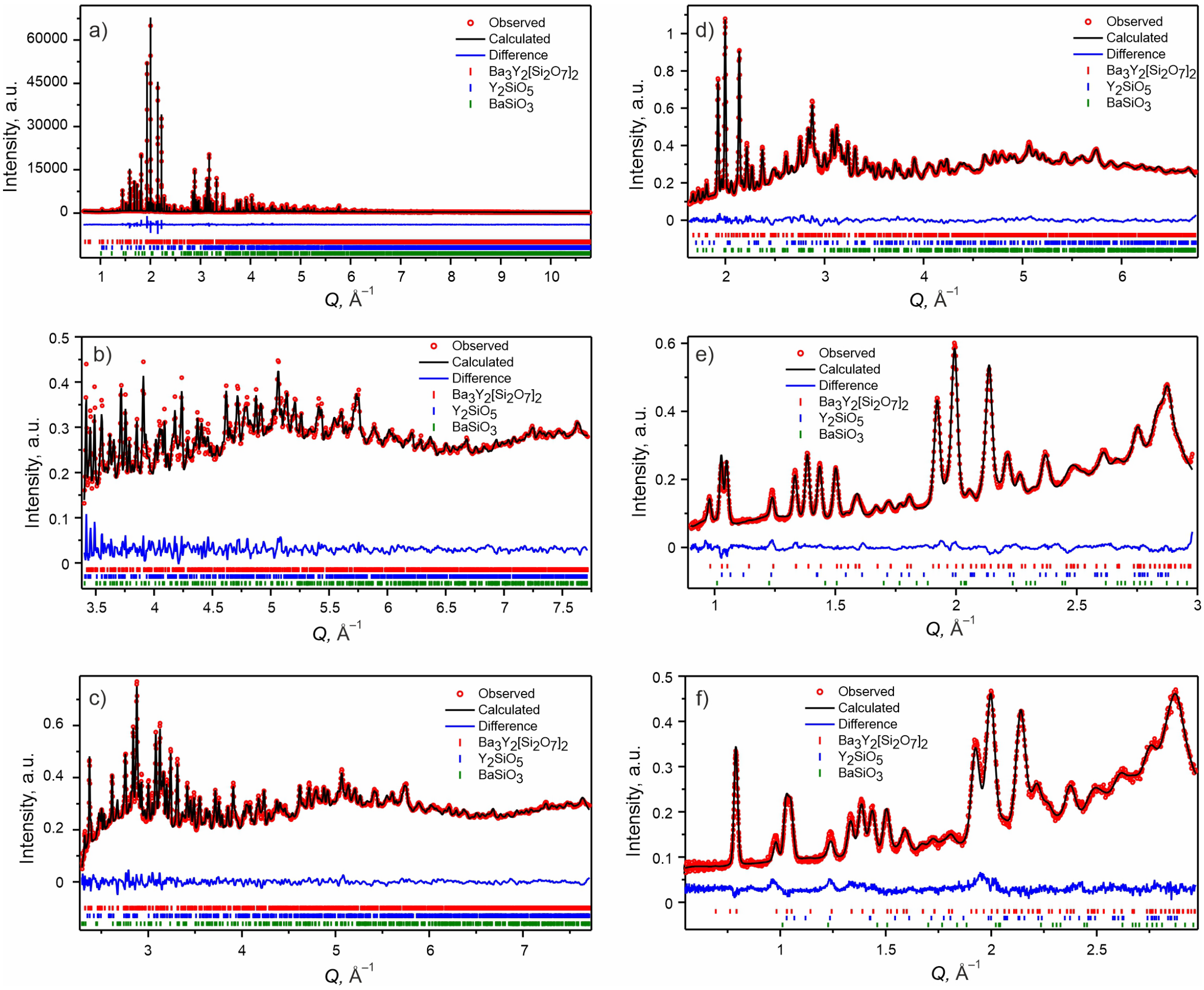

Figure SI19. Combined Rietveld refinement against (a) synchrotron XRD (I11, Diamond Light Source, MAC detector, λ = 0.82387Å) and TOF neutron diffractions from Bank 6 to Bank 2 (b-f) of $Ba_3Y_2[Si_2O_7]_2$ ($P2_1$, Z = 4) with R_{wp}= 5.19% and χ2= 2.92 for 413 refined parameters. Observed, calculated and difference intensity is drawn in red circles, black line and blue line, respectively. Bragg reflections for $Ba_3Y_2[Si_2O_7]_2$ (96.11 wt. %), Y_2SiO_5 (2.59 wt. %) and $BaSiO_3$ (1.30 wt. %) are shown as red, blue and green ticks, respectively. Chebyshev polynomial function was used to describe the background profiles of all datasets. Due to a presence of amorphous phase in the sample, TOF data had stronger background; in case of Bank 2, it was described using additional broad Pseudo-Voight peak combined with Chebyshev polynomial function.

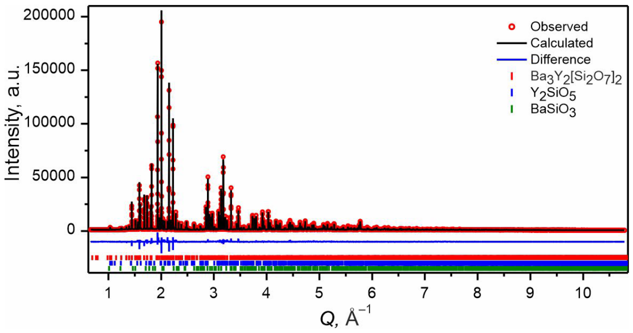

Figure SI20. Rietveld refinement of synchrotron XRD (I11, Diamond Light Source, MAC detector, λ = 0.82387Å) collected at 100K for $Ba_3Y_2[Si_2O_7]_2$ ($P2_1$, Z = 4). Observed, calculated and difference intensity is drawn in red circles, black line and blue line, respectively. Bragg reflections for $Ba_3Y_2[Si_2O_7]_2$ (96.11 wt. %), Y_2SiO_5 (2.59 wt. %) and $BaSiO_3$ (1.30 wt. %) are shown as red, blue and green ticks, respectively.

Table SI9. Crystallographic data and refinement details for $Ba_3Y_2[Si_2O_7]_2$ from Rietveld refinements of the synchrotron PXRD data collected at room temperature and at 100K and from TOF neutron diffraction.

Radiation	NPD bank 2	NPD bank 3	NPD bank 4	NPD bank 5	NPD bank 6	SXRD	
Refined composition	$Ba_3Y_2[Si_2O_7]_2$						
Formula weight, g mol^{-1}	926.2						
Space group	*P*2$_1$						
Z	4						
Lattice parameters	*a* = 16.47640(4) Å<br>*b* = 9.04150(5) Å<br>*c* = 9.04114(7) Å<br>β = 90.0122(9)°						*a* = 16.45139(4) Å<br>*b* = 9.02912(4) Å<br>*c* = 9.02884(6) Å<br>β = 90.0104(7)°
Cell volume, Å^3	1346.871(13)						1341.158(11)
Density, g cm^{-3}	4.568						4.587
Measurement temperature, K	298						100
Angle, ° / Wavelength, Å	17.98	34.96	63.62	91.37	154.46	0.824037	0.824037
d spacing range, Å	2.1150 – 11.7512	2.1083- 6.9668	0.9311 – 3.8371	0.8151 – 2.7411	0.8143- 1.8481	0.5827-9.4459	0.5827-9.4459
TOF, µsec. / 2ϑ, ° range	3131.3695-17404.0964	5899.5369-19505.3948	4539.2488-18701.5809	5423.3294-18228.3833	7373.0964-16734.9350	5-90	5-90
TOF, µsec. / 2ϑ, ° step	0.4889	0.5601	0.4826	0.4773	0.5953	0.004	0.004
No. of refined parameters	413						301
R_p, %	3.34	2.91	2.34	2.56	2.51	6.06	5.25
R_{wp}, %	4.53	3.35	2.99	2.87	2.79	8.31	7.73
R_{exp}, %	2.95	1.32	0.83	0.79	0.78	3.11	1.79
$\chi 2$	1.53	2.53	3.62	3.59	3.61	2.67	4.30

Table SI10. Site coordinates and isotropic thermal parameters for the atoms in the structure of $Ba_3Y_2[Si_2O_7]_2$ ($P2_1$, Z = 4) based on combined Rietveld refinement of synchrotron and neutron TOF powder diffraction data recorded at room temperature (298 K). Thermal parameters B_{iso} were refined constrained to the same value per atomic type for all split silicon and oxygen atomic sites. Atoms forming disilicate groups are listed together (the geometry of these groups was restrained). Labels *a* and *b* correspond to two equivalent disilicate groups.* The coordinates of Ba1 were kept fixed due to the floating origin of the $P2_1$ space group.

Site	Wyck.	*x/a*	*y/b*	*z/c*	B_{iso}
Ba1*	2*a*	0.08677	0.58991	0.24102	3.0(3)
Ba2	2*a*	0.5869(5)	0.8892(13)	0.2610(10)	1.9(2)
Ba3	2*a*	0.8337(6)	0.7447(16)	0.1017(10)	2.1(3)
Ba4	2*a*	0.3353(6)	0.7280(16)	0.3948(11)	2.2(2)
Y1	2*a*	0.3760(10)	0.062(2)	0.0661(16)	1.3(2)
Y2	2*a*	0.8788(8)	0.414(2)	0.4325(15)	1.2(2)
*M*1: 0.44(4)Ba5+0.56(4)Y5	2*a*	0.2420(7)	0.7182(18)	0.9021(13)	2.5(3)
*M*2: 0.47(4)Ba6+0.53(4)Y6	2*a*	0.9954(7)	0.0945(19)	0.2323(15)	2.0(3)
*M*3: 0.52(4)Ba7+0.48(4)Y7	2*a*	0.7450(7)	0.7524(19)	0.6045(13)	2.2(3)
M4: 0.58(4)Ba8+0.42(4)Y8	2*a*	0.4946(7)	0.3788(18)	0.2589(13)	1.6(2)
Disilicate 1 (*D*1), occupancies a:b = 0.61(2) : 0.39(2)					
Si1a	2*a*	0.449(4)	0.665(7)	0.016(6))	1.0(2)
Si2a	2*a*	0.674(4)	0.963(6)	0.917(7)	1.0(2)
Si1b	2*a*	0.443(6)	0.667(11)	0.055(10)	1.0(2)
Si2b	2*a*	0.676(6)	0.930(10)	0.902(12)	1.0(2)
O1a	2*a*	0.590(3)	0.329(5)	0.930(5)	2.1(1)
O2a	2*a*	0.512(3)	0.101(5)	0.837(5)	2.1(1)
O3a	2*a*	0.628(3)	0.069(6)	1.020(6)	2.1(1)
O4a	2*a*	0.496(3)	0.180(5)	0.131(6)	2.1(1)
O5a	2*a*	0.336(3)	0.285(6)	0.074(6)	2.1(1)
O6a	2*a*	0.767(3)	0.009(6)	0.940(6)	2.1(1)
O7a	2*a*	0.350(3)	0.506(5)	0.248(5)	2.1(1)
O1b	2*a*	0.550(4)	0.312(9)	0.841(8)	2.1(1)
O2b	2*a*	0.470(5)	0.079(8)	0.921(8)	2.1(1)
O3b	2*a*	0.631(5)	0.081(10)	0.879(9)	2.1(1)
O4b	2*a*	0.588(4)	0.214(8)	0.118(8)	2.1(1)
O5b	2*a*	0.334(5)	0.320(10)	-0.038(9)	2.1(1)
O6b	2*a*	0.774(6)	-0.025(10)	0.883(11)	2.1(1)
O7b	2*a*	0.352(4)	0.336(7)	0.238(7)	2.1(1)
Disilicate 2 (*D*2), occupancies a:b = 0.60(2) : 0.40(2)					
Si3a	2*a*	0.934(10)	0.79(2)	0.47(2)	1.0(2)
Si4a	2*a*	0.192(3)	0.552(5)	0.577(6)	1.0(2)
Si3b	2*a*	0.938(16)	0.80(3)	0.48(3)	1.0(2)
Si4b	2*a*	0.167(4)	0.538(8)	0.644(7)	1.0(2)
O8a	2*a*	0.862(3)	0.923(6)	0.521(6)	2.1(1)
O9a	2*a*	0.905(2)	0.665(6)	0.346(5)	2.1(1)
O10a	2*a*	1.068(3)	0.158(5)	0.409(5)	2.1(1)
O11a	2*a*	0.024(3)	0.860(6)	0.441(6)	2.1(1)
O12a	2*a*	0.287(4)	0.512(6)	0.582(8)	2.1(1)

O13a	2*a*	0.176(3)	0.712(6)	0.507(6)	2.1(1)
O14a	2*a*	0.162(3)	0.552(7)	0.744(6)	2.1(1)
O8b	2*a*	0.857(5)	0.896(11)	0.471(8)	2.1(1)
O9b	2*a*	0.912(4)	0.627(9)	0.504(9)	2.1(1)
O10b	2*a*	1.003(4)	0.358(8)	0.382(7)	2.1(1)
O11b	2*a*	-0.021(4)	0.809(9)	0.323(7)	2.1(1)
O12b	2*a*	0.267(4)	0.551(8)	0.671(7)	2.1(1)
O13b	2*a*	0.136(4)	0.704(8)	0.613(8)	2.1(1)
O14b	2*a*	0.127(4)	0.487(7)	0.797(7)	2.1(1)
Disilicate 3 (*D*3), occupancies a:b = 0.60(2) : 0.40(2)					
Si5a	2*a*	0.200(3)	0.980(7)	0.197(7)	1.0(2)
Si6a	2*a*	0.939(3)	0.397(6)	0.004(5)	1.0(2)
Si5b	2*a*	0.176(5)	0.973(9)	0.215(10)	1.0(2)
Si6b	2*a*	0.924(5)	0.365(9)	0.032(10)	1.0(2)
O15a	2*a*	0.798(3)	0.372(6)	0.647(5)	2.1(1)
O16a	2*a*	0.754(3)	0.640(5)	0.789(5)	2.1(1)
O17a	2*a*	0.888(3)	0.506(7)	0.901(7)	2.1(1)
O18a	2*a*	0.756(3)	0.400(6)	0.935(5)	2.1(1)
O19a	2*a*	0.106(3)	0.912(6)	0.836(6)	2.1(1)
O20a	2*a*	0.951(3)	0.223(5)	-0.015(5)	2.1(1)
O21a	2*a*	0.029(3)	0.456(5)	1.018(6)	2.1(1)
O15b	2*a*	0.813(5)	0.457(8)	0.609(8)	2.1(1)
O16b	2*a*	0.859(4)	0.645(7)	0.811(8)	2.1(1)
O17b	2*a*	0.876(4)	0.361(10)	0.881(7)	2.1(1)
O18b	2*a*	0.740(5)	0.440(9)	0.871(10)	2.1(1)
O19b	2*a*	0.099(5)	0.996(10)	0.856(9)	2.1(1)
O20b	2*a*	0.886(4)	0.208(8)	0.078(8)	2.1(1)
O21b	2*a*	0.021(5)	0.366(9)	0.997(9)	2.1(1)
Disilicate 4 (*D*4), occupancies a:b = 0.50(2) : 0.50(2)					
Si7a	2*a*	0.686(18)	0.53(3)	0.31(4)	1.0(2)
Si8a	2*a*	0.441(5)	0.097(9)	0.448(10)	1.0(2)
Si7b	2*a*	0.688(18)	0.53(3)	0.31(4)	1.0(2)
Si8b	2*a*	0.441(5)	0.065(9)	0.462(9)	1.0(2)
O22a	2*a*	0.514(3)	0.445(6)	0.519(5)	2.1(1)
O23a	2*a*	0.621(4)	0.560(8)	0.680(7)	2.1(1)
O24a	2*a*	0.646(3)	0.490(7)	0.145(7)	2.1(1)
O25a	2*a*	0.768(3)	0.620(6)	0.317(5)	2.1(1)
O26a	2*a*	0.613(4)	0.624(7)	0.410(8)	2.1(1)
O27a	2*a*	0.498(3)	0.736(6)	0.563(6)	2.1(1)
O28a	2*a*	0.712(3)	0.361(6)	0.383(6)	2.1(1)
O22b	2*a*	0.464(4)	0.571(8)	0.511(7)	2.1(1)
O23b	2*a*	0.585(3)	0.468(8)	0.682(7)	2.1(1)
O24b	2*a*	0.695(4)	0.483(7)	0.140(7)	2.1(1)
O25b	2*a*	0.712(3)	0.705(7)	0.336(5)	2.1(1)
O26b	2*a*	0.599(4)	0.497(7)	0.393(7)	2.1(1)
O27b	2*a*	0.601(4)	0.728(7)	0.562(7)	2.1(1)
O28b	2*a*	0.760(3)	0.424(6)	0.355(6)	2.1(1)

Table SI11. List of interatomic distances (Å) for the atoms in the structure of $Ba_3Y_2[Si_2O_7]_2$ ($P2_1$, Z = 4) based on combined Rietveld refinement of synchrotron and neutron TOF powder diffraction data recorded at room temperature (298 K). For silicon atoms, the coordination of a corresponding disilicate group is given.

Atoms			*d*, Å	Atoms			*d*, Å	Atoms			*d*, Å
Ba1	1x	O20a	2.46(4)	*M*1: Ba5/Y5	1x	O20b	2.12(6)	Si1a	1x	Si1b	0.37(11)
	1x	O21a	2.54(5)		1x	O18a	2.21(5)		1x	O3a	1.57(8)
	1x	O6b	2.76(10)		1x	O6a	2.38(5)		1x	O2a	1.59(7)
	1x	O17b	2.76(9)		1x	O14a	2.45(6)		1x	O4a	1.60(8)
	1x	O11b	2.76(8)		1x	O12b	2.61(7)		1x	O1a	1.69(8)
	1x	O8a	2.76(6)		1x	O3a	2.63(5)		1x	Si2a	2.81(9)
	1x	O10b	2.81(7)		1x	O24b	2.64(6)	Si2a	1x	Si2b	0.33(11)
	1x	O6a	3.00(6)		1x	O4b	2.80(6)		1x	O3a	1.54(8)
	1x	O13a	3.03(5)		1x	O18b	2.88(9)		1x	O6a	1.60(9)
	1x	O20b	3.11(8)		1x	O19a	2.91(5)		1x	O7a	1.60(8)
	1x	O21b	3.19(8)		1x	O6b	2.95(10)		1x	O5a	1.61(7)
Ba2	1x	O5b	2.48(8)		1x	O28b	2.98(6)		1x	Si1a	2.81(9)
	1x	O1b	2.54(7)		1x	O14b	2.98(6)	Si1b	1x	Si1a	0.37(11)
	1x	O22a	2.64(5)		1x	O28a	2.98(6)		1x	O3b	1.57(13)
	1x	O25b	2.73(6)		1x	O17b	3.05(8)		1x	O1b	1.62(13)
	1x	O12a	2.75(6)		1x	O24a	3.10(6)		1x	O2b	1.64(12)
	1x	O22b	2.77(7)		1x	Si4b	3.10(6)		1x	O4b	1.70(11)
	1x	O26a	2.78(7)		1x	Si6b	3.11(8)		1x	Si2b	2.94(14)
	1x	O3a	2.80(5)		1x	O3b	3.13(9)	Si2b	1x	Si2a	0.33(11)
	1x	O12b	2.88(7)		1x	O13b	3.14(7)		1x	O3b	1.57(13)
	1x	O23b	2.97(6)		1x	Si2a	3.15(6)		1x	O5b	1.59(13)
	1x	O27b	3.09(7)	*M*2: Ba6/Y6	1x	O10a	2.08(5)		1x	O7b	1.60(13)
	1x	O2a	3.19(5)		1x	O14b	2.26(6)		1x	O6b	1.67(14)
Ba3	1x	O14b	2.46(6)		1x	O17a	2.40(6)		1x	Si1b	2.94(14)
	1x	O25a	2.49(5)		1x	O16b	2.48(7)	Si3a	1x	Si3b	0.1(4)
	1x	O19b	2.54(9)		1x	O20b	2.49(7)		1x	O10a	1.60(19)
	1x	O9a	2.60(5)		1x	O21a	2.62(6)		1x	O11a	1.63(18)
	1x	O21b	2.78(8)		1x	O20a	2.62(5)		1x	O9a	1.70(19)
	1x	O16b	2.81(7)		1x	O14a	2.64(5)		1x	O8a	1.73(19)
	1x	O5b	2.91(9)		1x	O11b	2.72(8)		1x	Si4a	3.18(19)
	1x	O25b	2.94(5)		1x	O10b	2.74(7)	Si4a	1x	Si4b	0.76(8)
	1x	O17a	2.96(6)		1x	O13b	2.77(7)		1x	O14a	1.59(7)
	1x	O6a	3.01(5)		1x	O9b	2.85(8)		1x	O12a	1.60(7)
	1x	O6b	3.04(10)		1x	O11a	2.88(6)		1x	O13a	1.60(7)
	1x	O14a	3.11(6)		1x	Si4b	2.94(7)		1x	O8a	1.72(8)
	1x	O21a	3.15(5)		1x	O21b	2.94(8)		1x	Si3a	3.18(19)
	1x	O11b	3.17(7)		1x	Si6a	2.99(5)	Si3b	1x	Si3a	0.1(4)
Ba4	1x	O7a	2.41(5)		1x	O17b	3.16(8)		1x	O8b	1.6(3)
	1x	O28a	2.47(6)		1x	Si5b	3.18(8)		1x	O11b	1.6(3)
	1x	O15a	2.58(5)	*M*3: Ba7/Y7	1x	O16a	1.96(5)		1x	O9b	1.6(3)
	1x	O23b	2.63(7)		1x	O7b	2.27(7)		1x	O10b	1.7(3)
	1x	O12a	2.71(6)		1x	O27b	2.42(6)		1x	Si4b	3.0(3)
	1x	O22b	2.75(7)		1x	O25b	2.53(5)	Si4b	1x	Si4a	0.76(8)
	1x	O13a	2.82(5)		1x	O8b	2.57(9)		1x	O14b	1.59(9)
	1x	O3b	2.86(8)		1x	O8a	2.59(6)		1x	O13b	1.60(10)

	1x	O1b	2.95(8)		1x	O23a	2.76(7)		1x	O12b	1.68(10)
	1x	O27a	3.08(5)		1x	O16b	2.82(7)		1x	O8b	1.70(11)
	1x	O23a	3.16(7)		1x	O25a	2.89(5)		1x	Si3b	3.0(3)
	1x	O12b	3.18(7)		1x	O15b	2.90(8)	Si5a	1x	Si5b	0.42(10)
Y1	1x	O2b	2.04(8)		1x	O12a	2.93(7)		1x	O18a	1.57(8)
	1x	O24a	2.05(7)		1x	O26a	3.03(7)		1x	O16a	1.64(7)
	1x	O5a	2.12(5)		1x	O7a	3.09(5)		1x	O15a	1.72(8)
	1x	O1a	2.18(5)		1x	O9b	3.11(7)		1x	O17a	1.72(8)
	1x	O18b	2.27(8)		1x	Si5a	3.18(6)		1x	Si6a	3.02(7)
	1x	O23a	2.30(7)		1x	O10a	3.20(5)	Si6a	1x	Si6b	0.46(10)
	1x	O24b	2.31(6)	*M*4: Ba8/Y8	1x	O27a	2.07(5)		1x	O21a	1.57(7)
	1x	O4a	2.33(5)		1x	O4a	2.14(5)		1x	O17a	1.60(8)
	1x	O23b	2.52(6)		1x	O2a	2.19(5)		1x	O20a	1.60(7)
	1x	O16a	2.61(5)		1x	O26b	2.36(7)		1x	O19a	1.63(7)
	1x	O5b	2.61(9)		1x	O7b	2.39(6)		1x	Si5a	3.02(7)
	1x	O18a	2.62(5)		1x	O22a	2.45(5)	Si5b	1x	Si5a	0.42(10)
	1x	O1b	2.70(8)		1x	O4b	2.50(7)		1x	O17b	1.59(12)
	1x	O7b	2.95(7)		1x	O2b	2.50(8)		1x	O15b	1.61(11)
	1x	O2a	3.07(5)		1x	O27b	2.64(6)		1x	O18b	1.62(11)
	1x	Si1a	3.11(7)		1x	O7a	2.65(4)		1x	O16b	1.67(10)
Y2	1x	O13b	1.96(8)		1x	O24a	2.88(6)		1x	Si6b	2.94(12)
	1x	O15b	1.97(8)		1x	O22b	2.91(7)	Si6b	1x	Si6a	0.46(10)
	1x	O11a	2.03(5)		1x	O3b	3.04(9)		1x	O17b	1.58(11)
	1x	O28b	2.08(5)		1x	Si8a	3.20(8)		1x	O19b	1.60(12)
	1x	O9b	2.10(8)						1x	O20b	1.61(11)
	1x	O13a	2.11(6)						1x	O21b	1.62(11)
	1x	O10b	2.16(7)						1x	Si5b	2.94(12)
	1x	O15a	2.38(5)					Si7a	1x	Si7b	0.1(4)
	1x	O9a	2.44(5)						1x	O25a	1.6(3)
	1x	O19a	2.44(6)						1x	O28a	1.7(3)
	1x	O19b	2.74(8)						1x	O24a	1.7(3)
	1x	O10a	2.77(5)						1x	O26a	1.7(3)
	1x	O25a	2.80(5)						1x	Si8a	3.1(3)
	1x	O28a	2.82(5)					Si8a	1x	Si8b	0.31(11)
									1x	O26a	1.58(11)
									1x	O23a	1.58(11)
									1x	O22a	1.59(10)
									1x	O27a	1.62(10)
									1x	Si7a	3.1(3)
								Si7b	1x	Si7a	0.1(4)
									1x	O28b	1.6(3)
									1x	O25b	1.6(3)
									1x	O24b	1.6(3)
									1x	O26b	1.7(3)
									1x	Si8b	3.0(3)
								Si8b	1x	Si8a	0.31(11)
									1x	O22b	1.59(11)
									1x	O26b	1.60(10)
									1x	O23b	1.62(10)
									1x	O27b	1.65(10)
									1x	Si7b	3.0(3)

Table SI12. Site coordinates and isotropic thermal parameters for the atoms in the structure of $Ba_3Y_2[Si_2O_7]_2$ ($P2_1$, Z = 4) based on Rietveld refinement of synchrotron powder diffraction data recorded at 100 K. Thermal parameters B_{iso} were refined constrained to the same value per atomic type for all split silicon and oxygen atomic sites. Atoms forming disilicate groups are listed together. Labels *a* and *b* correspond to two equivalent disilicate groups.* The coordinates of Ba1 were kept fixed due to the floating origin of the $P2_1$ space group The occupancy parameters were fixed according to the room temperature model.

Site	Wyck.	*x/a*	*y/b*	*z/c*	B_{iso}
Ba1*	2*a*	0.08677	0.58991	0.24102	1.9(2)
Ba2	2*a*	0.5873(5)	0.8872(13)	0.2666(10)	1.8(2)
Ba3	2*a*	0.8349(5)	0.7429(16)	0.0971(10)	1.7(2)
Ba4	2*a*	0.3341(5)	0.7308(16)	0.3931(10)	1.6(2)
Y1	2*a*	0.3789(9)	0.064(2)	0.0600(14)	0.8(2)
Y2	2*a*	0.8790(10)	0.413(2)	0.4321(16)	1.0(2)
*M*1: 0.44Ba5+0.56Y5	2*a*	0.2438(8)	0.725(2)	0.8961(15)	1.4(2)
*M*2: 0.47Ba6+0.53Y6	2*a*	0.9936(8)	0.095(2)	0.2287(14)	2.2(3)
*M*3: 0.52Ba7+0.48Y7	2*a*	0.7456(8)	0.751(2)	0.6054(13)	1.6(3)
M4: 0.58Ba8+0.42Y8	2*a*	0.4932(9)	0.3849(19)	0.2625(15)	1.9(2)
Disilicate 1 (*D*1), occupancies a:b = 0.61 : 0.39					
Si1a	2*a*	0.460(4)	0.666(7)	0.021(7)	0.7(2)
Si2a	2*a*	0.676(5)	0.964(7)	0.916(8)	0.7(2)
Si1b	2*a*	0.444(6)	0.670(11)	0.060(12)	0.7(2)
Si2b	2*a*	0.679(7)	0.928(12)	0.900(13)	0.7(2)
O1a	2*a*	0.554(7)	0.341(12)	0.942(13)	1.4(3)
O2a	2*a*	0.500(5)	0.092(11)	0.833(9)	1.4(3)
O3a	2*a*	0.629(7)	0.096(15)	0.998(15)	1.4(3)
O4a	2*a*	0.496(7)	0.155(10)	0.141(12)	1.4(3)
O5a	2*a*	0.336(7)	0.287(14)	0.039(13)	1.4(3)
O6a	2*a*	0.772(7)	0.006(12)	0.930(15)	1.4(3)
O7a	2*a*	0.350(6)	0.480(11)	0.259(12)	1.4(3)
O1b	2*a*	0.549(9)	0.320(18)	0.842(18)	1.4(3)
O2b	2*a*	0.474(8)	0.060(17)	0.926(18)	1.4(3)
O3b	2*a*	0.637(12)	0.09(3)	0.89(2)	1.4(3)
O4b	2*a*	0.569(7)	0.214(14)	0.115(15)	1.4(3)
O5b	2*a*	0.334(11)	0.32(2)	-0.04(2)	1.4(3)
O6b	2*a*	0.777(13)	-0.04(2)	0.88(2)	1.4(3)
O7b	2*a*	0.352(7)	0.332(14)	0.243(16)	1.4(3)
Disilicate 2 (*D*2), occupancies a:b = 0.60 : 0.40					
Si3a	2*a*	0.934(11)	0.79(2)	0.461(17)	0.7(2)
Si4a	2*a*	0.190(3)	0.551(7)	0.577(6)	0.7(2)
Si3b	2*a*	0.936(16)	0.79(3)	0.47(3)	0.7(2)
Si4b	2*a*	0.165(4)	0.539(9)	0.646(8)	0.7(2)
O8a	2*a*	0.868(6)	0.923(12)	0.515(12)	1.4(3)
O9a	2*a*	0.895(4)	0.673(10)	0.347(11)	1.4(3)
O10a	2*a*	1.053(5)	0.160(9)	0.416(10)	1.4(3)
O11a	2*a*	0.022(7)	0.858(10)	0.400(12)	1.4(3)
O12a	2*a*	0.286(9)	0.497(17)	0.576(17)	1.4(3)

O13a	2*a*	0.180(6)	0.718(12)	0.507(12)	1.4(3)
O14a	2*a*	0.158(7)	0.552(15)	0.749(15)	1.4(3)
O8b	2*a*	0.857(11)	0.90(2)	0.457(19)	1.4(3)
O9b	2*a*	0.906(11)	0.63(2)	0.52(2)	1.4(3)
O10b	2*a*	1.002(11)	0.351(19)	0.388(18)	1.4(3)
O11b	2*a*	-0.017(8)	0.794(17)	0.316(16)	1.4(3)
O12b	2*a*	0.265(9)	0.541(16)	0.667(17)	1.4(3)
O13b	2*a*	0.136(8)	0.709(15)	0.613(19)	1.4(3)
O14b	2*a*	0.125(8)	0.489(16)	0.802(14)	1.4(3)
Disilicate 3 (*D*3), occupancies a:b = 0.60 : 0.40					
Si5a	2*a*	0.202(4)	0.978(8)	0.186(8)	0.7(2)
Si6a	2*a*	0.939(4)	0.398(8)	0.013(7)	0.7(2)
Si5b	2*a*	0.180(5)	0.979(11)	0.212(10)	0.7(2)
Si6b	2*a*	0.926(6)	0.362(11)	0.031(12)	0.7(2)
O15a	2*a*	0.811(6)	0.375(11)	0.661(12)	1.4(3)
O16a	2*a*	0.757(6)	0.639(9)	0.780(10)	1.4(3)
O17a	2*a*	0.889(8)	0.491(15)	0.881(16)	1.4(3)
O18a	2*a*	0.754(5)	0.391(11)	0.945(10)	1.4(3)
O19a	2*a*	0.105(8)	0.942(13)	0.831(16)	1.4(3)
O20a	2*a*	0.941(5)	0.216(10)	0.006(9)	1.4(3)
O21a	2*a*	0.032(6)	0.458(11)	1.018(12)	1.4(3)
O15b	2*a*	0.806(10)	0.446(19)	0.611(18)	1.4(3)
O16b	2*a*	0.858(7)	0.644(13)	0.814(15)	1.4(3)
O17b	2*a*	0.876(11)	0.36(2)	0.874(17)	1.4(3)
O18b	2*a*	0.732(9)	0.462(17)	0.869(18)	1.4(3)
O19b	2*a*	0.099(11)	1.00(2)	0.863(19)	1.4(3)
O20b	2*a*	0.897(9)	0.202(16)	0.095(19)	1.4(3)
O21b	2*a*	0.024(12)	0.37(2)	1.00(2)	1.4(3)
Disilicate 4 (*D*4), occupancies a:b = 0.50(2) : 0.50(2)					
Si7a	2*a*	0.686(10)	0.521(12)	0.311(17)	0.7(2)
Si8a	2*a*	0.438(9)	0.093(14)	0.451(17)	0.7(2)
Si7b	2*a*	0.685(10)	0.541(12)	0.307(17)	0.7(2)
Si8b	2*a*	0.442(9)	0.074(14)	0.461(17)	0.7(2)
O22a	2*a*	0.514(5)	0.436(11)	0.534(10)	1.4(3)
O23a	2*a*	0.623(7)	0.579(14)	0.690(14)	1.4(3)
O24a	2*a*	0.656(10)	0.459(18)	0.15(2)	1.4(3)
O25a	2*a*	0.773(6)	0.604(12)	0.308(10)	1.4(3)
O26a	2*a*	0.615(10)	0.613(19)	0.399(18)	1.4(3)
O27a	2*a*	0.496(5)	0.735(11)	0.560(11)	1.4(3)
O28a	2*a*	0.693(6)	0.347(11)	0.358(11)	1.4(3)
O22b	2*a*	0.460(8)	0.59(2)	0.512(18)	1.4(3)
O23b	2*a*	0.579(9)	0.465(16)	0.684(18)	1.4(3)
O24b	2*a*	0.679(7)	0.492(16)	0.133(18)	1.4(3)
O25b	2*a*	0.717(6)	0.712(12)	0.325(11)	1.4(3)
O26b	2*a*	0.598(8)	0.514(16)	0.386(17)	1.4(3)
O27b	2*a*	0.600(8)	0.735(19)	0.570(17)	1.4(3)
O28b	2*a*	0.756(8)	0.415(12)	0.354(15)	1.4(3)

SI7. Additional crystallographic information on Phase B

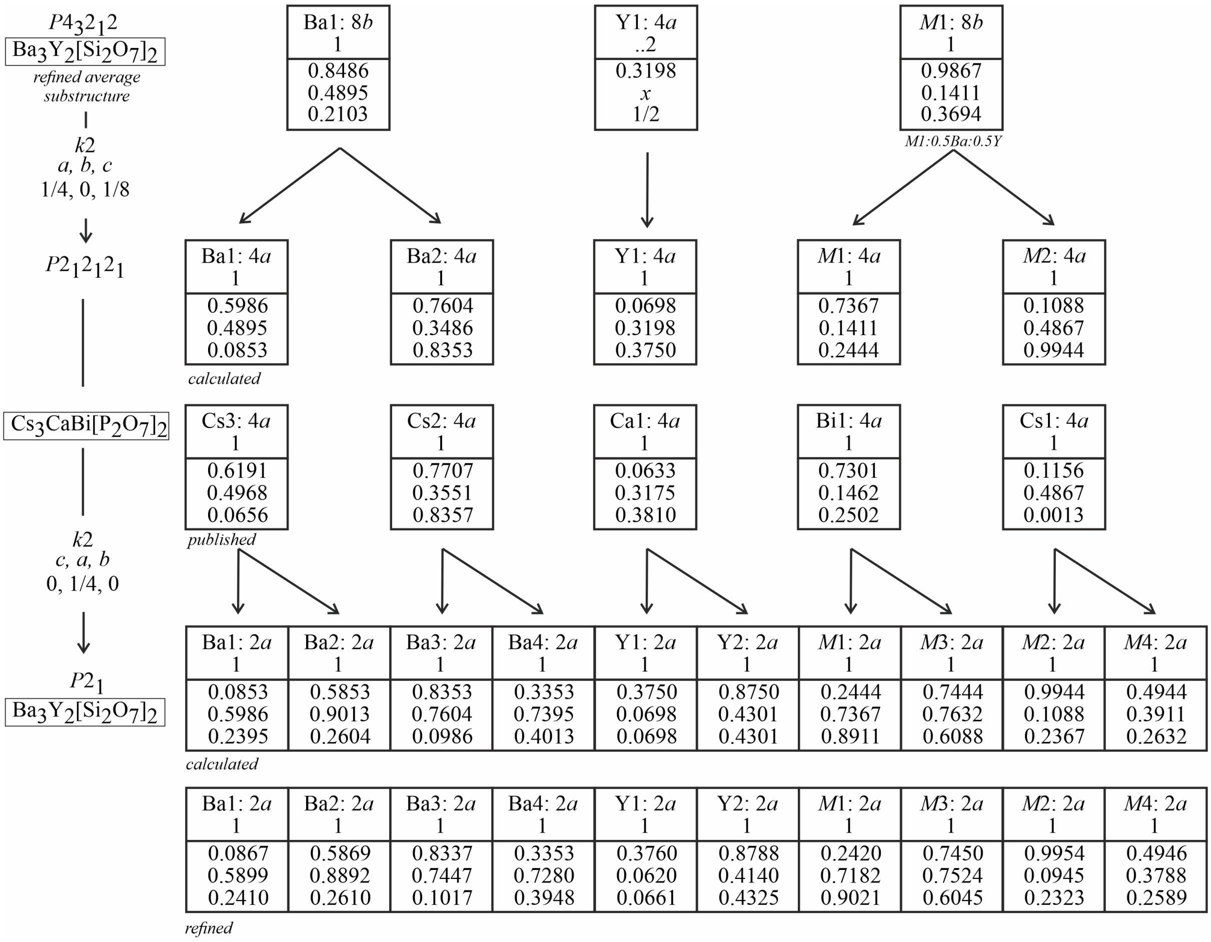

Figure SI21. Group-subgroup scheme in the Bärnighausen formalism[16–19] for the theoretical tetragonal subcell, orthorhombic $Ca_3BaBi[P_2O_7]_2$ (transformed from the standard setting via *a*,-*b*,-*c*, $00\frac{1}{2}$ to fit the calculated orientation) and the monoclinic refined model of $Ba_3Y_2[Si_2O_7]_2$. The indices for the klassengleiche (k) symmetry reductions, the unit cell transformations, as well as the evolution of the atomic parameters are given. Part 1: heavy atoms.

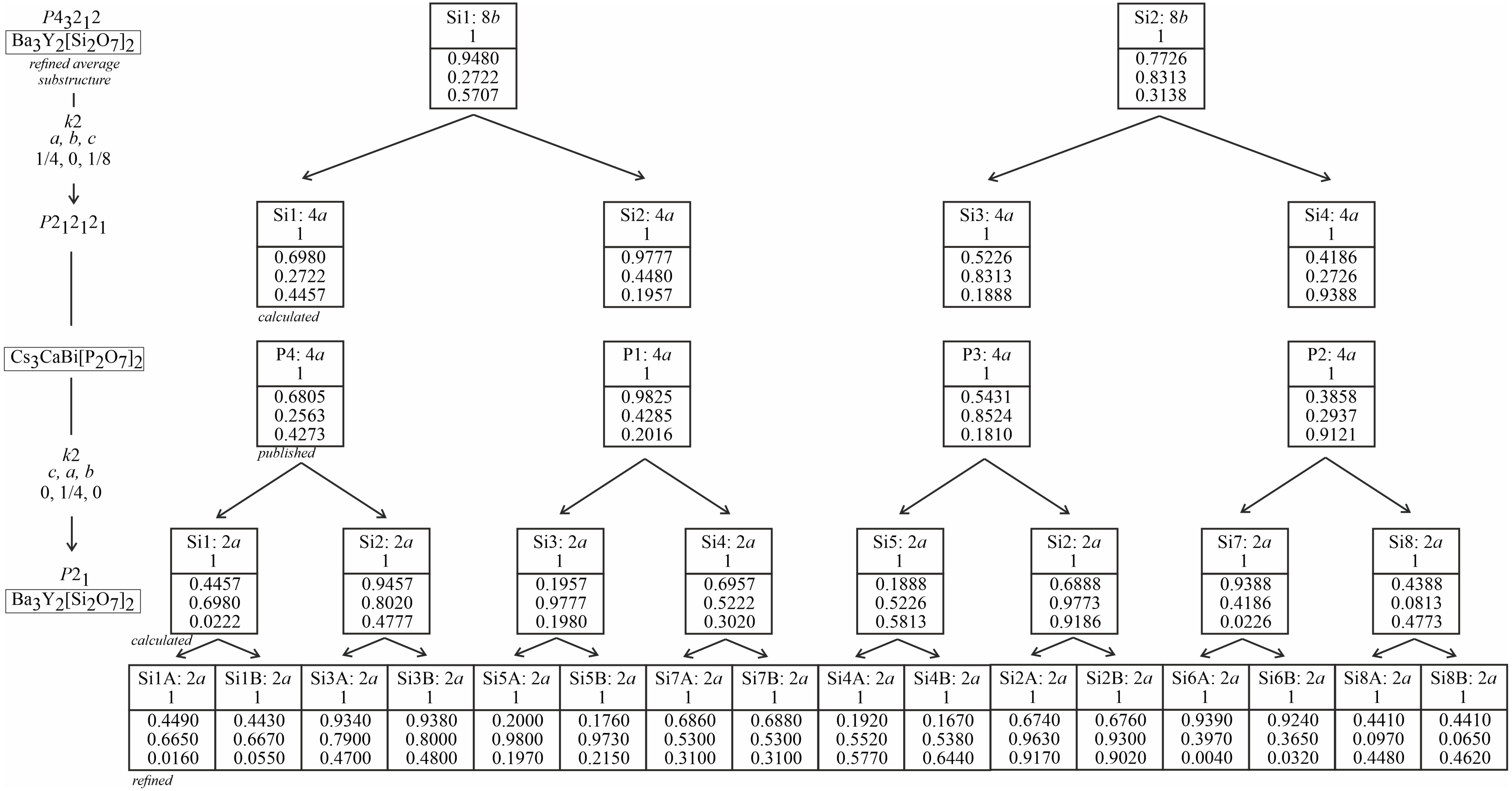

Figure SI22. Group-subgroup scheme in the Bärnighausen formalism[16–19] for the theoretical tetragonal subcell, orthorhombic $Ca_3BaBi[P_2O_7]_2$ (transformed from the standard setting via *a*,-*b*,-*c*, $00\frac{1}{2}$ to fit the calculated orientation) and the monoclinic refined model of $Ba_3Y_2[Si_2O_7]_2$. The indices for the klassengleiche (k) symmetry reductions, the unit cell transformations, as well as the evolution of the atomic parameters are given. Part 2: silicon atoms.

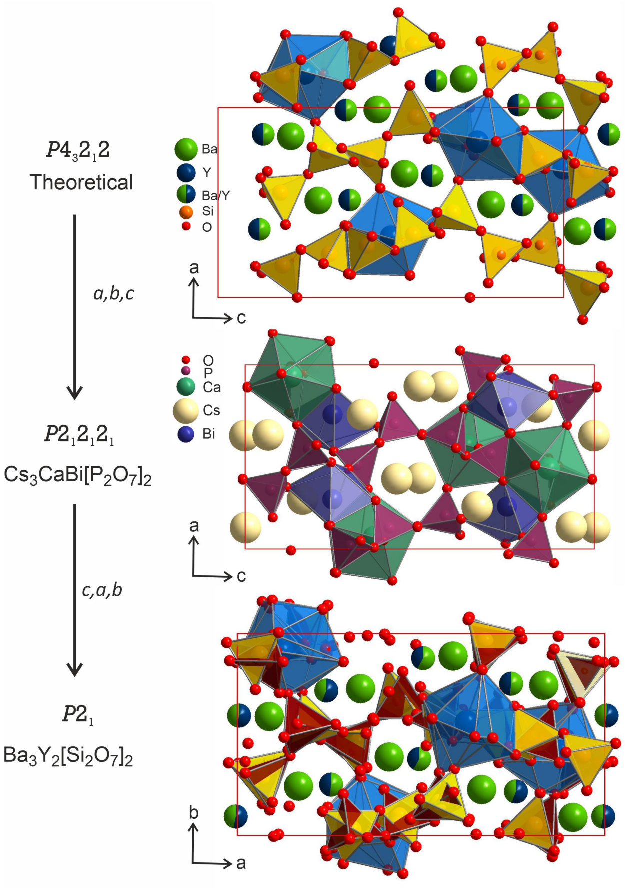

Figure SI23. Theoretical arisotype of $Ba_3Y_2[Si_2O_7]_2$ in supergroup $P4_32_12$ compared with the structure of $Cs_3CaBi[P_2O_7]_2$[20] in $P2_12_12_1$ and the refined structure of $Ba_3Y_2[Si_2O_7]_2$ in $P2_1$. Symmetry reduction route is shown on the left-hand site of the figure. In the structure of disilicate, Y- and Si-centred polyhedra drawn in blue and yellow/brown respectively. In the structure of diphosphate, Ca-, Bi-, and P-centred polyhedra are shown in teal, light-blue, and pink respectively.

Table SI13. Site coordinates and isotropic thermal parameters for the atoms in theoretical aristotype of $Ba_3Y_2[Si_2O_7]_2$ in supergroup $P4_32_12$ (Z = 4) based on combined Rietveld refinement of synchrotron and neutron TOF powder diffraction data recorded at room temperature (298 K).

Site	Wyck.	*x/a*	*y/b*	*z/c*
Ba1	8*b*	0.84863	0.48956	0.21037
Y1	4*a*	0.31984	0.31984	1/2
*M*1:0.5Ba2+0.5Y2	8b	0.98672	0.14116	0.36940
Si1	8*b*	0.94800	0.27221	0.57072
Si2	8*b*	0.77267	0.83139	0.31389
O1	8*b*	0.28591	0.16397	0.89225
O2	8*b*	0.42438	0.70621	0.96863
O3	8*b*	0.37047	0.12537	0.62335
O4	8*b*	0.14961	0.75169	0.98966
O5	8*b*	0.62167	0.55239	0.86662
O6	8*b*	0.29196	0.50023	0.97858
O7	8*b*	0.11638	0.32179	0.55490

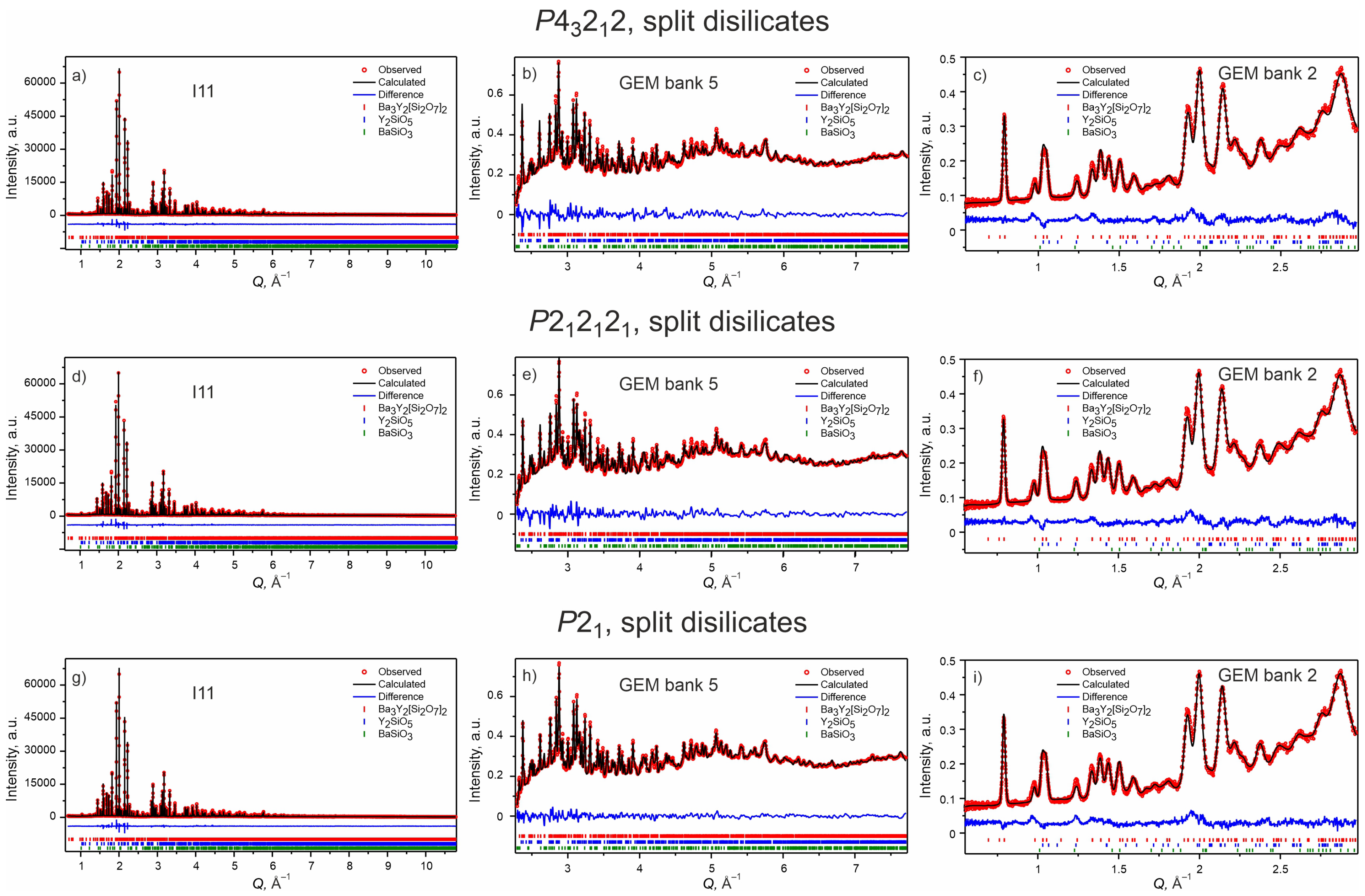

Figure SI24. Combined Rietveld refinement against synchrotron XRD (I11, Diamond Light Source, MAC detector, λ = 0.82387Å) and TOF neutron diffractions (Banks 3, 4 and 6 are not shown with respect to space) of $Ba_3Y_2[Si_2O_7]_2$ modelled in $P4_32_12$ (a-c), $P2_12_12_1$ (d-f), and $P2_1$ (g-i). Selected refinement parameters are listed in Table SI14. Lowering the symmetry achieves better fir to high-resolution NPD banks.

Table SI14. Comparison of R_{wp} and GOF for combined Rietveld refinements of different models of $Ba_3Y_2[Si_2O_7]_2$ in tetragonal, orthorhombic and monoclinic symmetries. Profile parameters were kept same as for the final refinement in Table SI9. Geometry of disilicate groups was restricted to be close to perfect. Lowering symmetry and splitting disilicate groups generates better fit against TOF neutron data.

Model / R_{wp}, %/χ2	$P4_32_12$		$P2_12_12_1$		$P2_1$
	No splitting	Split disilicates	No splitting	Split disilicates	Split disilicates
Total	6.62/3.39	5.70/2.92	6.51/3.33	5.73/2.94	5.19/2.92
SXRD	8.75/2.79	8.47/2.71	9.10/2.91	8.61/2.76	8.31/2.67
NHD bank 2	6.05/1.93	4.73/1.39	5.43/1.74	4.19/1.35	4.53/1.53
NPD bank 3	6.81/4.62	3.78/2.59	5.82/4.00	3.79/2.66	3.35/2.53
NPD bank 4	5.58/6.24	4.08/4.59	4.92/5.56	3.87/4.42	2.99/3.62
NPD bank 5	5.27/6.03	4.00/4.61	5.04/5.82	4.10/4.81	2.87/3.59
NPD bank 6	4.57/4.86	3.93/4.24	4.60/5.00	3.79/4.24	2.79/3.61

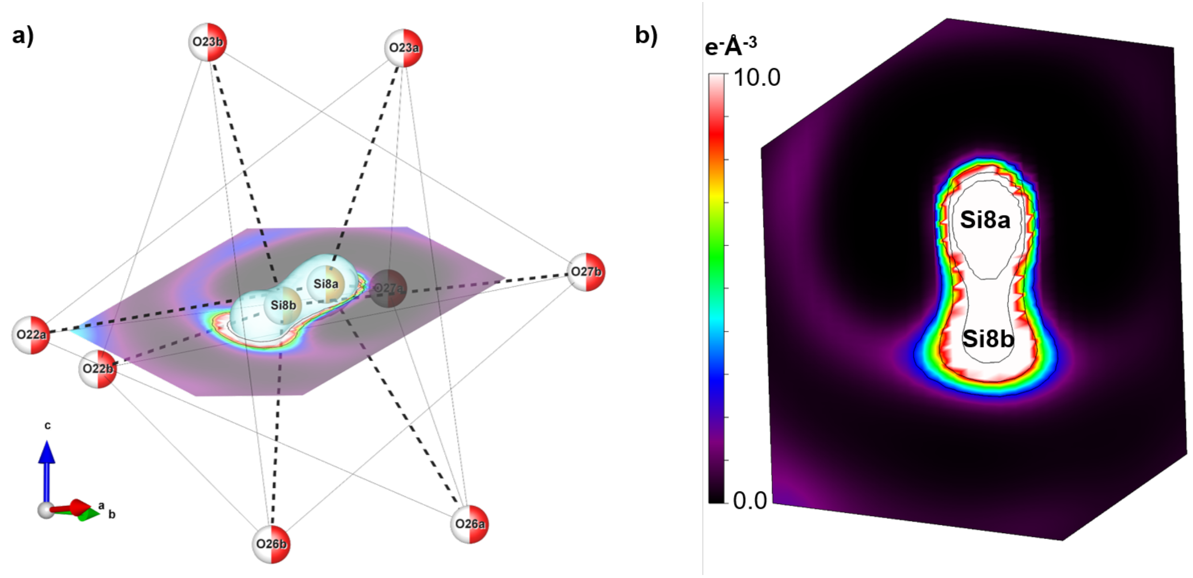

Figure SI25: Maximum entropy method calculations were used to evaluate the distribution of electron density across split silicon sites in $Ba_3Y_2[Si_2O_7]_2$ (phase B). A 300×300×300 voxel electron density map was generated *ab initio* from the observed SXRD data, providing a resolution of 20,044 voxels Å^{-3}; with an individual voxel representing electron density for a volume of 5×10^{-5} Å^3. **(a)** Electron density isosurface around the Si8a and Si8b positions generated by Maximum entropy method. The electron density isosurface is shown in light blue. Silicon is shown by the orange spheres and oxygen by the red spheres. Si–O bonds are shown by the dashed black lines and the SiO_4 polyhedra are shown by the grey lines. **(b)** The plane (0.7 -0.5 1), at a distance of 0.49 Å from the origin, has been selected to show a two-dimensional cross section of electron density that bisects the Si positions. A region of high electron density with two terminal nodules is observed; and supports the assigned positions of Si8a and Si8b in the average structure model from Rietveld refinement of SXRD.

SI8. Additional measurements

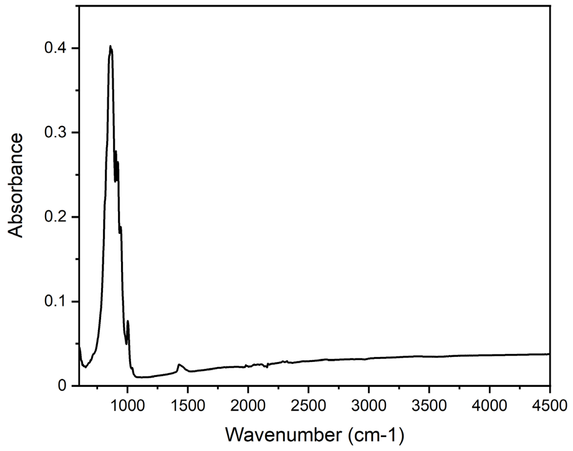

Figure SI26. IR spectrum of the $Ba_5Y_{13}[SiO_4]_8O_{8.5}$ sample. The absence of the characteristic peaks between 2700 - 3700 cm^{-1} indicates that no OH groups are present in the structure.

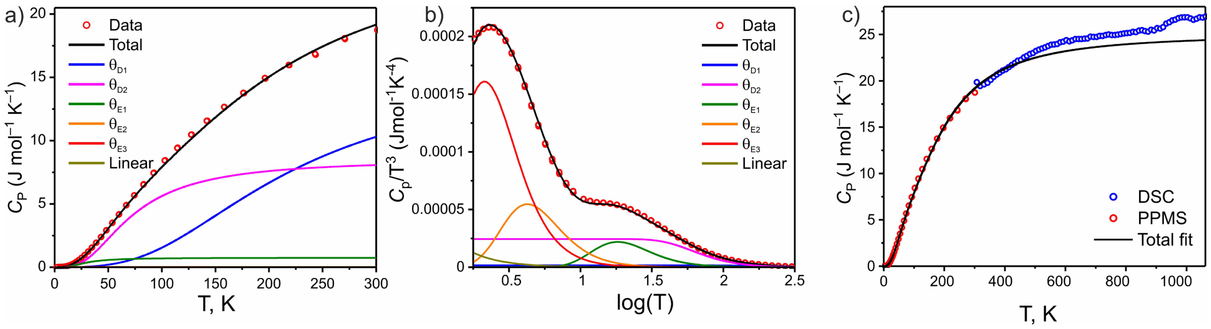

Figure SI27. Thermal properties of $Ba_5Y_{13}[SiO_4]_8O_{8.5}$. Heat capacity modelled as $C_p(T)$ (a) and $C_p/T^3(T)$ (b) using a linear combination of two Debye and three Einstein terms (Table SI15). (c) Heat capacity measured in a range from 1.8 to 1060°C. Red and blue circles correspond to PPMS and DSC collected data; black line indicates the fit obtained from the low temperature data.

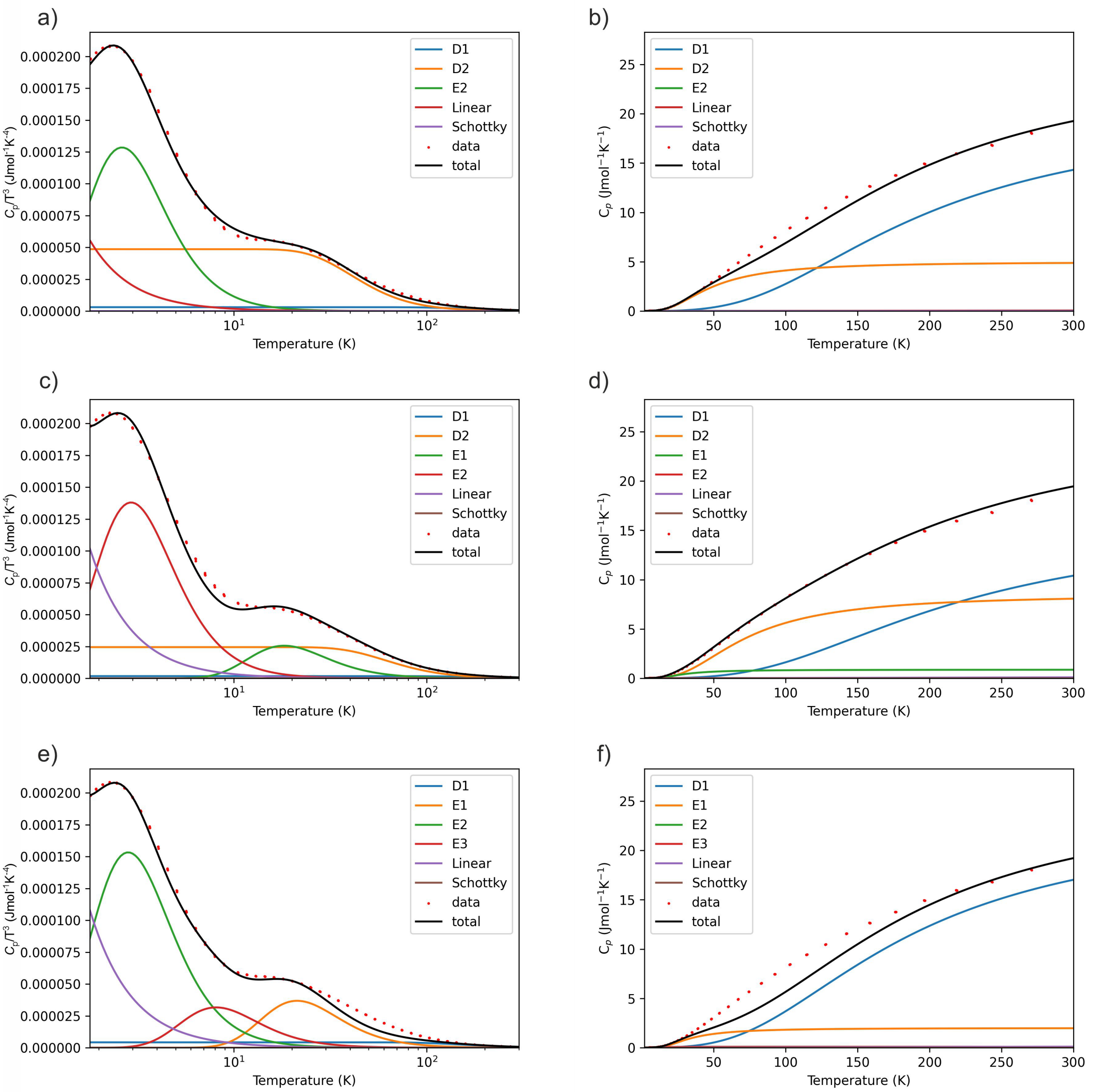

Figure SI28. Heat capacity of $Ba_5Y_{13}[SiO_4]_8O_8$ modelled as $C_p(T)$ (a) and $C_p/T^3(T)$ using a linear combination of different number of Debye and Einstein terms: (a-b) 2 Debye and 1 Einstein, (c-d) 2 Debye and 2 Einstein, (e-f) 1 Debye and 3 Einstein. The fits remain inferior to the 2 Debye and 3 Einstein model in Figure SI27.

Table SI15. The refined parameters for the heat capacity of $Ba_5Y_{13}[SiO_4]_8O_8$. The experimental data can be modelled by changing the values by ± 10 K and ± 2-5K for Debye respectively Einstein temperatures and consequently scaling the corresponding pre-factors.

**a1**	**θ_{D1}, K**	**a2**	**θ_{D2}, K**	**b1**	**θ_{E1}, K**	**b2**	**θ_{E2}, K**	**b3**	**θ_{E3}, K**	**γ, JK^{-2}mol^{-1}**
0.6287	910(10)	0.34	300(10)	0.03	90(5)	0.00095	21(5)	0.00035	10.5(2)	0.00004

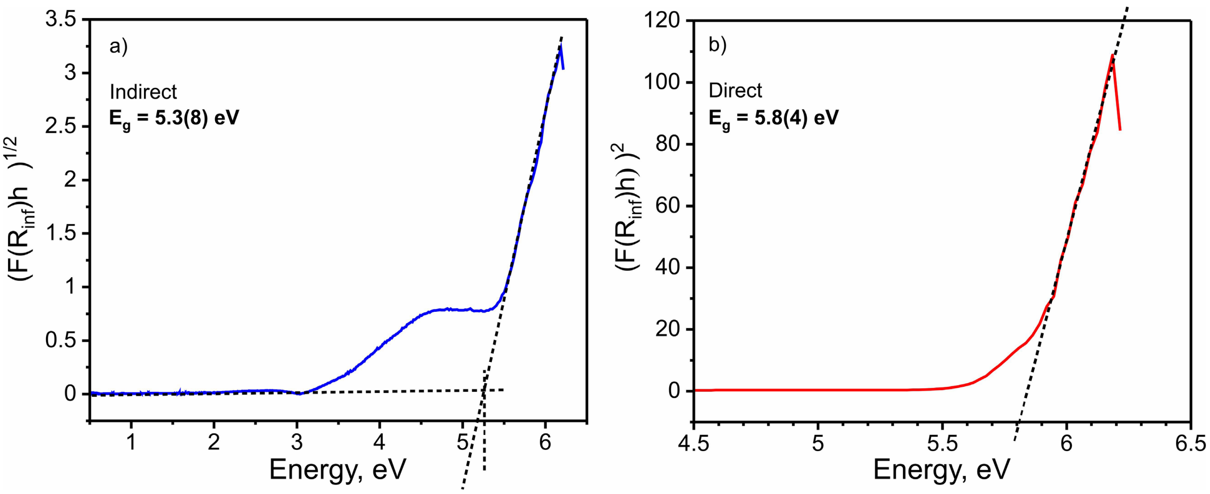

Figure SI29. UV-Vis spectra of $Ba_5Y_{13}[SiO_4]_8O_{8.5}$ phase.

SI9. References

1. A. A. Coelho, TOPAS and TOPAS-Academic: An Optimization Program Integrating Computer Algebra and Crystallographic Objects Written in C++: An. *J. Appl. Crystallogr.* 2018, **51**, 210–218. https://doi.org/10.1107/S1600576718000183.
2. J. Rodríguez-Carvajal, Recent Advances in Magnetic Structure Determination by Neutron Powder Diffraction. *Phys. B Condens. Matter.* 1993, **192**, 55–69. https://doi.org/https://doi.org/10.1016/0921-4526(93)90108-I.
3. Diamond - Crystal and Molecular Structure Visualization, Crystal Impact, Dr. H. Putz & Dr. K. Brandenburg GbR, Kreuzherrenstr. 102, 53227 Bonn, Germany, https://www.crystalimpact.de/diamond.
4. M. Roslova, S. Smeets, B. Wang, T. Thersleff, H. Xu, X. Zou, InsteaDMatic: Towards Cross-Platform Automated Continuous Rotation Electron Diffraction. *J. Appl. Crystallogr.* 2020, **53**, 1217–1224. https://doi.org/10.1107/S1600576720009590.
5. D. Zhang, P. Oleynikov, S. Hovmoller, X. Zou, Collecting 3D Electron Diffraction Data by the Rotation Method. *Z. Kristallogr.* 2010, **225**, 94–102. https://doi.org/10.1524/zkri.2010.1202.
6. W. Wan, J. Sun, J. Su, S. Hovmöller, X. Zou, Three-Dimensional Rotation Electron Diffraction: Software RED for Automated Data Collection and Data Processing. *J. Appl. Crystallogr.* 2013, **46**, 1863–1873. https://doi.org/10.1107/S0021889813027714.
7. T. Moriguchi, K. Mitsumoto, Y. Nishizawa, D. Yakeya, V. Jalli, A. Tsuge, Syntheses, Characterization and DFT Analysis of Two Novel Thiaheterohelicene Derivatives. *Cryst. Struct. Theory Appl.* 2016, **5**, 63–73. https://doi.org/10.4236/csta.2016.54006.
8. G. M. Sheldrick, SHELXT - Integrated Space-Group and Crystal-Structure Determination. *Acta Crystallogr. A* 2015, **71**, 3–8. https://doi.org/10.1107/S2053273314026370.
9. O. V. Dolomanov, L. J. Bourhis, R. J. Gildea, J. A. K. Howard, H. Puschmann, OLEX2: A Complete Structure Solution, Refinement and Analysis Program. *J. Appl. Crystallogr.* 2009, **42**, 339–341. https://doi.org/10.1107/S0021889808042726.
10. V. Petrícek, M. Dušek, L. Palatinus, Crystallographic Computing System JANA2006: General Features. *Z. Kristallogr.* 2014, **229**, 345–352. https://doi.org/10.1515/zkri-2014-1737.
11. S. van Smaalen, L. Palatinus, M. Schneider, The Maximum-Entropy Method in Superspace. *Acta Crystallogr. A* 2003, **59**, 459–469. https://doi.org/10.1107/S010876730301434X.
12. K. Momma, F. Izumi, VESTA 3 for Three-Dimensional Visualization of Crystal, Volumetric and Morphology Data. *J. Appl. Crystallogr.* 2011, **44**, 1272–1276. https://doi.org/10.1107/S0021889811038970.
13. S. Durdy, C. Hargreaves, M. Dennison, B. Wagg, M. Moran, J. A. Newnham, M. W. Gaultois, M. J. Rosseinsky, M. S. Dyer, The Liverpool Materials Discovery Server: A Suite of Computational Tools for the Collaborative Discovery of Materials. *Digit. Discov.* 2023, 1601–1611. https://doi.org/10.1039/d3dd00093a.
14. P. Makuła, M. Pacia, W. Macyk, How To Correctly Determine the Band Gap Energy of Modified Semiconductor Photocatalysts Based on UV–Vis Spectra. *J. Phys. Chem. Lett.* 2018, **9**, 6814–6817. https://doi.org/10.1021/acs.jpclett.8b02892.
15. Bruker. APEX III. 2019, Bruker AXS Inc., Madison, Wisconsin, USA.
16. H. Bärnighausen, Group-Subgroup Relations between Space Groups: A Useful Tool in Crystal Chemistry. *Commun. Math. Chem.* 1980, **9**, 139–175.
17. U. Müller, Kristallographische Gruppe-Untergruppe-Beziehungen Und Ihre Anwendung in Der Kristallchemie Crystallographic Group-Subgroup Relations and Their Use in Crystal Chemistry. *Z. Anorg. Allg. Chem* 2004, **630**, 1519–1537. https://doi.org/10.1002/zaac.200400250.
18. U. Müller, Relating Crystal Structures by Group–Subgroup Relations. In *International Tables for Crystallography. Vol. A1: Symmetry relations between space groups*; Wondratschek, H., Müller, U., Eds.; John Wiley & Sons: Chichester, 2011; pp 44–56.

https://doi.org/https://doi.org/10.1107/97809553602060000795.
19. U. Müller, *Symmetriebeziehungen Zwischen Verwandten Kristallstrukturen*; Vieweg + Teubner Verlag: Wiesbaden, Germany, 2012.
20. I. V. Zatovsky, N. Y. Strutynska, Y. A. Hizhnyi, V. N. Baumer, I. V. Ogorodnyk, N. S. Slobodyanik, I. V. Odynets, N. I. Klyui, New Complex Phosphates $Cs_3M^{II}Bi(P_2O_7)_2$ (M^{II}-Ca, Sr and Pb): Synthesis, Characterization, Crystal and Electronic Structure. *Dalt. Trans.* **2018**, *47*, 2274–2284. https://doi.org/10.1039/c7dt04505k.
